# A critical comparison of technologies for a plant genome sequencing project

**DOI:** 10.1093/gigascience/giy163

**Published:** 2019-01-09

**Authors:** Pirita Paajanen, George Kettleborough, Elena López-Girona, Michael Giolai, Darren Heavens, David Baker, Ashleigh Lister, Fiorella Cugliandolo, Gail Wilde, Ingo Hein, Iain Macaulay, Glenn J Bryan, Matthew D Clark

**Affiliations:** 1 Technology Development, Earlham Institute, Norwich Research Park, Norwich NR4 7UZ, UK; 2 Department of Cell and Developmental Biology, John Innes Centre, Norwich Research Park, Norwich NR4 7UH, UK; 3 Cell and Molcular Sciences, The James Hutton Institute, Invergowrie, Dundee DD2 5DA, UK; 4 The New Zealand Institute for Plant & Food Research Limited, Palmerston North 4442, New Zealand; 5 Department of Life Sciences, Natural History Museum, Cromwell Road, London WC2 5BD, UK

**Keywords:** assembly, long reads, short reads, optical mapping, Pacific Biosciences, PacBio, 10x Genomics

## Abstract

**Background:**

A high-quality genome sequence of any model organism is an essential starting point for genetic and other studies. Older clone-based methods are slow and expensive, whereas faster, cheaper short-read–only assemblies can be incomplete and highly fragmented, which minimizes their usefulness. The last few years have seen the introduction of many new technologies for genome assembly. These new technologies and associated new algorithms are typically benchmarked on microbial genomes or, if they scale appropriately, on larger (e.g., human) genomes. However, plant genomes can be much more repetitive and larger than the human genome, and plant biochemistry often makes obtaining high-quality DNA that is free from contaminants difficult. Reflecting their challenging nature, we observe that plant genome assembly statistics are typically poorer than for vertebrates.

**Results:**

Here, we compare Illumina short read, Pacific Biosciences long read, 10x Genomics linked reads, Dovetail Hi-C, and BioNano Genomics optical maps, singly and combined, in producing high-quality long-range genome assemblies of the potato species *Solanum verrucosum*. We benchmark the assemblies for completeness and accuracy, as well as DNA compute requirements and sequencing costs.

**Conclusions:**

The field of genome sequencing and assembly is reaching maturity, and the differences we observe between assemblies are surprisingly small. We expect that our results will be helpful to other genome projects, and that these datasets will be used in benchmarking by assembly algorithm developers.

Developments in high-throughput sequencing have revolutionized genetics and genomics, with lower costs leading to an explosion in genome sequencing project size [1] and number of species [2]. Genomes from many diverse organisms have been sequenced, from marsupials to microbes, plants, phytoplankton, and fungi, among many others [3]. For a while it has been feasible for a single lab to sequence and *de novo* assemble a complex genome (e.g., [4]).

The existence of very high-quality references [5, 6] has made the human genome popular for demonstrating new sequencing technologies and assembly algorithms. The human genome has now been sequenced and assembled using various technologies including Sanger, 454, IonTorrent, Illumina, Pacific Biosciences (PacBio), 10x Genomics, and even nanopore sequencing technologies [7–12]. Hybrid approaches that combine complementary technologies, e.g., PacBio and BioNano, have also been used [13].

However, the human genome is not representative of all eukaryotic genomes; plant genomes in particular are typically more repetitive (including multi-kilobase long retrotransposon elements as well as even longer regions comprising “nested” transposon insertions). Plant biology also poses challenges for the isolation of high-quality high-molecular-weight DNA due to strong cell walls, co-purifying polysaccharides, and secondary metabolites that inhibit enzymes or directly damage DNA [14]. Thus, technologies that work well on vertebrate genomes may not work well for plants [15]. For these reasons, slow and expensive clone-based minimal tiling path sequencing approaches have persisted in plants [16, 17] long after faster, cheaper short-read whole-genome assemblies were first demonstrated for vertebrate genomes [18]. In addition to increased genome repetitiveness and size, polyploidy is common in plants (especially key crops such as cotton, brassicas, wheat, and potatoes) as are high levels of heterozygosity, especially where inbreeding is problematic due to generation times [19] or the plants are obligate outcrossers.

Plant biology poses some additional challenges for the isolation of high-quality high-molecular-weight DNA. Plant cells possess strong rigid cell walls not broken by the addition of a detergent and, when physically breaking the cell wall, the DNA can be sheared, rendering the isolation of high-molecular-weight DNA problematic. A large proportion of the DNA in a plant cell can be from organelles (mitochondrial and chloroplast) [20], which are high copy number and large, e.g., the mitochondrial genome is 453  kbp in wheat [21] but only 16  kbp in human [22]. Plants are also rich in polysaccharides that can co-purify with DNA, and they produce secondary metabolites to protect themselves from herbivores [14].

Plant genomes also vary hugely in size, from 61  Mbp (*Genlisea tuberosa*, a member of the bladderwort family [23]) to 150  Gbp (*Paris japonica*, a relative of lilies [24]). It is still nontrivial to design a *de novo* assembly project that involves an ensemble of technologies. Each platform comes with its own input requirements, computational requirements, quality of output, and, of course, labor and materials costs. Our results can be used as guidance for further sequencing assembly projects and provide a basis for comparative genome studies, as each sequencing strategy and assembly method has its own biases.

Here, we compare several practical *de novo* assembly projects of a Mexican wild potato species, *Solanum verrucosum*. We chose this genome because *S. verrucosum* is a self-compatible, diploid, tuber-bearing, wild potato species that we inbred further to produce the line Ver-54. The estimated genome size based on *k*-mer content is 722 Mbp. In addition, recent cytogenetic and molecular studies have shown it likely represents a genome donor to Mexican allopolyploid potatoes [25, 26] and, as such, is taxonomically distant from the genetically characterized cultivars and landraces, although it has been classified into the same larger phylogenetic potato clade (Clade 4) as cultivated potatoes [27]. The Mexican allopolyploids in Series Longipedicellata and Demissa have very high levels of resistance to *Phytophthora infestans* (encoded by several R-genes) as does *S. verrucosum*. Thus, the *S. verrucosum* genome can be a highly useful genetic resource and a “potato model” for forward/reverse genetic studies relating to its high level of blight resistance, its unusually high level of self-fertility, and because it produces tubers, albeit small inedible ones. The Solanaceae, or nightshades, are a family containing many economically important, and previously sequenced, plants including potato *Solanum tuberosum* [28], tomato *Solanum lycopersicum* [29], aubergine *Solanum melongena* [30], and pepper *Capsicum annuum* [31]. These related species genomes can provide information about genome organization in the Solanaceae and allow comparative genomic studies.

## Results

The results of this study are presented in two parts. In the first part, we compare several short-read (Illumina) to long-read (PacBio) based assemblies. These represent the simplest type of sequencing projects that are often undertaken. We then choose one each of the Illumina-based and one PacBio-based assembly and, in the second part, use various different combinations of longer-range scaffolding data from newer technologies, namely, *in vitro* Hi-C (Dovetail), optical mapping (BioNano Genomics) to increase continuity. Finally, we compare these approaches to the read clouds (10x Genomics Chromium) technology, which promises short-read assembly and longer-range scaffolding simultaneously. Validating the assemblies for sequence and scaffolding accuracy, we find strengths and weaknesses and that methods differ hugely in their DNA, time, computational requirements, and cost.

### Contig assembly and scaffolding

**Figure 1:**
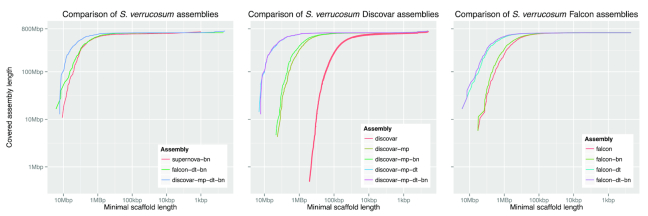
Comparison of contig/scaffold lengths and total assembly sizes of the various *S. verrucosum* assemblies.

The first stage of an assembly is to piece together reads to form long contiguous sequences, or *contigs* for short. These contigs can be ordered and oriented using longer-range information such as jumping/mate-pair libraries. Throughout this article we will refer to different contig assemblies that have been scaffolded. We use a naming convention that shows all of the steps used to construct the assembly. Each assembly name contains the steps used in order, separated by a hyphen. For example, the discovar-mp-dt-bn assembly is the discovar contig assembly scaffolded first with mate-pairs, then Dovetail and finally BioNano. An overview of the assmebly results is presented in Fig. 1.

#### Illumina contig assembly

Two libraries were constructed for Illumina assembly. The first is a PCR-free library with insert size 500 bp (±40%) that was sequenced with 250 bp paired-end reads on a single Illumina HiSeq 2500 run. We refer to this below as the Discovar library. The coverage of the library was 120×. The second library is a PCR-free “Tight and Long Library” (TALL) with insert size 650  bp (±20%) sequenced with 100  bp and 150  bp paired-end reads on two Illumina HiSeq 2500 runs. The coverage of this library was 135×.

We analyzed the TALL library reads with preqc, part of the SGA assembler [32], and giving a genome size estimate at 702  Mbp, while the same analysis on the Discovar library yielded 722  Mbp. The latter agrees better with the 727  Mbp size of the potato genome assembly [28].

The TALL library was assembled with ABySS (ABySS, RRID:SCR_010709)[33] (*k*-mer size 113) and the Discovar library using Discovar*de novo* (Discovar, RRID:SCR_016755) [34] producing contig assemblies discovar and abyss, respectively.

The results for these two Illumina assemblies are similar in contiguity and shown in Table 1. However, while ABySS assembled about 8% longer total length, the number of small contigs was larger, leading to very similar contig N50 to Discovar. One additional feature was that AbySS performed more scaffolding using the paired-end data but did not fill many of the introduced gaps, leading to about 100 times higher percentage of N bases than Discovar. These assemblies are more contiguous than the equivalent contig assemblies of the *S. tuberosum* genome where the reported contig N50 from paired-end reads is 22.4  kbp [28].

**Table 1:** Assembly statistics of Illumina and PacBio assemblies, with a minimum contig/scaffold size of 1 kbp

Assembly	Number of contigs	N50 (kbp)	Max length (kbp)	Total length (Mbp)
abyss	33,146	75	642	702
abyss-mp	21,376	331	2,288	712
discovar	25,216	77	498	646
discovar-mp	8,074	858	4,266	665
hgap	5,446	585	4,876	716
canu	8,138	290	4,701	722
falcon	2,442	712	5,738	659

abyss uses the TALL library; discovar uses the Discovar library; and hgap, canu, and falcon use the PacBio library. For a more comprehensive summary, see Supplementary Table S3.1.

#### Illumina scaffolding

A Nextera long mate-pair (LMP) library was made with insert size 10,000  bp (±20%) and sequenced on two lanes of an Illumina MiSeq with fragment size 500  bp and 300  bp reads. The total coverage of the LMP library was 15× after we had filtered out duplicates 23.4% *ofreads*, reads that did not contain a Nextera adapter or were too short to be useful.

We scaffolded both the discovar and abyss assemblies separately using Soapdenovo2 (soapdenovo2,RRID:SCR_014986) [35], producing discovar-mp and abyss-mp, respectively. The contiguity of both was increased significantly as shown in Table 1. Here the discovar-mp scaffolds were slightly better, so we used this assembly to take forward for longer-range scaffolding with other data types.

#### PacBio assembly

A gel size selected PacBio library with fragment lengths of at least 20  kbp was made according to the manufacturer’s instructions. The library was sequenced using a PacBio RSII instrument and P6C4 chemistry. We sequenced 65 single-molecule real-time (SMRT) cells total, each giving about 500  MB of data and a total coverage of 50×. The N50 of the fragments was 13,499  bp and total number of reads 9 7,68,980.

We conducted three long-read assemblies on the same data using HGAP3 [36], part of SMRT-analysis (version 2.3.0p5) (SMRT-Analysis, RRID:SCR_002942), Canu (Canu, RRID:SCR_015880) [37] (version 1.0), and Falcon (Falcon, RRID:SCR_016089) [38] (version 0.3.0), producing the hgap, canu, and falcon assemblies, respectively. The assembly statistics for each are shown in Table 1. Another long-read assembler that we chose not to use because it does not include any error correction is miniasm [39]. This is a fast, lower computational power alternative to the ones that we used here and is useful for many purposes, e.g., empirical testing of long-read assemblies.

The Canu assembly was made with reads that were first error-corrected by the HGAP3 pipeline because the first attempt using raw reads resulted in an excessive amount of small scaffolds and a genome size more than 50% longer than expected.

The canu and hgap assemblies contain slightly more sequence content (as measured by the total length of the assembly) and a lower percentage of unknown bases (as measured by the percentage of bases denoted by N) than the short-read assemblies. This may be due to their capturing of additional difficult sequences, especially repeat elements that short-read assemblies are known to have problems traversing. The falcon assembly has the highest N50, and canu is closest to the estimated genome length. Falcon also produced 9.9  Mbp of alternate contigs, likely from residual heterozygosity, which will be useful for interpreting downstream genetic results, e.g., forward and reverse genetic screens. We also found this assembly was easier and faster to run than HGAP3. We also found the base-pair accuracy of canu read correction to be lower than HGAP3 read correction. For these reasons, we chose the falcon assembly to take forward to hybrid scaffolding. We first polished it using Quiver as part of SMRT analysis (version 2.3.0p5).

### Longer-range scaffolding

To achieve higher contiguity, newer technologies have been developed to complement the previous methods and, in some cases, each other. In this section, we investigate use of longer-range scaffolding methods to increase the contiguity of the Illumina discovar-mp assembly and the falcon PacBio assembly. We also investigate the 10x Genomics Chromium platform, an integrated solution that can be used to generate short Illumina reads with long-range positional information.

#### Dovetail

Dovetail Genomics provides a specialized library preparation method called Chicago and an assembly service using a custom scaffolder called HiRise. The Chicago library preparation technique is based on the Hi-C method, producing deliberately “chimeric” inserts linking DNA fragments from distant parts of the original molecule [40]. This is followed by standard Illumina paired-end sequencing of the inserts. Since the separation of the original fragments follows a well-modeled insert size distribution, the scaffolder is able to join contigs to form scaffolds spanning large distances, even up to 500  kbp [40].

Dovetail Genomics, LLC (Santa Cruz, CA) received fresh leaf material from us from which they constructed a Chicago library. This was sequenced at Earlham Institute using Illumina 250  bp paired-end reads. The total read coverage of the Chicago library was 105×. Dovetail used their HiRise software to further scaffold the discovar-mp assembly, increasing the N50 from 858  kbp to 4,713  kbp, and the falcon assembly, increasing the N50 from 712  kbp to 2,553  kbp. These assemblies are called discovar-mp-dt and falcon-dt, respectively.

#### BioNano

The BioNano Genomics Irys platform constructs a physical map using very large DNA fragments digested at known sequence motifs with a specific nicking enzyme to which a polymerase adds a fluorescent nucleotide. The molecules are scanned, and the distance between nicks generates a fingerprint of each molecule that is then used to build a whole-genome physical map. Sequence-based scaffolds or contigs can be integrated by performing the same digestion *in silico* then ordering and orienting the contigs according to the physical map [41].

We collected BioNano data from 16 runs by repeatedly running the same chip. After filtering fragments less than 100  kbp, the yield varied from 0.8  Gb to 25.8  Gb, with the earlier runs yielding more whereas the molecule N50 was higher in later runs (ranging from 135  kbp to 240  kbp). The total yield of BioNano data was 252  Gbp, which is roughly equivalent to 350× coverage.

We performed hybrid scaffolding on the discovar-mp and falcon assemblies. The *in silico* digest suggested a label density of 8.1/100  kbp for discovar-mp and 8.4/100  kbp for falcon while the actual observed density was only 6.8/100  kbp. We used the BioNano pipeline (v2.0) (BioNano Irys, RRID:SCR_016754) to scaffold discovar-mp, increasing the N50 from 858  kbp to 1,260  kbp, and falcon, increasing the N50 from 710 kbp to 1,500  kbp. These assemblies are called discovar-mp-bn and falcon-bn, respectively.

#### 10x Genomics

10x Genomics provides an integrated microfluidics based platform for generating linked reads (a cloud of non-contiguous reads with the same barcode from the same original DNA molecule) and customized software for their analysis [11]. Large fragments of genomic DNA are combined with individually barcoded gel beads into micelles in which library fragments are constructed and then sequenced as a standard Illumina library. Using the barcodes, the reads from the same gel bead can be grouped together.

Unlike the previous two longer-range scaffolding approaches, the 10x Genomics platform constructs a new paired-end library that can be sequenced and then assembled into large scaffolds by one assembly program: Supernova.

A 10x Genomics Chromium library was made according to manufacturer’s instructions, and a lane of Illumina HiSeq 250  bp paired-end reads were generated with a coverage of about 92×. Supernova (version 1.1.1) (Supernova, RRID:SCR_016756) produced the supernova assembly with length 641  Mbp and a scaffold N50 of 2.33  Mbp. Trimming reads back to 150  bp or reducing sequencing depth to 56×, which are the read length and depth recommended by 10x Genomics, generated very similar results (see Supplementary Section 2.3) compared to the ones reported above.

#### Hybrid scaffolding

It is possible to iteratively combine these longer-range scaffolding approaches. We tested several hybrid approaches using the discovar, falcon, and supernova assemblies. For example, the discovar-mp assembly was scaffolded using Dovetail and then BioNano producing discovar-mp-dt-bn with an N50 of 7.0  Mbp, the highest contiguity of any assembly reported here. The falcon assembly when scaffolded with both produced scaffolds with an N50 of only 3.09  Mbp, lower than with BioNano alone. Finally, we scaffolded the supernova assembly with BioNano producing supernova-bn, which increased the N50 from 2.33  Mbp to 2.85  Mbp.

Most scaffolding steps add gaps of unknown sequence, so we also used long reads from PacBio to scaffold and to perform “gapfilling” on the assemblies, replacing regions of unknown sequence (N stretches) with a PacBio consensus sequence. This also presents an opportunity to use lower coverage PacBio data to improve an Illumina assembly, which may be more cost effective than a *de novo* assembly using PacBio. PBJelly (version 15.2.20) [42] was used to perform gapfilling using only 10 SMRT cells of PacBio data (8× depth). The Supernova assembly increased in size from 641  Mbp to 671  Mbp, and N50 from 2.33  Mbp to 2.64  Mbp, and the amount of Ns present reduced from 7.58% to 5.14%. The discovar-mp-dt assembly increased in size from 656  Mbp to 680  Mbp and N50 from 4.69  Mbp to 4.87  Mbp, with Ns reduced from 3.03% to 1.28%. However, how gaps and percentage Ns are generated differs between assembly methods (see Discussion section).

## Assembly evaluation

Achieving a genome assembly with high levels of contiguity is potentially useless if it does not faithfully represent the original genome sequence. We assessed errors in assemblies by comparison to the raw data used to make the assemblies, as well as measuring gene content, local accuracy (Bacterial Artificial Chromosome (BAC) assemblies), and long-range synteny with the close relative *S. tuberosum*.

### *k*-mer content

**Figure 2:**
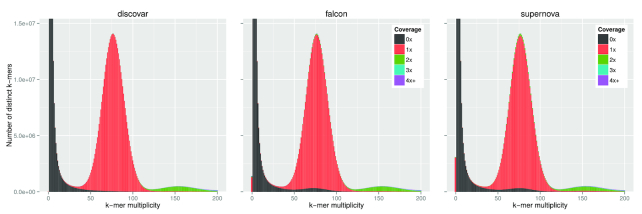
*k*-mer spectra plots from the *k*-mer Analysis Toolkit comparing three *S. verrucosum* contig assemblies. The heights of the bars indicate how many *k*-mers of each multiplicity appear in the raw Discovar reads. The colors indicate how many times those *k*-mers appear in the respective assemblies with black being zero times and red being one time. A colored bar at zero multiplicity indicates *k*-mers appearing in the assembly that do not appear in the reads. The Falcon assembly has been polished with the Illumina reads using Pilon to reduce the effect of using a different sequencing platform.

Analysis of the *k*-mer content of an assembly gives a broad overview of how well the assembly represents the underlying genome. We used the PCR-free Illumina Discovar library as our reference for the *k*-mer content of the genome. Due to the high accuracy of the reads, we expect the *k*-mer spectra for a library to form a number of distributions that correspond to read errors, non-repetitive, and repetitive content in the genome. These distributions can be seen by observing only the shapes and ignoring the colors in Fig. 2. The reader is referred to the *k*-mer Analysis Toolkit (KAT, RRID:SCR_016741) documentation for further details [43].

In Fig. 2 we compare the *k*-mer contents of the three contig assemblies—discovar, falcon, and supernova—to the Discovar library. To minimize the effects of the differences between Illumina and PacBio sequencing error profiles, the falcon assembly has been polished with the Illumina reads using Pilon [44] (see Supplementary Figure S3.1 for the unpolished plot).

The small red bar on the origin in some plots shows content that appears in the assembly but not in the Illumina reads. The discovar assembly is very faithful to the content in the library. The black area denotes sequences in the reads but not in the assembly; those clustering at the origin are predicted sequence errors in the reads, the small amount between 50 and 100 on the *x*-axis is sequence missing from the assembly. The dominant red peak (1×, around multiplicity 77), which is the vast majority of all assemblies here, contains content in the Illumina reads that appears once in the assembly (homozygous sample). Green areas on top of the main peak in Falcon and Supernova represent possible duplications in the assembly, whereas the green (2×) small peak to the right of the main peak is probably true duplicates—as these sequences are present twice in the assembly and at twice the expected read counts. At the main peak (*k*-mer multiplicity 77), the amount of potentially duplicated content in the assemblies (i.e., number of *k*-mers appearing more than once in the assembly) is 0.66% in falcon, 1.3% in supernova, and 0.15% in discovar.

### Gene content

We assessed the gene content of the three most contiguous assemblies—discovar-mp-dt-bn, falcon-dt-bn, and supernova-bn—using two datasets. The first is with Benchmarking Universal Single-Copy Orthologs (Busco) and its embryophyta_odb9 (plants) dataset (BUSCO, RRID:SCR_015008) [45] and the second is all the predicted transcript sequences from the *S. tuberosum* genome [28].

**Figure 3:**
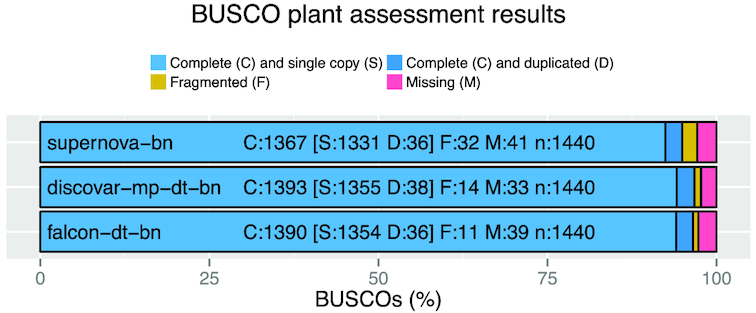
Busco analysis of supernova-bn, discovar-mp-dt-bn, and falcon-dt-bn using the plant gene dataset.

We found that each of the three assemblies shows at least 95% of Buscos as complete, with just a small difference of only 2% to 3% missing. The results are shown in Fig. 3.

**Figure 4:**
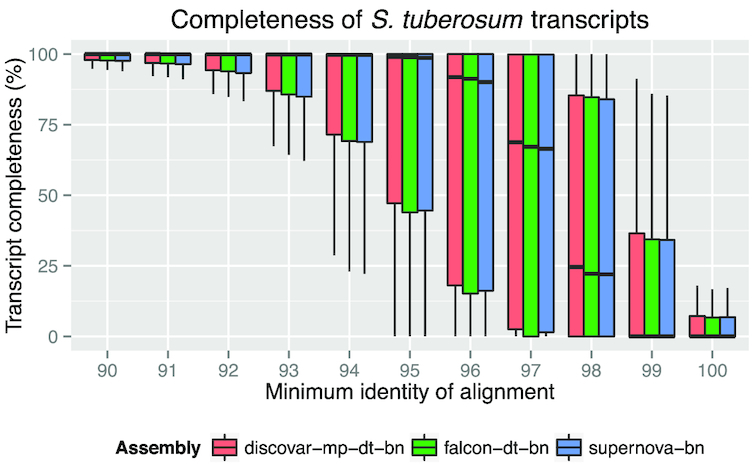
Box and whisker plot showing completeness of the *S. tuberosum* transcripts in supernova-bn, discovar-mp-dt-bn, and falcon-dt-bn with various levels of minimum percentage identity.

We aligned the *S. tuberosum* representative transcript sequences to each genome assembly using Basic Local Alignment Search Toolusing (Blast) [46] and then measured how much of each transcript sequence was represented in the assembly according to various minimum percentage identity cutoffs. As expected when comparing between species, as the threshold approaches 100% nucleotide identity, the transcript completeness drops closer to zero. Using a threshold between 96% and 98%, we find the median transcript completeness is highest in discovar-mp-dt-bn, followed by falcon-dt-bn, and then supernova-bn. However, the difference between the assemblies is small. Figure 4 shows a box and whisker plot of completeness of the representative transcript sequences.

### Local accuracy

As BACs are easier to assemble due to smaller size and a much more limited amount of repetitive DNA content than a whole genome, we assessed the performance of our three assemblies at a local scale using BAC assemblies. We randomly selected, sequenced, and assembled 96 BAC clones from the *S. verrucosum* BAC library. We chose 20 high-quality BAC assemblies (single scaffolds/contigs with Illumina or PacBio) to measure the accuracy of the whole-genome assemblies.

We used dnadiff [47] to compare the BAC sequences to the supernova-bn, discovar-mp-dt-bn, and falcon-dt-bn assemblies, finding sequence identities of 99.40%, 99.97%, and 99.87%, respectively. As in the previous section, the discovar-mp-dt-bn assembly shows the highest accuracy, with supernova-bn the lowest, though the differences are small.

**Figure 5:**
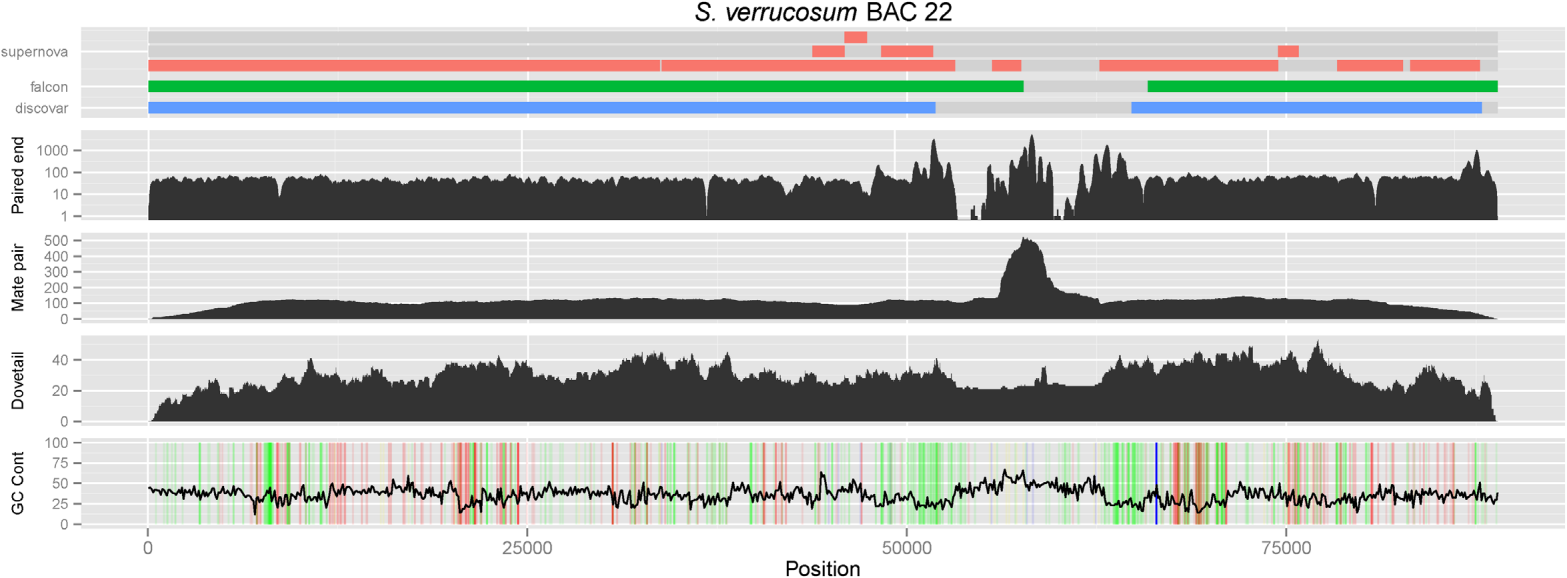
A difficult region of the genome that is contiguously assembled with a PacBio BAC but in none of our whole-genome assemblies. The region was correctly scaffolded by Dovetail. The figure shows various alignments and information with respect to the BAC assembly. The top track shows the contigs that appear in the discovar, falcon, and supernova assemblies. The paired-end track shows read coverage of the Discovar paired-end library. The mate-pair and Dovetail tracks show physical/fragment coverage of the mate-pair and Dovetail libraries, respectively. The bottom track shows GC content of the sequence as well as homopolymers sequences of at least 5  bp where A, C, G, and T are colored red, blue, yellow, and green, respectively.

To illustrate the performance of the different technologies sequencing different genomic features, we mapped whole-genome reads and assemblies to single BACs as shown in Fig. 5. None of our three whole-genome assemblies are able to reconstruct BAC 22; each breaking at a large (more than 12  kbp) repeat. The Discovar library (paired-end), mate-pair library, and Dovetail library were each mapped, and only reads mapping to a high quality and exhibiting up to one mismatch are shown in the figure. The mapping reveals several areas of high repetition, e.g., the arms and middle of a retrotransposon, and there are areas lacking coverage completely, which suggests a sequence that is difficult for our Illumina sequence data to resolve. We also see drops in coverage at some sites with high concentrations of homopolymers, as marked by colored lines in the GC content, e.g., an A rich region of ∼7  kbp. Interestingly, the repeat arms are also rich in homopolymers.

We note that the discovar-mp-dt-bn assembly leaves the largest gap around the repeat. The falcon assembly was able to completely cover an area with no mapping paired-end Illumina reads, which explains some of extra *k*-mer content in Fig. 2 noted earlier in this assembly. The supernova-bn assembly was able to reconstruct more of the difficult region, but it also contains duplications in the homopolymer-rich flanking regions that is not seen in the other assemblies.

The mate-pair library was not able to scaffold the discovar contigs due to the size of this repeat being larger than its 10  kbp insert size. The mate-pair fragments also map to a great depth in the repeat. Dovetail data, however, shows a much smoother fragment distribution and was able to scaffold the two discovar contigs in the correct order and orientation as it could scaffold up to 50  kbp (the cutoff used by the HiRise scaffolder). However, the gap length was not estimated with Dovetail and was arbitrarily set to 100 Ns when in reality the gap is over 12,000  bp long. While BioNano software estimates gap sizes, we note that BioNano data were not able to close this particular gap in any of the assemblies.

### Long-range accuracy using synteny to *S. tuberosum*

**Figure 6:**
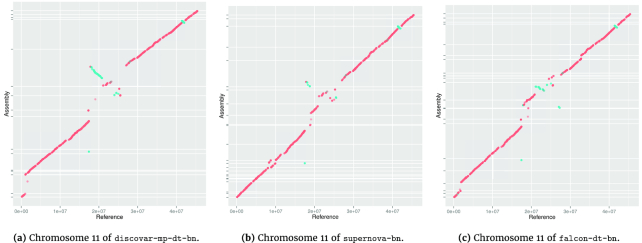
Mummer plots showing alignment to chromosome 11 of the *S. tuberosum* reference version 4.03. The *S. tuberosum* reference is shown on the *x*-axis and assembly scaffolds on the *y*-axis. Alignments shown are at least 10 kbp long and 90% identical.

As all our assemblies are *de novo*, in the sense that we used no prior information from other Solanaceae genomes, we reasoned that more accurate long-range scaffolding would be apparent as longer syntenic blocks to a closely related species. We used nucmer [47] to analyze the synteny of our assemblies to the pseudomolecules of the *S. tuberosum* genome [48]. Figure 6 shows the mummer plot for chromosome 11 of *S. tuberosum* against our three assemblies. We saw the falcon-dt-bn assembly showed the best synteny, with the discovar-mp-dt-bn being the worst. The plots for the remaining chromosomes are shown in Supplementary Figures S3.2, S3.3, and S3.4.

Using synteny, we identified two cases of chimerism, i.e., scaffolds that align well to two different pseudomolecules of the*S. tuberosum* genome. Both cases are in discovar-mp-dt-bn but not falcon-dt-bn. The first 1.5  Mbp of scaffold ScEqE3Q_528 maps to pseudomolecule 7, while the last 2.9  Mbp map to pseudomolecule 2 in the *S. tuberosum* genome. There is no conflict reported with the BioNano Genomics optical map in this area, but we can exclude the possibility that these are real chromosome structural arrangements in *S. verruscosum* because we have GbS markers on each end of this scaffold that also map in an *S. verrucosum* cross to these different linkage groups (López-Girona, unpublished work). The other case is a scaffold ScEqE3Q_633 in which the first 1.4  Mbp map to pseudomolecule 8 and the remainder to pseudomolecule 3. Here, BioNano Genomics does report a conflict that would highlight this error, and *S. verrucosum* genetic markers also support the chimera classification.

## Discussion

The quality and quantity of DNA available, whether it is from fresh or frozen tissue, and ease of its extraction will often dictate which preparation and sequencing technologies are feasible to use. Budget constraints do play a large part in the choice of technologies to be adopted for any genome project. Assembly and scaffolding methods are often effectively the choice of sequencing method, but the properties of the genome will also affect the results. Interestingly, none of the assembly approaches we used led to a “bad assembly,” e.g., one that fails to assemble large parts of the genome or makes many systematic errors (as seen in many early short-read assemblies). This speaks to the tremendous progress made in improved sequencing technologies and assembly algorithms. Instead, they differ mostly in the length of the ungapped sequence and scaffolds, with much smaller differences in missing sequence and gene content, duplicated regions, and per base accuracy.

A Discovar assembly is the cheapest and easiest to construct, and the resulting assembly is very accurate, albeit highly fragmented. Adding a long mate-pair library is a proven method of increasing the contiguity of a short-read assembly by scaffolding. The 10x Genomics-based assembly using Supernova was as easy to obtain as the Discovar assembly. The two most remarkable features of this assembly are the low cost and input DNA requirement; for only slightly higher cost than a Discovar assembly, and considerably less than with only one long mate-pair library, we obtained an assembly comparable to what one would expect from multiple long mate-pair libraries.

Our PacBio assembly using Falcon achieved contiguity similar to that of discovar-mp (Discovar plus long mate-pair scaffolding). PacBio sequencing has a considerably higher cost and material requirement than Illumina sequencing, but the falcon assembly contains truly contiguous sequence as opposed to discovar-mp, which contains gaps patched with Ns. The PacBio read lengths (N50 = 13.5  kbp) were similar to the insert size of mp library (mean 10  kb), and the read coverage was higher for PacBio (50×) than for the mp data (15×), but PacBio contigs (N50 = 712  kbp) are slightly shorter than the discovar-mp scaffolds (N50 = 858  kbp).

The addition of Dovetail showed the most striking increase in contiguity by scaffolding. We note that our Dovetail scaffolds provided the order and orientation of the constituent contigs but no estimate for the length of the gaps between them. This should be taken into consideration if true physical length of sequences is important and for specific downstream uses. Both Illumina (Discovar+mp) and PacBio (Falcon) assemblies are amenable to the addition of Dovetail, but the scaffolds produced from the Falcon contigs (4× increase) were not as long as those from the Illumina assembly (5.5× increase). This could be because while the Falcon assembly has been polished with PacBio reads, it retains some PacBio errors and so some Dovetail (Illumina) reads do not pass stringent mapping filters. If true, Pilon polishing with Illumina reads could help, as it improved the *k*-mer spectra (Fig. 2).

With BioNano Genomics restriction enzyme digest based optical maps, we obtained less (∼2× increase) scaffolding improvement than with Dovetail (4× to 5.5× increase). This could be due to three issues: first, that assembly gaps are not correctly sized, which prevents real and *in silico* restriction maps matching (as information is purely encoded in the distances between sites). We see that the ungapped PacBio assemblies improve more than scaffolded Illumina, and Dovetail scaffolds (with arbitrary 100  bp gaps) hardly increase at all. Second, because the method produces low information density (one enzyme site per ∼12  kbp), long fragments with many sites are needed to create significant matches, and our DNA was not sufficiently long (best run N50 was 240  kbp). Longer DNA (over 300  kbp) and perhaps multiple enzyme maps with iterative scaffolding could have improved the results. Third, we observe that the *in silico* restriction rates for Illumina and PacBio assemblies are similar (8.1 to 8.4 sites /100  kbp), whereas the actual observed rates from the physical map are much lower at 6.8 sites/100  kbp, suggesting that there could be a fraction of the genome missing from our assemblies, which is very low in sites such as centromeric or telomeric regions where the BioNano Genomics map cannot scaffold through.

Gapfilling using PBJelly offers an attractive method of using the long-read data from PacBio to improve an existing Illumina-based assembly. This closed many of the gaps in the scaffolds thereby decreasing the fraction of unknown sequence (Ns) and also increasing the contiguity. The increase in contiguity of the 10x Genomics assembly was the highest. It will be intriguing to see if an assembly approach combining Chromium data with long reads (directly on the assembly graph) can combine the best attributes of both data types to resolve complex regions.

Analysis of the *k*-mer content of the supernova, discovar, and falcon assemblies showed that the *k*-mer spectra of each assembly is very clean. We see slightly higher level of sequence duplication in the supernova assembly and, to a lesser extent, in the falcon assembly. All three assembly algorithms are diploid aware, meaning they are able to preserve both haplotypes. The gene content of each assembly was very similar, with all three of our long assemblies showing a high percentage of the expected genes. The 10x Genomics-based assembly showed a slightly lower count in both of our assessments, but the difference is very small.

We used multiple BAC assemblies of ∼100  kb insert size to illustrate the technical limitations of each method. Short-read methods cannot resolve many areas of repetition within a whole-genome sequencing assembly. This is especially noticeable in a plant genome with higher repeat content and is one of the major reasons for breaks in contiguity in these assemblies. In our example in Fig. 5, the long mate-pair library alone is not sufficient. It takes the larger fragment lengths within the Dovetail Chicago library to finally make the join in the whole-genome assembly.

Long-read technologies do not suffer as much with repeats and, in the case of PacBio, tend to have more random rather than systematic errors [49]. We can see in our examplar that the falcon assembly covers some of the repetitive region. The underlying BAC assembly was also obtained with PacBio and gave us a single true contig for the entire BAC. On close inspection, we noticed that difficult region was spanned by reads of length 22  kbp to 26  kbp. This shows that long reads are certainly able to span such regions of difficulty and to assemble them.

Recently ultra-long reads with an N50 of 99.7  kbp (max. 882  kbp) with ∼ 92% accuracy have been produced with the new MinION R9.4 chemistry using high-molecular-weight DNA from a human sample [12]. If this is also achievable on plant material, the remaining (mostly repetitive) fraction of genomes should become visible. The recent *Solanum penellii* Nanopore assembly [50] reported average read length 12.7  kbp and error rate of 18% to 20%.

To evaluate the longer-range accuracy of our genome assemblies, we compared them to the closely related *S. tuberosum* pseudomolecule assembly, which revealed good synteny with all three of our longest assemblies (discovar-mp-dt-bn, falcon-dt-bn, and supernova). There are some disagreements, especially in the centromeric areas, but as these appeared in all assemblies, they could illustrate real structural variation. We detected two chimeric scaffolds in the discovar-mp-dt-bn assembly, but neither is present in the falcon-dt-bn. The two Dovetail scaffolding processes shared the same Hi-C sequence data but were conducted many months apart (discovar-mp first and later falcon) and used different versions of Dovetail’s proprietary HiRise software, versions 0.9.6 and 1.3.0, respectively, which might have affected the results. On detailed examination, we see that the ScEqE3Q_528 scaffold chimeric join is made by Dovetail hopping through a fragmented area of short (1  kbp to 2  kbp) contigs. Such small contigs do not exist in the Falcon assembly, which may be why we do not find chimeras. BioNano Genomics finds it hard to map to areas with many Dovetail gaps (as these are set to an arbitrary 100  bp size), and this region also has a high enzyme nicking rate (nearly twice the genome average), including two areas where nicks are less than 200  bp apart and so would be optically merged. In scaffold ScEqE3Q_633, we detect that discovar-mp scaffold123 was correctly split by Dovetail data as chimeric (also highlighted by BioNano Genomics and genetic markers), but the scaffold was not broken at the exact chimeric join, and the remaining sequence from the wrong chromosome was sufficient for Dovetail to propagate the error. While we did not detect a high level of systematic errors in any of our assembly methods, the importance of using BioNano Genomics and genetic markers to identify chimeras that then can be broken is apparent.

Even though we found some surprisingly small differences between assemblies of *S. verrucosum*, this is an inbred diploid potato species with a medium-size genome and is in no way exceptional. As there are about 300,000 angiosperms alone [51], we remind the reader that many factors, e.g., genome size, the ease of high-quality high-molecular-weight DNA extraction, the types of repeat content, polyploidy or heterozygosity may pose additional hurdles affecting the choice of technology and how well they will perform. Heterozygosity, in particular, complicates the assembly process; if individual haplotypes are desired, this places limitations on which strategies can be used. The careful choice of sample where possible, such as a highly inbred plant or doubled haploid, can remove or minimize these problems. This approach was also adopted for the potato DM reference, whereby a completely homozygous “doubled monoploid” that was used as the heterozygous diploid RH genotype originally selected for sequencing proved difficult to assemble due to the extremely high level of heterozygosity. Newer methods have recently been developed to assemble diploid genomes into chromosome-scale phase blocks [52] or even to exploit the haplotype diversity using a “trio binning” approach developed by [53], so we expect to see more true diploid assemblies in the near future.

## Materials and Methods

### Project requirements

Each of the assembly methods we used comes with its own requirements. We have broken this down into material requirements, i.e., plant and DNA material, monetary requirements (the cost of preparation and sequencing), and computational requirements. Table 2 lists the material requirements for each library.

**Table 2:** Material requirements for each library

Library	Tissue type	Material/DNA amount	HMW	Fragment length (bp)
TALL	Frozen	3 μg	No	700
Discovar	Frozen	0.6 μg	No	500
Mate-pair	Frozen	4 μg	No	10,000
PacBio	Young frozen	5 g	No	20,000
BioNano	Young fresh	2.5 μg	Yes	>100,000
Dovetail	Fresh	20 g	Yes	>100,000
Chromium	Flash frozen	0.5 g	Yes	>100,000

Amounts in grams are for fresh/frozen material and amounts in micrograms for DNA. In each case where frozen or flash frozen is stated, fresh material is also acceptable.

We calculated costs taking into consideration the costs of consumables, laboratory time, and machine overheads, but not bioinformatics time. For sequencing costs, we used the Duke University cost as much as possible to provide comparative figures. Since several of the projects share common methods, such as sequencing a lane on a HiSeq 2500, we have broken down the costs into individual components. See Table 3 for our full costs calculations.

**Table 3:** The overall cost of each assembly project

Assembly	Paired-end	Mate-pair	PacBio	Chromium	Dovetail	BioNano	HiSeq 2500	MiSeq	PacBio RSII	Total (USD)
discovar	✗						✗			3,273
discovar-mp	✗	✗					✗	✗		7,854
discovar-mp-bn	✗	✗				✗	✗	✗		8,803
discovar-mp-dt	✗	✗			✗		✗✗	✗		32,793
discovar-mp-dt-bn	✗	✗			✗	✗	✗✗	✗		33,742
falcon			✗						✗	25,499
falcon-bn			✗			✗			✗	26,448
falcon-dt			✗		✗		✗		✗	50,438
falcon-dt-bn			✗		✗	✗	✗		✗	51,387
supernova				✗			✗			4,299
supernova-bn				✗		✗	✗			5,248
**Cost (USD)**	209	595	474	1,235*	21,875	949*	3,064	3,986	25,025	

We show which library preparations and sequencing runs are required for each assembly with a checkmark (✗). Individual costs are given at the bottom, and total costs of each assembly are on the right. All costs are according to Duke University as of April 2017 and in US dollars (USD), except those marked with an asterisk (*), which were according to the Earlham Institute and converted from Great British pounds (GBP) to US dollars at an exchange rate of 0.804 GBP/USD. Paired-end, mate-pair, PacBio, and Chromium are library preparations including DNA extraction. Dovetail includes Chicago library preparation and HiRise scaffolding. BioNano is the cost of building the optical map. HiSeq2500 is for a rapid run half flowcell (one lane) with 250  bp reads. MiSeq is for two runs with 300  bp reads. PacBio RSII is for 65 SMRT cells.

In many cases, the assemblies can be performed with modest scientific computing facilities. In some cases, notably for Supernova, a very large amount of memory is required. In this case, the computing requirement will not be available to most laboratories and will need to be sourced elsewhere. Table 4 shows the computational requirements of each assembly method.

**Table 4:** Computational requirements

Name of assembly	Approximate runtime	Peak memory (Gb)	Average memory (Gb)	System
Supernova	3 days	1 300		Large memory
Canu (Uncorr)	12 days	47	20	HPC cluster
Canu (Corr)	4 days	34	14	HPC cluster
Falcon	5 days	120	60	Large memory
HGAP	2 minutes	280	--	Large memory
Discovar	22 hours	260	134	Large memory
ABySS	1 week	64	--	HPC cluster
BioNano (Asm)	8 hours	64	64	HPC cluster
BioNano (Scaf)	1 day	64	64	HPC cluster

### Library preparation and sequencing

In this section, we briefly describe methods for library preparation and sequencing. For a comprehensive description, please see the Supplementary Material.

*Solanum verrucosum* accesssion Ver-54 was grown in the glass house in James Hutton Institute in Scotland. Both fresh and frozen leaves from this accession and its clones were used for DNA extraction.

The TALL library was prepared using 3 μg of DNA, and fragments of 650  bp were sequenced with a HiSeq2500 with a 2×150  bp read metric. The Discovar library was prepared using 600  ng of DNA, and fragments of 500  bp were sequenced with a HiSeq2500 with a 2×250  bp read metric.

The mate-pair library was prepared using 4  μg of DNA, and fragments of 10  kbp were circularized, fragmented, and sequenced on a MiSeq with a 2×300, bp read metric [54].

A PacBio library was prepared using 5, g of frozen leaf material. A 20 ,kbp fragment length library was prepared according to manufacturer’s instructions and sequenced on 65 SMRT cells with the P6C4 chemistry on a PacBio RSII.

The 10x Chromium library was prepared according to the manufacturer’s instructions and sequenced on a HiSeq2500 with a 2×250, bp read metric.

For BioNano, DNA was extracted using the IrysPrep protocol. A total of 300  ng was used in the Nick, Label, Repair, and Stain reaction and loaded onto a single flow cell on a BioNano chip. The chip was run eight times to generate 252, Gb of raw data.

### Assembly and evaluation

All tools and scripts that were used to perform the evaluation and produce the figures are available on GitHub in the georgek/potato-figures repository.

We used Rampart (Rampart, RRID:SCR_016742) [55] to run ABySS [33] multiple times with different *k* values. Discovar*de novo* was run with normal parameters.

Long mate-pair reads were first processed with NextClip (NextClip, RRID:SCR_005465) [56] to remove the Nextera adapter. Soapdenovo2 was then used to perform scaffolding with both the paired-end and mate-pair libraries.

*k*-mer content was analyzed with the kat comp tool [43] (KAT, RRID:SCR_016741). We used default parameters with manually adjusted plot axes to show the relevant information.

We used the Busco core plant dataset to evaluate the gene content. The *S. tuberosum* representative transcripts (PGSC_DM_V403_representative_genes from http://solanaceae.plantbiology.msu.edu/pgsc_download.shtml) were aligned to the assemblies using Blast and the coverage of transcripts at various thresholds using a tool we developed.

The BACs were sequenced with the Earlham Institute BAC pipeline [57] and were assembled with Discovar*de novo* using normal parameters after filtering for *Escherichia coli* and the BAC vector. The PacBio BAC was assembled using HGAP3 [36]. We used GNU parallel [58] for concurrent assembly and analysis.

A total of 20 BACs that assembled into a single contig were selected to use as a reference. These BACs are non-redundant to the extent that they did not share any lengths of sequence of more than 95% identity and over 5,000  bp long. Short reads were aligned to the BACs using Bowtie2 [59] with default parameters. The assemblies were mapped to the BACs using bwa mem [60]. The mapped sequences were sorted and filtered for quality using sambamba [61]. Fragment coverage was calculated using samtools [62] and bedtools [63].

Synteny was analyzed with mummer [64]. We used nucmer to align the assemblies to the *S. tuberosum* reference v4.04 [65]. Alignments less than 10  kbp and 90% identity were filtered out.

## Supplementary Material

GIGA-D-18-00164_Oroginal_Submission.pdf

GIGA-D-18-00164_Revision_1.pdf

GIGA-D-18-00164_Revision_2.pdf

Response_to_Reviewer_Comments_Original_Submission.pdf

Response_to_Reviewer_Comments_Revision_1.pdf

Reviewer_1_Report_Orginal_Submission -- CÃ©cile Monat 6/13/2018 Reviewed

Reviewer_2_Report_Orginal_Submission -- Alex Harkess 6/19/2018 Reviewed

Reviewer_3_Report_Orginal_Submission -- Sachiko Isobe 6/28/2018 Reviewed

Supplemental File

## Data Availability

All read data generated in this study have been submitted to the EMBL-EBI European Nucleotide Archive under project PRJEB20860. Archival copy of the code, assemblies, and other data are available in the *GigaScience* GigaDB repository [66].

## Abbreviations

BAC: Bacterial Artificial Chromosome; BLAST: Basic Local Alignment Search Tool; BUSCO: Benchmarking Universal Single-Copy Orthologs; LMP: long mate-pair; PacBio: Pacific Biosciences; SMRT: single-molecule real-time; TALL: Tight and Long Library.
