# Supplementary material for: A critical comparison of technologies for a plant genome sequencing project
Source: Gigascience. 2019 Jan 9;8(3):giy163. doi: 10.1093/gigascience/giy163 (PMC6423373; doi:10.1093/gigascience/giy163)
Supplement: GIGA-D-18-00164_Revision_2.pdf [file giy163_giga-d-18-00164_revision_2.pdf]

GigaScience

A critical comparison of technologies for a plant genome sequencing project

--Manuscript Draft--

Manuscript Number:	GIGA-D-18-00164R2	
Full Title:	A critical comparison of technologies for a plant<br>genome sequencing project	
Article Type:	Research	
Funding Information:	Biotechnology and Biological Sciences Research Council<br>(BB/K019325/1)	Dr Glenn Bryan
	Biotechnology and Biological Sciences Research Council<br>(BB/K019090/1)	Dr Matthew D Clark
Abstract:	A high quality genome sequence of any model organism is an essential starting point for genetic and other studies. Older<br>clone based methods are slow and expensive, whereas faster, cheaper short read only assemblies can be incomplete and<br>highly fragmented, which minimises their usefulness. The last few years have seen the introduction of many new<br>technologies for genome assembly. These new technologies and associated new algorithms are typically benchmarked on<br>microbial genomes or, if they scale appropriately, on larger (e.g. human) genomes. However, plant genomes can be much<br>more repetitive and larger than the human genome, and plant biochemistry often makes obtaining high quality DNA free<br>from contaminants dicult. Reecting their challenging nature we observe that plant genome assembly statistics are<br>typically poorer than for vertebrates. Here we compare Illumina short read, PacBio long read, 10x Genomics linked reads,<br>Dovetail Hi-C and BioNano Genomics optical maps, singly and combined, in producing high quality long range genome<br>assemblies of the potato species S. verrucosum. We benchmark the assemblies for completeness and accuracy, as well as<br>DNA, compute requirements and sequencing costs. We expect that our results will be helpful to other genome projects, and<br>that these datasets will be used in benchmarking by assembly algorithm developers.	
Corresponding Author:	Matthew D Clark, Ph.D.<br>Natural History Museum<br>London, London UNITED KINGDOM	
Corresponding Author Secondary Information:		
Corresponding Author's Institution:	Natural History Museum	
Corresponding Author's Secondary Institution:		
First Author:	Pirita Paajanen, Ph.D.	
First Author Secondary Information:		
Order of Authors:	Pirita Paajanen, Ph.D.	
	George Kettleborough, Ph.D.	
	Elena López-Girona, Ph.D.	
	Michael Giolai, M.Sc.	
	Darren Heavens, B.Sc.	

Powered by Editorial Manager® and ProduXion Manager® from Aries Systems Corporation

	David Baker, B.Sc.
	Fiorella Cugliandolo
	Ashleigh Lister
	Gail Wilde
	Ingo Hein, Ph.D.
	Iain Macaulay, Ph.D.
	Glenn Bryan, Ph.D.
	Matthew D Clark, Ph.D.
**Order of Authors Secondary Information:**	
**Response to Reviewers:**	Dear Scott,<br>we have made the minor revisions you requested (pasted below as an aide memoire). My postdoc is applying for her first lecturer position at the moment so is keen to have this in print or online soon if thats possible.<br><br>Hope this finds you well and have a great christmas. Maybe see you at AGBT?<br><br>Matt<br><br>###########<br>We are now happy with your manuscript "A critical comparison of technologies for a plant genome sequencing project" (GIGA-D-18-00164R1). I am pleased to inform you that it is potentially acceptable for publication in GigaScience, once you have carried out some essential revisions to make it meet our reproducibility policies.<br><br>1. Can you change the Data Access section to Availability of Supporting Data and add an additional line to cite the GigaDB. This can be something similar to this:<br><br>Archival copy of the code, assemblies and other data are available in the GigaScience GigaDB repository[66].<br><br>Then cite the Data DOI in the references as reference 66:<br><br>Paajanen P; Kettleborough G; López-Girona E; Giolai M; Heavens D; Baker D; Lister A; Cugliandolo F; Wilde G; Hein I; Macaulay I; Bryan GJ; Clark MD (2018): Supporting data for "A critical comparison of technologies for a plant genome sequencing project" GigaScience Database. http://dx.doi.org/10.5524/100518<br><br>2. Can you add all relevant accessions and identifiers such as ORCID IDs (for authors that have them), accessions and RRIDs for the software (see: ftp://penguin.genomics.cn/pub/10.5524/RRID/RRIDlist.pdf), as well as version numbers for the software.<br><br>Once you have made the necessary corrections, please submit a revised manuscript online
**Additional Information:**	
**Question**	**Response**
Are you submitting this manuscript to a special series or article collection?	No
**Experimental design and statistics**<br><br><br>Full details of the experimental design and statistical methods used should be given in the Methods section, as detailed in our	Yes

Minimum Standards Reporting Checklist. Information essential to interpreting the data presented should be made available in the figure legends.<br><br>Have you included all the information requested in your manuscript?	
**Resources**<br><br>A description of all resources used, including antibodies, cell lines, animals and software tools, with enough information to allow them to be uniquely identified, should be included in the Methods section. Authors are strongly encouraged to cite Research Resource Identifiers (RRIDs) for antibodies, model organisms and tools, where possible.<br><br>Have you included the information requested as detailed in our Minimum Standards Reporting Checklist?	Yes
**Availability of data and materials**<br><br>All datasets and code on which the conclusions of the paper rely must be either included in your submission or deposited in publicly available repositories (where available and ethically appropriate), referencing such data using a unique identifier in the references and in the "Availability of Data and Materials" section of your manuscript.<br><br>Have you have met the above requirement as detailed in our Minimum Standards Reporting Checklist?	Yes

Manuscript
Click here to access/download;Manuscript;main.pdf
Click here to view linked References

OXFORD
(GIGA)^n SCIENCE
GigaScience, 2018, 1–12
doi: xx.xxxx/xxxx
Manuscript in Preparation
Paper

PAPER

A critical comparison of technologies for a plant genome sequencing project

Pirita Paajanen[1,3,†], George Kettleborough[1,†], Elena López-Girona[2,4], Michael Giolai[1], Darren Heavens[1], David Baker[1], Ashleigh Lister[1], Fiorella Cugliandolo[1], Gail Wilde[2], Ingo Hein[2], Iain Macaulay[1], Glenn J. Bryan[2] and and Matthew D. Clark[1,5,*]

[1]Earlham Institute, Norwich, UK and [2]The James Hutton Institute, Invergowrie, Dundee, UK and [3]Department of Cell and Developmental Biology, John Innes Centre, Norwich, UK and [4]The New Zealand Institute for Plant & Food Research Limited, Palmerston North, New Zealand and [5]Department of Life Sciences, Natural History Museum, London, UK

[*]matt.clark@nhm.ac.uk
[†]Contributed equally.

Abstract

Background A high quality genome sequence of any model organism is an essential starting point for genetic and other studies. Older clone based methods are slow and expensive, whereas faster, cheaper short read only assemblies can be incomplete and highly fragmented, which minimises their usefulness. The last few years have seen the introduction of many new technologies for genome assembly. These new technologies and associated new algorithms are typically benchmarked on microbial genomes or, if they scale appropriately, on larger (e.g. human) genomes. However, plant genomes can be much more repetitive and larger than the human genome, and plant biochemistry often makes obtaining high quality DNA free from contaminants difficult. Reflecting their challenging nature we observe that plant genome assembly statistics are typically poorer than for vertebrates.
Results Here we compare Illumina short read, PacBio long read, 10x Genomics linked reads, Dovetail Hi-C and BioNano Genomics optical maps, singly and combined, in producing high quality long range genome assemblies of the potato species *S. verrucosum*. We benchmark the assemblies for completeness and accuracy, as well as DNA, compute requirements and sequencing costs.
Conclusions The field of genome sequencing and assembly is reaching maturity and the differences we observe between assemblies are surprisingly small. We expect that our results will be helpful to other genome projects, and that these datasets will be used in benchmarking by assembly algorithm developers.

Key words: assembly, long reads, short reads, optical mapping, Pacific Biosciences, PacBio, 10x Genomics.

Developments in high-throughput sequencing have revolutionised genetics and genomics, with lower costs leading to an explosion in genome sequencing project size [1] and number of species [2]. Genomes from many diverse organisms have been sequenced, from marsupials to microbes, plants, phytoplankton, and fungi, amongst many others [3]. For a while it has been feasible for a single lab to sequence and *de novo* assemble a complex genome (for example, [4]).

The existence of very high quality references [5, 6] has made the human genome popular for demonstrating new sequenc-

Compiled on: 11th December 2018.
Draft manuscript prepared by the author.

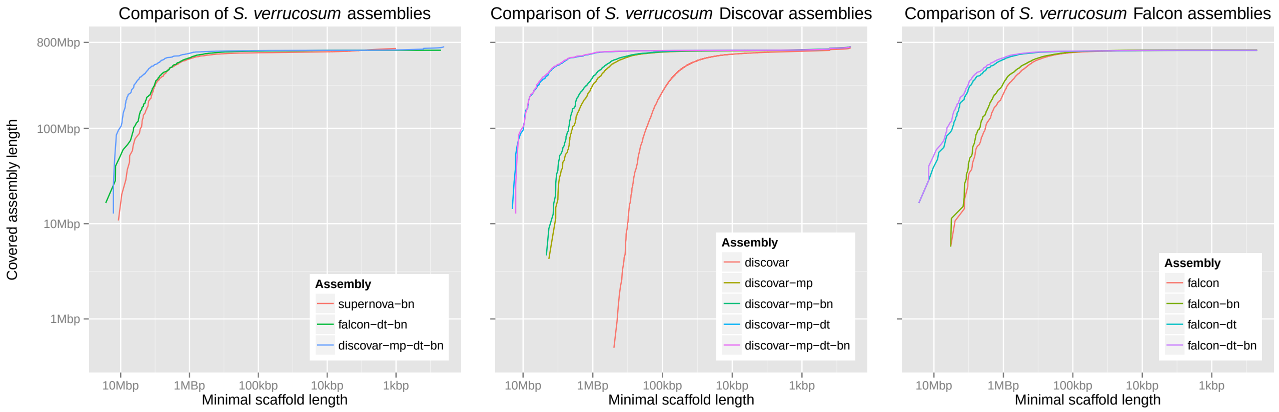

Figure 1. Comparison of contig/scaffold lengths and total assembly sizes of the various *S. verrucosum* assemblies.

ing technologies and assembly algorithms. The human genome has now been sequenced and assembled using various technologies including Sanger, 454, IonTorrent, Illumina, Pacific Biosciences (PacBio), 10x Genomics and even nanopore sequencing technologies [7, 8, 9, 10, 11, 12]. Hybrid approaches have also been used which combine complementary technologies, for example PacBio and BioNano [13].

However, the human genome is not representative of all eukaryotic genomes; plant genomes in particular are typically more repetitive (including multi-kilobase long retrotransposon elements as well as even longer regions comprising of "nested" transposon insertions). Plant biology also poses challenges for the isolation of high quality high molecular weight DNA, due to strong cell walls, co-purifying polysaccharides, and secondary metabolites which inhibit enzymes or directly damage DNA [14]. Thus technologies that work well on vertebrate genomes may not work well for plants [15]. For these reasons slow and expensive clone based minimal tiling path sequencing approaches have persisted in plants [16, 17] long after faster, cheaper short read whole genome assemblies were first demonstrated for vertebrate genomes [18]. In addition to increased genome repetitiveness and size, polyploidy is common in plants (especially key crops such as cotton, brassicas, wheat, potatoes) as are high levels of heterozygosity, especially where inbreeding is problematic due to generation times [19] or the plants are obligate outcrossers.

Plant biology poses some additional challenges for the isolation of high quality high molecular weight DNA. Plant cells possess strong rigid cell walls not broken by the addition of a detergent and, when physically breaking the cell wall, the DNA can be sheared, rendering the isolation of high molecular weight DNA problematic. A large proportion of the DNA in a plant cell can be from organelles (mitochondrial and chloroplast) [20] which are high copy number and large, for example the mitochondial genome is 453 kbp in wheat [21] but only 16 kbp in human [22]. Plants are also rich in polysaccharides which can co-purify with DNA, and they produce secondary metabolites to protect themselves from herbivores [14].

Plant genomes also vary hugely in size, from 61 Mbp (*Genlisea tuberosa*, a member of the bladderwort family [23]) to 150 Gbp (*Paris japonica*, a relative of lilies [24]), it is still nontrivial to design a *de novo* assembly project which involves an ensemble of technologies. Each platform comes with its own input requirements, computational requirements, quality of output and, of course, labour and materials costs. Our results can be used as guidance for further sequencing assembly projects and provide a basis for comparative genome studies, as each sequencing strategy and assembly method has its own biases.

In this paper we compare several practical *de novo* assembly projects of a Mexican wild potato species *Solanum verrucosum*. We chose this genome because *Solanum verrucosum* is a self-compatible, diploid, tuber-bearing, wild potato species which we inbred further to produce the line Ver-54. The estimated genome size based on *k*-mer content is 722 Mbp. In addition, recent cytogenetic and molecular studies have shown it likely represents a genome donor to Mexican allopolyploid potatoes [25, 26] and as such is taxonomically distant from the genetically characterised cultivars and landraces, although it has been classified into the same larger phylogenetic potato clade (Clade 4) as cultivated potatoes [27]. The Mexican allopolyploids in Series Longipedicellata and Demissa have very high levels of resistance to *Phytophthora infestans* (encoded by several R-genes) as does *S. verrucosum*. Thus, the *S. verrucosum* genome can be a highly useful genetic resource and a "potato model" for forward/reverse genetic studies relating to its high level of blight resistance, its unusually high level of self-fertility, and because it produces tubers, albeit small inedible ones. The Solanaceae, or nightshades, are a family containing many economically important, and previously sequenced, plants including potato *S. tuberosum* [28], tomato *S. lycopersicum* [29], aubergine *S. melongena* [30], and pepper *Capsicum annuum* [31]. These related species genomes can provide information about genome organisation in the Solanaceae, and allow comparative genomic studies.

Results

The results of this study are presented in two parts. In the first part we compare several short read (Illumina) to long read (PacBio) based assemblies. These represent the simplest type of sequencing projects that are often undertaken. We then choose one each of the Illumina based and one PacBio based assembly and in the second part we will use various different combinations of longer-range scaffolding data from newer technologies, namely *in vitro* Hi-C (Dovetail), optical mapping (BioNano Genomics) to increase continuity. Finally we compare these approaches to the read clouds (10x Genomics Chromium) technology, which promises short read assembly and longer-range scaffolding simultaneously. Validating the assemblies for sequence and scaffolding accuracy we find strengths and weaknesses, and that methods differ hugely in their DNA, time, computational requirements and cost.

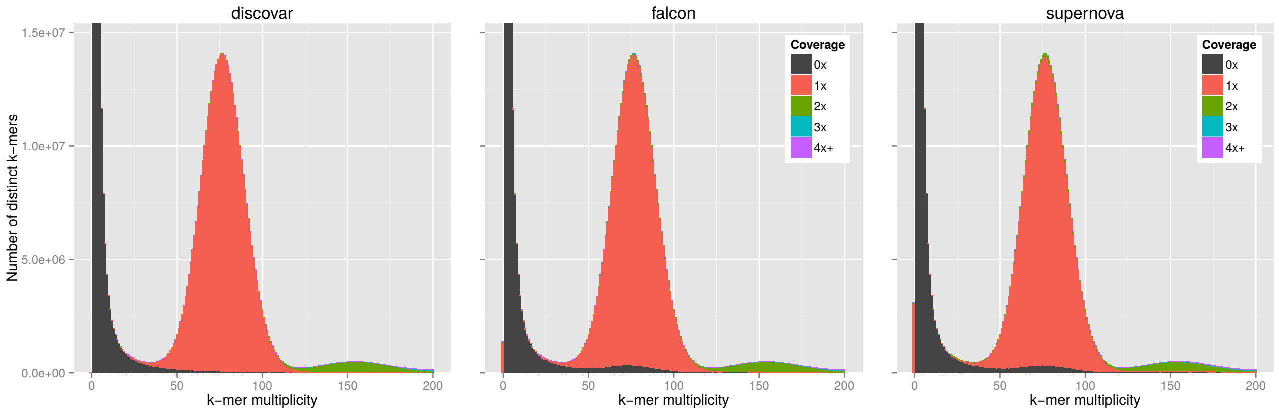

Figure 2. *k*-mer spectra plots from the *k*-mer Analysis Toolkit (KAT) comparing three *S. verrucosum* contig assemblies. The heights of the bars indicate how many *k*-mers of each multiplicity appear in the raw Discovar reads. The colours indicate how many times those *k*-mers appear in the respective assemblies with black being zero times and red being one time. A coloured bar at zero multiplicity indicates *k*-mers appearing in the assembly which do not appear in the reads. The Falcon assembly has been polished with the Illumina reads using Pilon to reduce the effect of using a different sequencing platform.

Contig assembly and scaffolding

The first stage of an assembly is to piece together reads to form long contiguous sequences, or *contigs* for short. These contigs can be ordered and oriented using longer-range information such as jumping/mate pair libraries. Throughout this paper we will refer to different contig assemblies that have been scaffolded. We use a naming convention which shows all of the steps used to construct the assembly. Each assembly name contains the steps used in order, separated by a hyphen. For example, the `discovar-mp-dt-bn` assembly is the `discovar` contig assembly scaffolded first with mate-pairs, then Dovetail and finally BioNano.

Illumina contig assembly

Two libraries were constructed for Illumina assembly. The first is a PCR-free library with insert size 500 bp (±40 %) which was sequenced with 250 bp paired-end reads on a single Illumina HiSeq 2500 run. We refer to this below as the Discovar library. The coverage of the library was 120×. The second library is a PCR-free "Tight and Long Library" (TALL) with insert size 650 bp (±20 %) sequenced with 100 bp and 150 bp paired-end reads on two Illumina HiSeq 2500 runs. The coverage of this library was 135×.

We analysed the TALL library reads with preqc, part of the SGA assembler [32], and giving a genome size estimate at 702 Mbp, while the same analysis on the DISCOVAR library yielded 722 Mbp. The latter agrees better with the 727 Mbp size of the potato genome assembly [28].

Assembly	Number of contigs	N50 (kbp)	Max length (kbp)	Total length (Mbp)
`abyss`	33 146	75	642	702
`abyss-mp`	21 376	331	2 288	712
`discovar`	25 216	77	498	646
`discovar-mp`	8 074	858	4 266	665
`hgap`	5 446	585	4 876	716
`canu`	8 138	290	4 701	722
`falcon`	2 442	712	5 738	659

Table 1. Assembly statistics of Illumina and PacBio assemblies, with a minimum contig/scaffold size of 1 kbp. `abyss` uses the TALL library, `discovar` uses the Discovar library, and `hgap`, `canu` and `falcon` use the PacBio library. For a more comprehensive summary, see Supplementary Table S3.1.

The TALL library was assembled with ABySS (ABySS, RRID:SCR_010709)[33] (*k*-mer size 113) and the Discovar library using Discovar *de novo* (Discovar, RRID:SCR_016755) [34] producing contig assemblies `discovar` and `abyss`, respectively.

The results for these two Illumina assemblies are similar in contiguity and shown in Table 1. However, while ABySS assembled about 8% longer total length, the number of small contigs was larger leading to very similar contig N50 to Discovar. One additional feature was that AbySS performed more scaffolding using the paired end data but did not fill many of the introduced gaps leading to about 100 times higher percentage of N bases than Discovar. These assemblies are more contiguous than the equivalent contig assemblies of the *S. tuberosum* genome where the reported contig N50 from paired-end reads is 22.4 kbp [28].

Illumina scaffolding

A Nextera long mate-pair (LMP) library was made with insert size 10 000 bp (±20 %) and sequenced on two lanes of an Illumina MiSeq with fragment size 500 bp and 300 bp reads. The total coverage of the LMP library was 15× after we had filtered out duplicates 23.4%*ofreads*, reads that did not contain a Nextera adapter or were too short to be useful.

We scaffolded both the `discovar` and `abyss` assemblies separately using Soapdenovo2 (soapdenovo2,RRID:SCR_014986) [35] producing `discovar-mp` and `abyss-mp`, respectively. The contiguity of both was increased significantly as shown in Table 1. Here the `discovar-mp` scaffolds were slightly better so we used this assembly to take forward for longer range scaffolding with other data types.

PacBio assembly

A gel size selected PacBio library with fragment lengths of at least 20 kbp was made according to the manufacturer's instructions. The library was sequenced using a PacBio RSII instrument and P6C4 chemistry. We sequenced 65 SMRT cells total, each giving about 500 MB of data and a total coverage of 50×. The N50 of the fragments was 13 499 bp and total number of reads 9 768 980.

We conducted three long read assemblies on the same data using HGAP3 [36], part of SMRT-analysis (version 2.3.0p5) (SMRT-Analysis, RRID:SCR_002942), Canu (Canu, RRID:SCR_015880) [37] (version 1.0), and Falcon (Falcon, RRID:SCR_016089) [38] (version 0.3.0) producing the `hgap`, `canu` and `falcon` assemblies, respectively. The assembly statistics for each is shown in Table 1. Another long read assembler, that we chose not to use, because it does not include any error

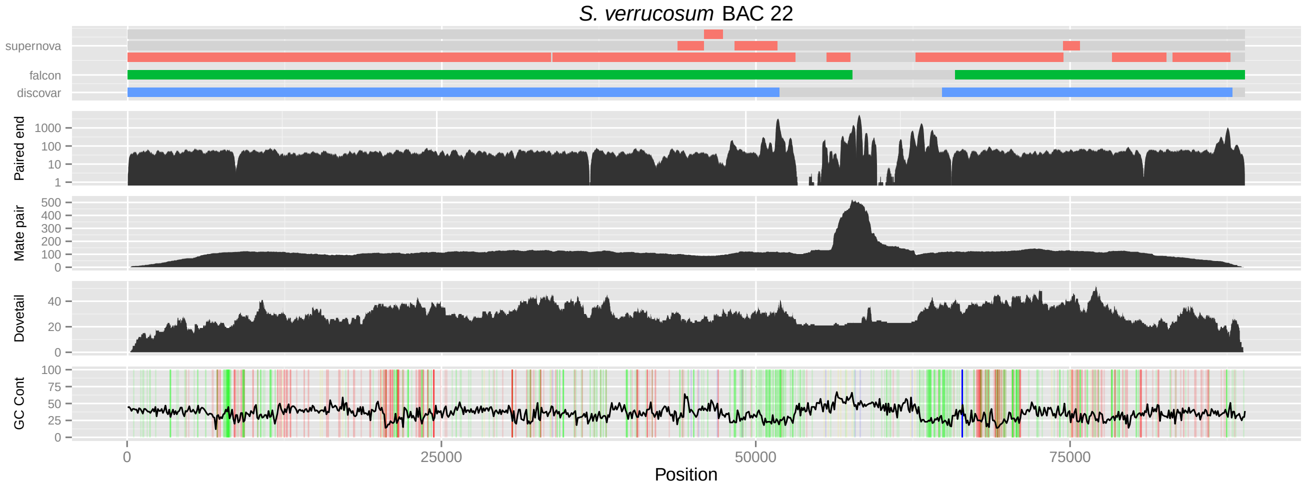

Figure 3. A difficult region of the genome which is contiguously assembled with a PacBio BAC but in none of our whole genome assemblies. The region was correctly scaffolded by Dovetail. The figure shows various alignments and information with respect to the BAC assembly. The top track shows the contigs which appear in the `discovar`, `falcon` and `supernova` assemblies. The paired-end track shows read coverage of the Discovar paired-end library. The mate-pair and Dovetail tracks show physical/fragment coverage of the mate-pair and Dovetail libraries, respectively. The bottom track shows GC content of the sequence as well as homopolymers sequences of at least 5 bp where A, C, G, and T are coloured as red, blue, yellow, and green, respectively.

correction is miniasm [39]. This is a fast lower computational power alternative to the ones that we used in this paper and is useful for many purposes e.g. empirical testing of long read assemblies.

The Canu assembly was made with reads that were first error-corrected by the HGAP3 pipeline because the first attempt using raw reads resulted in an excessive amounts of small scaffolds and a genome size more than 50 % longer than expected.

The `canu` and `hgap` assemblies contain slightly more sequence content (as measured by the total length of the assembly), and a lower percentage of unknown bases (as measured by the percentage of bases denoted by N) than the short read assemblies. This may be due to their capturing of additional difficult sequences, especially repeat elements which short read assemblies are known to have problems traversing. The `falcon` assembly has the highest N50, and while `canu` is closest to the estimated genome length. Falcon also produced 9.9 Mbp of alternate contigs, likely from residual heterozygosity, which will be useful for interpreting downstream genetic results e.g. forward and reverse genetic screens. We also found this assembly was easier and faster to run than HGAP3. We also found the basepair accuracy of canu read correction to be lower than HGAP3 read correction. For these reasons we chose the `falcon` assembly to take forward to hybrid scaffolding. We first polished it using Quiver as part of SMRT-analysis (version 2.3.0p5).

Longer-range scaffolding

To achieve higher contiguity, newer technologies have been developed to complement the previous methods and, in some cases, each other. In this section we investigate using longer range scaffolding methods to increase the contiguity of the Illumina `discovar-mp` assembly and the `falcon` PacBio assembly. We also investigate the 10x Genomics Chromium platform, an integrated solution which can be used to generate short Illumina reads with long-range positional information.

Dovetail

Dovetail Genomics provides a specialised library preparation method called Chicago and an assembly service using a custom scaffolder called HiRise. The Chicago library preparation technique is based on the Hi-C method, producing deliberately "chimeric" inserts linking DNA fragments from distant parts of the original molecule [40]. This is followed by standard Illumina paired-end sequencing of the inserts. Since the separation of the original fragments follows a well-modelled insert size distribution, the scaffolder is able to join contigs to form scaffolds spanning large distances, even up to 500 kbp [40].

Dovetail Genomics, LLC (Santa Cruz, CA, USA) received fresh leaf material from us from which they constructed a Chicago library. This was sequenced at Earlham Institute using Illumina 250 bp paired-end reads. The total read coverage of the Chicago library was 105×. Dovetail used their HiRise software to further scaffold the `discovar-mp` assembly, increasing the N50 from 858 kbp to 4713 kbp, and the `falcon` assembly, increasing the N50 from 712 kbp to 2553 kbp. These assemblies are called `discovar-mp-dt` and `falcon-dt`, respectively.

BioNano

The BioNano Genomics Irys platform constructs a physical map using very large DNA fragments digested at known sequence motifs with a specific nicking enzyme, to which a polymerase adds a fluorescent nucleotide. The molecules are scanned, and the distance between nicks generates a fingerprint of each molecule which is then used to build a whole genome physical map. Sequence-based scaffolds or contigs can be integrated by performing the same digestion *in silico* then ordering and orienting the contigs according to the physical map [41].

We collected BioNano data from 16 runs by repeatedly running the same chip. After filtering fragments less than 100 kbp, the yield varied from 0.8 Gb to 25.8 Gb, with the earlier runs yielding more whereas the molecule N50 was higher in later runs (ranging from 135 kbp to 240 kbp). The total yield of BioNano data was 252 Gbp which is roughly equivalent to 350× coverage.

We performed hybrid scaffolding on the `discovar-mp` and `falcon` assemblies. The *in silico* digest suggested a label density of 8.1/100 kbp for `discovar-mp` and 8.4/100 kbp for `falcon` whilst the actual observed density was only 6.8/100 kbp. We used the BioNano pipeline (v2.0) (BioNano Irys, RRID:SCR_016754) to scaffold `discovar-mp`, increasing the N50 from 858 kbp to 1260 kbp, and `falcon`, increasing the N50 from 710 kbp to 1500 kbp. These assemblies are called `discovar-mp-bn` and `falcon-bn`, respectively.

10x Genomics

10x Genomics provides an integrated microfluidics based platform for generating linked reads (a cloud of non-contiguous reads with the same barcode from the same original DNA molecule) and customised software for their analysis [11]. Large fragments of genomic DNA are combined with individually barcoded gel beads into micelles in which library fragments are constructed and then sequenced as a standard Illumina library. Using the barcodes the reads from the same gel bead can be grouped together.

Unlike the previous two longer-range scaffolding approaches, the 10x Genomics platform constructs a new paired-end library which can be sequenced and then assembled into large scaffolds by one assembly program: Supernova.

A 10x Genomics Chromium library was made according to manufacturer's instructions and a lane of Illumina HiSeq 250 bp paired-end reads were generated with a coverage of about 92×. Supernova (version 1.1.1) (Supernova, RRID:SCR_016756) produced the `supernova` assembly with length 641 Mbp and a scaffold N50 of 2.33 Mbp. Trimming reads back to 150 bp or reducing sequencing depth to 56×, which are the read length and depth recommended by 10x Genomics, generated very similar results (see Supplemental Section 2.3) compared to the ones reported above.

Hybrid scaffolding

It is possible to iteratively combine these longer-range scaffolding approaches. We tested several hybrid approaches using the `discovar`, `falcon` and `supernova` assemblies. For example the `discovar-mp` assembly was scaffolded using Dovetail and then BioNano producing `discovar-mp-dt-bn` with an N50 of 7.0 Mbp, the highest contiguity of any assembly reported here. The `falcon` assembly when scaffolded with both produced scaffolds with an N50 of only 3.09 Mbp, lower than with BioNano alone. Finally we scaffolded the `supernova` assembly with BioNano producing `supernova-bn` which increased the N50 from 2.33 Mbp to 2.85 Mbp.

Most scaffolding steps add gaps of unknown sequence, so we also used long reads from PacBio to scaffold and to perform "gapfilling" on the assemblies, replacing regions of unknown sequence (N stretches) with a PacBio consensus sequence. This also presents an opportunity to use lower coverage PacBio data to improve an Illumina assembly, which may be more cost effective than a *de novo* assembly using PacBio. PBJelly (version 15.2.20) [42] was used to perform gapfilling using only 10 SMRTcells of PacBio data (8× depth). The Supernova assembly increased in size from 641 Mbp to 671 Mbp, and N50 from 2.33 Mbp to 2.64 Mbp, and the amount of Ns present reduced from 7.58 % to 5.14 %. The `discovar-mp-dt` assembly increased in size from 656 Mbp to 680 Mbp and N50 from 4.69 Mbp to 4.87 Mbp, with Ns reduced from 3.03 % to 1.28 %. However, how gaps and percentage Ns are generated differs between assembly methods (see Discussion).

Assembly evaluation

Achieving a genome assembly with high levels of contiguity is potentially useless if it does not faithfully represent the original genome sequence. We assessed errors in assemblies by comparison to the raw data used to make the assemblies, as well as measuring gene content, local accuracy (BAC assemblies), and long-range synteny with the close relative *Solanum tuberosum*.

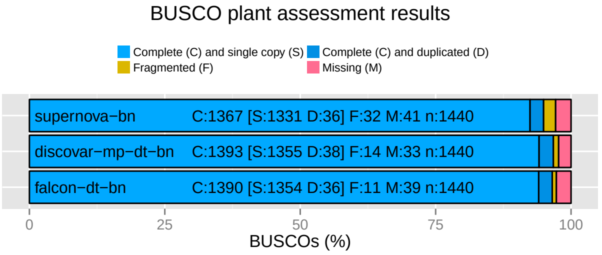

Figure 4. Busco analysis of `supernova-bn`, `discovar-mp-dt-bn`, and `falcon-dt-bn` using the plant gene dataset.

K-mer content

Analysis of the *k*-mer content of an assembly gives a broad overview of how well the assembly represents the underlying genome. We used the PCR-free Illumina Discovar library as our reference for the *k*-mer content of the genome. Due to the high accuracy of the reads we expect the *k*-mer spectra for a library to form a number of distributions which correspond to read errors, non-repetitive, and repetitive content in the genome. These distributions can be seen by observing only the shapes and ignoring the colours in Figure 2. The reader is referred to the KAT (KAT, RRID:SCR_016741) documentation for further details [43].

In Figure 2 we compare the *k*-mer contents of the three contig assemblies—`discovar`, `falcon`, and `supernova`—to the Discovar library. To minimise the effects of the differences between Illumina and PacBio sequencing error profiles the `falcon` assembly has been polished with the Illumina reads using Pilon [44] (see Supplemental Figure S3.1 for the unpolished plot).

The small red bar on the origin in some plots shows content which appears in the assembly but not in the Illumina reads. The `discovar` assembly is very faithful to the content in the library. The black area denotes sequences in the reads but not in the assembly: those clustering at the origin are predicted sequence errors in the reads, the small amount between 50 to 100 on the *x*-axis is sequence missing from the assembly. The dominant red peak (1×, around multiplicity 77), which is the vast majority of all assemblies here, contains content in the Illumina reads which appears once in the assembly (homozygous sample). Green areas on top of the main peak in Falcon and Supernova represents possible duplications in the assembly, whereas the green (2×) small peak to the right of the main peak is probably true duplicates— as these sequences are present twice in the assembly and at twice the expected read counts. At the main peak (*k*-mer multiplicity 77), the amount of potentially duplicated content in the assemblies (that is, number of *k*-mers appearing more than once in the assembly) is 0.66 % in `falcon`, 1.3 % in `supernova`, and 0.15 % in `discovar`.

Gene content

We assessed the gene content of the three most contiguous assemblies—`discovar-mp-dt-bn`, `falcon-dt-bn`, and `supernova-bn`—using two datasets. The first is with Busco and its `embryophyta_odb9` (plants) dataset (BUSCO, RRID:SCR_015008) [45] and the second is all the predicted transcript sequences from the *S. tuberosum* genome [28].

We found that each of the three assemblies shows at least 95 % of Buscos as complete, with just a small difference of only 2 % to 3 % missing. The results are shown in Figure 4.

We aligned the *S. tuberosum* representative transcript sequences to each genome assembly using Blast [46] and then measured how much of each transcript sequence was represented in the assembly according to various minimum percent-

age identity cutoffs. As expected when comparing between species, as the threshold approaches 100 % nucleotide identity the transcript completeness drops closer to zero. Using a threshold between 96 % to 98 % we find the median transcript completeness is highest in `discovar-mp-dt-bn`, followed by `falcon-dt-bn`, and then `supernova-bn`. However, the difference between the assemblies is small, Figure 5 shows a box and whisker plot of completeness of the representative transcript sequences.

Local accuracy

As BACs are easier to assemble due to smaller size and a much more limited amount of repetitive DNA content than a whole genome, we assessed the performance of our three assemblies at a local scale using BAC assemblies. We randomly selected, sequenced, and assembled 96 BAC clones from *S. verrucosum* BAC library. We chose 20 high-quality BAC assemblies (single scaffolds/contigs with Illumina or PacBio) to measure the accuracy of the whole genome assemblies.

We used dnadiff [47] to compare the BAC sequences to the `supernova-bn`, `discovar-mp-dt-bn`, and `falcon-dt-bn` assemblies finding sequence identities of 99.40 %, 99.97 %, and 99.87 %, respectively. As in the previous section, the `discovar-mp-dt-bn` assembly shows the highest accuracy, with `supernova-bn` the lowest, though the differences are small.

To illustrate the performance of the different technologies sequencing different genomic features we mapped whole genome reads and assemblies to single BACs as shown in Figure 3. None of our three whole genome assemblies are able to reconstruct BAC 22; each breaking at a large (more than 12 kbp) repeat. The Discovar library (paired-end), mate-pair library and Dovetail library were each mapped and only reads mapping to a high quality and exhibiting up to one mismatch are shown in the figure. The mapping reveals several areas of high repetition, for example the arms and middle of a retrotransposon, and there are areas lacking coverage completely which suggests a sequence which is difficult for our Illumina sequence data to resolve. We also see drops in coverage at some sites with high concentrations of homopolymers, as marked by coloured lines in the GC content, for example an A rich region of ~7 kbp. Interestingly the repeat arms are also rich in homopolymers.

We note that the `discovar-mp-dt-bn` assembly leaves the largest gap around the repeat. The `falcon` assembly was able to completely cover an area with no mapping paired-end Illumina reads which explains some of extra *k*-mer content in Figure 2 noted earlier in this assembly. The `supernova-bn` assembly was

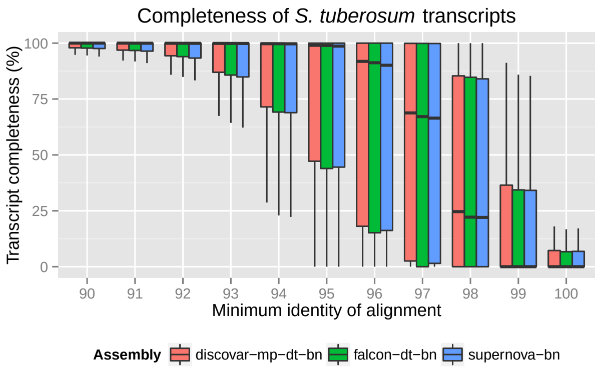

Figure 5. Box and whisker plot showing completeness of the *S. tuberosum* transcripts in `supernova-bn`, `discovar-mp-dt-bn`, and `falcon-dt-bn` with various levels of minimum percentage identity.

able to reconstruct more of the difficult region, but it also contains duplications in the homopolymer rich flanking regions that is not seen in the other assemblies.

The mate-pair library was not able to scaffold the `discovar` contigs due to the size of this repeat being larger than its 10 kbp insert size. The mate-pair fragments also map to a great depth in the repeat. Dovetail data, however, shows a much smoother fragment distribution and was able to scaffold the two `discovar` contigs in the correct order and orientation as it could scaffold up to 50 kbp (the cutoff used by the HiRise scaffolder). However, the gap length was not estimated with Dovetail and was arbitrarily set to 100 Ns when in reality the gap is over 12 000 bp long. While BioNano software estimates gap sizes, we note that BioNano data was not able to close this particular gap in any of the assemblies.

Long-range accuracy using synteny to *S. tuberosum*

As all our assemblies are *de novo*, in the sense that we used no prior information from other Solanaceae genomes, we reasoned that more accurate long range scaffolding would be apparent as longer syntenic blocks to a closely related species. We used nucmer [47] to analyse the synteny of our assemblies to the pseudomolecules of the *S. tuberosum* genome [48]. Figure 6 shows the mummer plot for chromosome 11 of *S. tuberosum* against our three assemblies. We saw the `falcon-dt-bn` assembly showed the best synteny with the `discovar-mp-dt-bn` being the worst. The plots for the remaining chromosomes are shown in Supplemental Figures S3.2, S3.3, and S3.4.

Using synteny we identified two cases of chimerism, i.e. scaffolds that align well to two different pseudomolecules of *S. tuberosum* genome. Both cases are in `discovar-mp-dt-bn` but not `falcon-dt-bn`. The first 1.5 Mbp of scaffold ScEqE3Q_528 maps to pseudomolecule 7 while the last 2.9 Mbp map to pseudomolecule 2 in the *S. tuberosum* genome. There is no conflict reported with the BioNano Genomics optical map in this area, but we can exclude the possibility that these are real chromosome structural arrangements in *S. verruscosum* because we have GbS markers on each end of this scaffold which also map in an *S. verrucosum* cross to these different linkage groups (López-Girona unpublished). The other case is a scaffold ScEqE3Q_633 in which the first 1.4 Mbp map to pseudomolecule 8 and the remainder to pseudomolecule 3, here BioNano Genomics does report a conflict which would highlight this error, and *S. verrucosum* genetic markers also support the chimera classification.

Discussion

The quality and quantity of DNA available, whether it is from fresh or frozen tissue, and ease of its extraction will often dictate which preparation and sequencing technologies are feasible to use. Budget constraints do play a large part in the choice of technologies to be adopted for any genome project. Assembly and scaffolding methods are often effectively the choice of sequencing method, but the properties of the genome will also affect the results. Interestingly, none of the assembly approaches we used lead to a "bad assembly" e.g. one that fails to assemble large parts of the genome or makes many systematic errors (as seen in many early short read assemblies). This speaks to the tremendous progress made in improved sequencing technologies and assembly algorithms. Instead they differ mostly in the length of the ungapped sequence and scaffolds, with much smaller differences in missing sequence and gene content, duplicated regions, and per base accuracy.

A Discovar assembly is the cheapest and easiest to construct, and the resulting assembly is very accurate, albeit highly frag-

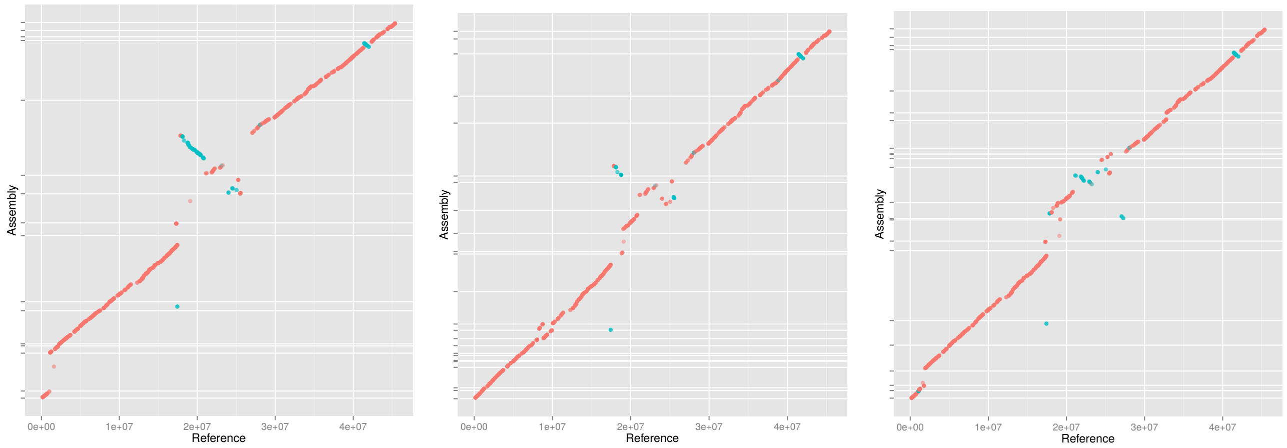

(a) Chromosome 11 of `discovar-mp-dt-bn`. **(b)** Chromosome 11 of `supernova-bn`. **(c)** Chromosome 11 of `falcon-dt-bn`.

Figure 6. Mummer plots showing alignment to chromosome 11 of the *S. tuberosum* reference version 4.03. The *S. tuberosum* reference is shown on the x-axis and assembly scaffolds on the y-axis. Alignments shown are at least 10 kbp long and 90 % identical.

Assembly	Paired-end	Mate-pair	PacBio	Chromium	Dovetail	BioNano	HiSeq 2500	MiSeq	PacBio RSII	**Total (USD)**
`discovar`	✗						✗			3,273
`discovar-mp`	✗	✗					✗	✗		7,854
`discovar-mp-bn`	✗	✗				✗	✗	✗		8,803
`discovar-mp-dt`	✗	✗			✗		✗✗	✗		32,793
`discovar-mp-dt-bn`	✗	✗			✗	✗	✗✗	✗		33,742
`falcon`			✗						✗	25,499
`falcon-bn`			✗			✗			✗	26,448
`falcon-dt`			✗		✗		✗		✗	50,438
`falcon-dt-bn`			✗		✗	✗	✗		✗	51,387
`supernova`				✗			✗			4,299
`supernova-bn`				✗		✗	✗			5,248
**Cost (USD)**	209	595	474	1,235*	21,875	949*	3,064	3,986	25,025	

Table 2. The overall cost of each assembly project. We show which library preparations and sequencing runs are required for each assembly with a checkmark (✗). Individual costs are given at the bottom, and total costs of each assembly on the right. All costs are according to Duke University as of April 2017 and in USD, except those marked with a * which were according to the Earlham Institute and converted from GBP to USD at an exchange rate of 0.804 GBP/USD. Paired-end, mate-pair, PacBio, and Chromium are library preparations including DNA extraction. Dovetail includes Chicago library preparation and HiRise scaffolding. BioNano is the cost of building the optical map. HiSeq2500 is for a rapid run half flowcell (one lane) with 250 bp reads. MiSeq is for two runs with 300 bp reads. PacBio RSII is for 65 SMRT cells.

mented. Adding a long mate-pair library is a proven method of increasing the contiguity of a short read assembly by scaffolding. The 10x Genomics based assembly using Supernova was as easy to obtain as the Discovar assembly. The two most remarkable features of this assembly are the low cost and input DNA requirement: for only slightly higher cost than a Discovar assembly, and considerably less than with only one long mate-pair library, we obtained an assembly comparable to what one would expect from multiple long mate-pair libraries.

Our PacBio assembly using Falcon achieved contiguity similar to that of `discovar-mp` (Discovar plus long mate-pair scaffolding). PacBio sequencing has a considerably higher cost and material requirement than Illumina sequencing, but the `falcon` assembly contains truly contiguous sequence as opposed to `discovar-mp` which contains gaps patched with Ns. The PacBio read lengths (N50=13.5 kbp) were similar to the insert size of mp library (mean 10 kb), and the read coverage was higher for PacBio (50×) than for the mp data (15×), but PacBio contigs (N50=712 kbp) are slightly shorter than the `discovar-mp` scaffolds (N50=858 kbp).

The addition of Dovetail showed the most striking increase in contiguity by scaffolding. We note that our Dovetail scaffolds provided the order and orientation of the constituent contigs but no estimate for the length of the gaps between them. This should be taken into consideration if true physical length of sequences is important, and for specific downstream uses. Both Illumina (Discovar+mp) and PacBio (Falcon) assemblies are amenable to the addition of Dovetail, but the scaffolds produced from the Falcon contigs (4× increase) were not as long as those from the Illumina assembly (5.5× increase). This could be because while the Falcon assembly has been polished with PacBio reads, it retains some PacBio errors and so some Dovetail (Illumina) reads do not pass stringent mapping filters. If true, Pilon polishing with Illumina reads could help, as it improved the *k*-mer spectra (Figure 2).

With BioNano Genomics restriction enzyme digest based optical maps we obtained less (~2× increase) scaffolding improvement than with Dovetail (4× to 5.5× increase). This could be due to three issues: first that assembly gaps are not correctly sized which prevents real, and *in silico*, restriction maps matching (as information is purely encoded in the distances between sites). We see that the ungapped PacBio assemblies improve more than scaffolded Illumina, and Dovetail scaffolds (with arbitrary 100 bp gaps) hardly increase at all. Secondly, because the method produces low information density (one enzyme site per ~12 kbp) long fragments with many sites are need to create significant matches, and our DNA was not sufficiently long (best run N50 was 240 kbp). Longer DNA (over 300 kbp), and perhaps multiple enzyme maps with iterative scaffolding could have improved the results. Thirdly we observe that the *in silico* restriction rates for Illumina and PacBio assemblies are similar (8.1 s to 8.4 sites /100 kbp) whereas the actual observed rates from the physical map is much lower at 6.8 sites/100 kbp, suggesting that there could be a fraction of the genome missing from our assemblies which is very low in sites such as centromeric or telomeric regions where the BioNano Genomics map can not scaffold through.

Gapfilling using PBJelly offers an attractive method of using the long read data from PacBio to improve an existing Illumina based assembly. This closed many of the gaps in the scaffolds thereby decreasing the fraction of unknown sequence (Ns) and also increasing the contiguity. The increase in contiguity of the 10x Genomics assembly was the highest. It will be intriguing to see if an assembly approach combining Chromium data with long reads (directly on the assembly graph) can combine the best attributes of both data types to resolve complex regions.

Analysis of the *k*-mer content of the `supernova`, `discovar`, and `falcon` assemblies showed that the *k*-mer spectra of each assembly is very clean. We see slightly higher level of sequence duplication in the `supernova` assembly, and to a lesser extent in the `falcon` assembly. All three assembly algorithms are diploid aware, meaning they are able to preserve both haplotypes. The gene content of each assembly was very similar with all three of our long assemblies showing a high percentage of the expected genes. The 10x Genomics based assembly showed a slightly lower count in both of our assessments but the difference is very small.

We used multiple BAC assemblies of ~100 kb insert size to illustrate the technical limitations of each method. Short read methods cannot resolve many areas of repetition within a WGS assembly. This is especially noticeable in a plant genome with higher repeat content, and is one of the major reasons for breaks in contiguity in these assemblies. In our example in Figure 3, the long mate-pair library alone is not sufficient. It takes the larger fragment lengths within the Dovetail Chicago library to finally make the join in the whole genome assembly.

Long read technologies do not suffer as much with repeats and, in the case of PacBio, tend to have more random rather than systematic errors [49]. We can see in our examplar that the `falcon` assembly covers some of the repetitive region. The underlying BAC assembly was also obtained with PacBio and gave us a single true contig for the entire BAC. On close inspection we noticed that difficult region was spanned by reads of length 22 kbp to 26 kbp. This shows that long reads are certainly able to span such regions of difficulty, and to assemble them.

Recently ultra-long reads with an N50 of 99.7 kbp (max. 882 kbp) with ~92 % accuracy have been produced with the new MinION R9.4 chemistry using high molecular weight DNA from a human sample [12]. If this is also achievable on plant material the remaining (mostly repetitive) fraction of genomes should become visible. The recent *S. penellii* Nanopore assembly [50] reported average read length 12.7 kbp and error rate of 18 % to 20 %.

To evaluate the longer range accuracy of our genome assemblies we compared them to the closely related *S. tuberosum* pseudomolecule assembly, which revealed good synteny with all three of our longest assemblies (`discovar-mp-dt-bn`, `falcon-dt-bn` and `supernova`). There are some disagreements especially in the centromeric areas, but as these appeared in all assemblies these could illustrate real structural variation. We detected two chimeric scaffolds in the `discovar-mp-dt-bn` assembly but neither is present in the `falcon-dt-bn`. The two Dovetail scaffolding processes shared the same Hi-C sequence data but were conducted many months apart (`discovar-mp` first and later `falcon`), and used different versions of Dovetail's proprietary HiRise software, versions 0.9.6 and 1.3.0, respectively, which might have affected the results. On detailed examination we see that the ScEqE3Q__528 scaffold chimeric join is made by Dovetail hopping through a fragmented area of short (1 kbp to 2 kbp) contigs. Such small contigs do not exist in the Falcon assembly, which maybe why we do not find chimeras. BioNano Genomics finds it hard to map to areas with many Dovetail gaps (as these are set to an arbitrary 100 bp size), and this region also has a high enzyme nicking rate (nearly twice the genome average), including two areas where nicks are less than 200 bp apart and so would be optically merged. In scaffold ScEqE3Q__633 we detect that `discovar-mp` scaffold123 was correctly split by Dovetail data as chimeric (also highlighted by BioNano Genomics and genetic markers) but the scaffold was not broken at the exact chimeric join, and the remaining sequence from the wrong chromosome was sufficient for Dovetail to propagate the error. Whilst we did not detect a high level of systematic errors in any of our assembly methods, the importance of using BioNano Genomics and genetic markers to identify chimeras that then can be broken is apparent.

Library	Tissue type	Material/DNA amount	HMW	Fragment length (bp)
TALL	Frozen	3 µg	No	700
Discovar	Frozen	0.6 µg	No	500
Mate-pair	Frozen	4 µg	No	10 000
PacBio	Young frozen	5 g	No	20 000
BioNano	Young fresh	2.5 µg	Yes	>100 000
Dovetail	Fresh	20 g	Yes	>100 000
Chromium	Flash frozen	0.5 g	Yes	>100 000

Table 3. Material requirements for each library. Amounts in grams are for fresh/frozen material and amounts in micrograms for DNA. In each case where frozen or flash frozen is stated, fresh material is also acceptable.

Even though we found some surprisingly small differences between assemblies of *S. verrucosum*, this is an inbred diploid potato species, with a medium size genome and is in no way exceptional. As there are about 300,000 angiosperms alone [51] we remind the reader, that many factors e.g. genome size, the ease of high quality HMW DNA extraction, the types of repeat content, polyploidy or heterozygosity may pose additional hurdles affecting the choice of technology and how well they will perform. Heterozygosity, in particular, complicates the assembly process and if individual haplotypes are desired this places limitations on which strategies can be used. The careful choice of sample where possible, such as a highly inbred plant or doubled haploid, can remove or minimisethese problems. This approach was also adopted for the potato DM reference, whereby a completely homozygous "doubled monoploid" was used as the heterozygous diploid RH genotype originally selected for sequencing proved difficult to assemble due to the extremely high level of heterozygosity. Newer methods have recently been developed to assemble diploid genomes into chromosome scale phase blocks [52] or even to exploit the haplotype diversity using a "trio binning" approach developed in [53], so we expect to see more true diploid assemblies in the near future.

Materials and Methods

Project requirements

Each of the assembly methods we have used comes with its own requirements. We have broken this down into material requirements, that is plant and DNA material, monetary requirements, that is the cost of preparation and sequencing, and computational requirements. Table 3 lists the material requirements for each library.

We calculated costs taking into consideration the costs of consumables, laboratory time, and machine overheads, but not bioinformatics time. For sequencing costs we used the Duke University cost as much as possible to provide comparative figures. Since several of the projects share common methods, such as sequencing a lane on a HiSeq 2500, we have broken down the costs into individual components. See Table 2 for our full costs calculations.

In many cases the assemblies can be performed with modest scientific computing facilities. In some cases, notably for Supernova, a very large amount of memory is required. In this case the computing requirement will not be available to most laboratories and will need to be sourced elsewhere. Table 4 shows the computational requirements of each assembly method.

Name of assembly	Approximate runtime	Peak memory	Average memory	System
Supernova	3 d	1300 GB		Large memory
Canu (Uncorr)	12 d	47 GB	20 GB	HPC cluster
Canu (Corr)	4 d	34 GB	14 GB	HPC cluster
Falcon	5 d	120 GB	60 GB	Large memory
HGAP	2 m	280 GB		Large memory
Discovar	22 h	260 GB	134 GB	Large memory
ABySS	1 w	64 GB		HPC cluster
BioNano (Asm)	8 h	64 GB	64 GB	HPC cluster
BioNano (Scaf)	1 d	64 GB	64 GB	HPC cluster

Table 4. Computational requirements.

Library preparation and sequencing

In this section we briefly describe methods for library preparation and sequencing. For a comprehensive description, please see the supplementary material.

S. verrucosum accesssion Ver-54 was grown in the glass house in James Hutton Institute in Scotland. Both fresh and frozen leaves from this accession and its clones were used for DNA extraction.

The TALL library was prepared using 3 µg of DNA and fragments of 650 bp were sequenced with a HiSeq2500 with a 2×150 bp read metric. The Discovar library was prepared using 600 ng of DNA and fragments of 500 bp were sequenced with a HiSeq2500 with a 2×250 bp read metric.

The mate-pair library was prepared using 4 µg of DNA and fragments of 10 kbp were circularised, fragmented and sequenced on a MiSeq with a 2×300 bp read metric [54].

A PacBio library was prepared using 5 g of frozen leaf material. A 20 kbp fragment length library was prepared according to manufacturer's instructions and sequenced on 65 SMRT cells with the P6C4 chemistry on a PacBio RSII.

The 10x Chromium library was prepared according to the manufacturer's instructions and sequenced on a HiSeq2500 with a 2×250 bp read metric.

For BioNano, DNA was extracted using the IrysPrep protocol. 300 ng was used in the Nick, Label, Repair and Stain reaction and loaded onto a single flow cell on a BioNano chip. The chip was run eight times to generate 252 Gb of raw data.

Assembly and evaluation

All tools and scripts that were used to perform the evaluation and produce the figures are available on GitHub in the georgek/potato-figures repository.

We used Rampart (Rampart, RRID:SCR_016742) [55] to run ABySS [33] multiple times with different k values. Discovar *de novo* was run with normal parameters.

Long mate-pair reads were first processed with NextClip (NextClip, RRID:SCR_005465) [56] to remove the Nextera adapter. Soapdenovo2 was then used to perform scaffolding with both the paired-end and mate-pair libraries.

k-mer content was analysed with the `kat comp` tool [43] (KAT, RRID:SCR_016741). We used default parameters with manually adjusted plot axes to show the relevant information.

We used the Busco core plant dataset to evaluate the gene content. The *S. tuberosum* representative transcripts (`PGSC_DM_V403_representative_genes` from `http://solanaceae.plantbiology.msu.edu/pgsc_download.shtml`) were aligned to the assemblies using Blast and the coverage of transcripts at various thresholds using a tool we developed.

The BACs were sequenced with the Earlham Institute BAC pipeline [57] and were assembled with Discovar *de novo* using

normal parameters after filtering for *E. coli* and the BAC vector. The PacBio BAC was assembled using HGAP3 [36]. We used GNU parallel [58] for concurrent assembly and analysis.

20 BACs which assembled into a single contig were selected to use as a reference. These BACs are non-redundant to the extent that they do not share any lengths of sequence of more than 95 % identity and over 5000 bp long. Short reads were aligned to the BACs using Bowtie2 [59] with default parameters. The assemblies were mapped to the BACs using `bwa mem` [60]. The mapped sequences were sorted and filtered for quality using sambamba [61]. Fragment coverage was calculated using samtools [62] and bedtools [63].

Synteny was analysed with mummer [64]. We used nucmer to align the assemblies to the *S. tuberosum* reference v4.04 [65]. Alignments less than 10 kbp and 90 % identity were filtered out.

Availability of Supporting Data

All read data generated in this study have been submitted to the EMBL-EBI European Nucleotide Archive under the project PRJEB20860. Archival copy of the code, assemblies and other data are available in the GigaScience GigaDB repository [66].

Acknowledgements

We thank Lawrence Percival-Alwyn and Walter Verweij for their assistance in library preparation and analysis, and Michael Bevan for critical reading of this manuscript. This work was funded with BBSRC project grants (BB/K019325/1) and (BB/K019090/1). This work was strategically funded by the BBSRC, Core Strategic Programme Grant (BB/CSP17270/1) at the Earlham Institute. High-throughput sequencing and library construction was delivered via the BBSRC National Capability in Genomics (BB/CCG1720/1) at the Earlham Institute (EI, formerly The Genome Analysis Centre, Norwich), by members of the Platforms and Pipelines Group. This research was supported in part by the NBI Computing infrastructure for Science (CiS) group through the HPC cluster and UV systems. We thank Duke University for providing sequencing costs via Dugsim (`https://dugsim.net/`).

Authors' contributions: GB, ELG, IH, and GW prepared the sample. MDC, GK, and PP designed the analysis. DB, GB, FC, ELG, MG, DH, IH, AL, and IM constructed libraries and performed sequencing. GK and PP made the assemblies and GK, ELG, and PP performed the evaluation. MDC, GK, GB, ELG and PP wrote and prepared the manuscript. All authors read and approved the final manuscript.

References

1. The 1000 Genomes Project Consortium. An Integrated Map of Genetic Variation from 1,092 Human Genomes. Nature 2012 Nov;491(7422):56–65.
2. Genome 10K Community of Scientists. Genome 10K: A Proposal to Obtain Whole-Genome Sequence for 10,000 Vertebrate Species. Journal of Heredity 2009;100(6):659.
3. Goodwin S, McPherson JD, McCombie WR. Coming of Age: Ten Years of Next-Generation Sequencing Technologies. Nature Reviews Genetics 2016 Jun;17(6):333–351.
4. Doyle JM, Katzner TE, Bloom PH, Ji Y, Wijayawardena BK, DeWoody JA. The Genome Sequence of a Widespread Apex Predator, the Golden Eagle (*Aquila chrysaetos*). PLOS ONE 2014 Apr;9(4):e95599.
5. Callaway E. 'Platinum' Genome Takes on Disease. Nature News 2014 Nov;515(7527):323.
6. Genome in a Bottle—A Human DNA Standard. Nature Biotech 2015 Jul;33(7):675–675.
7. Li R, Zhu H, Ruan J, Qian W, Fang X, Shi Z, et al. *De novo* Assembly of Human Genomes with Massively Parallel Short Read Sequencing. Genome Research 2010 Jan;20(2):265–272.
8. Mostovoy Y, Levy-Sakin M, Lam J, Lam ET, Hastie AR, Marks P, et al. A Hybrid Approach for *de novo* Human Genome Sequence Assembly and Phasing. Nature Methods 2016 Jul;13(7):587–590.
9. Chaisson MJP, Huddleston J, Dennis MY, Sudmant PH, Malig M, Hormozdiari F, et al. Resolving the Complexity of the Human Genome Using Single-Molecule Sequencing. Nature 2015 Jan;517(7536):608–611.
10. Rothberg JM, Hinz W, Rearick TM, Schultz J, Mileski W, Davey M, et al. An Integrated Semiconductor Device Enabling Non-Optical Genome Sequencing. Nature 2011 Jul;475(7356):348–352.
11. Weisenfeld NI, Kumar V, Shah P, Church DM, Jaffe DB. Direct Determination of Diploid Genome Sequences. Genome Research 2017 Apr;.
12. Jain M, Koren S, Miga KH, Quick J, Rand AC, Sasani TA, et al. Nanopore Sequencing and Assembly of a Human Genome with Ultra-Long Reads. Nature Biotechnology 2018;.
13. Pendleton M, Sebra R, Pang AWC, Ummat A, Franzen O, Rausch T, et al. Assembly and Diploid Architecture of an Individual Human Genome via Single-Molecule Technologies. Nature Methods 2015 Aug;12(8):780–786.
14. Friar EA. Isolation of DNA from Plants with Large Amounts of Secondary Metabolites. Methods in Enzymology 2005 Jan;395:1–12.
15. Jiao WB, Schneeberger K. The Impact of Third Generation Genomic Technologies on Plant Genome Assembly. Current Opinion in Plant Biology 2017 Apr;36:64–70.
16. Choulet F, Alberti A, Theil S, Glover N, Barbe V, Daron J, et al. Structural and Functional Partitioning of Bread Wheat Chromosome 3B. Science 2014 jul;345(6194):1249721–1249721.
17. Mascher M, Gundlach H, Himmelbach A, Beier S, Twardziok SO, Wicker T, et al. A Chromosome Conformation Capture Ordered Sequence of the Barley Genome. Nature 2017 Apr;544(7651):427–433.
18. Li R, Fan W, Tian G, Zhu H, He L, Cai J, et al. The Sequence and *de novo* Assembly of the Giant Panda Genome. Nature 2010 Jan;463(7279):311–317.
19. Bevan MW, Uauy C, Wulff BBH, Zhou J, Krasileva K, Clark MD. Genomic Innovation for Crop Improvement. Nature 2017 Mar;543(7645):346–354.
20. Jackman SD, Warren RL, Gibb EA, Vandervalk BP, Mohamadi H, Chu J, et al. Organellar Genomes of White Spruce (*Picea glauca*): Assembly and Annotation. Genome Biology and Evolution 2016 Jan;8(1):29–41.
21. Ogihara Y, Yamazaki Y, Murai K, Kanno A, Terachi T, Shiina T, et al. Structural Dynamics of Cereal Mitochondrial Genomes as Revealed by Complete Nucleotide Sequencing of the Wheat Mitochondrial Genome. Nucleic Acids Research 2005;33(19):6235.
22. Anderson S, Bankier AT, Barrell BG, de Bruijn MHL, Coulson AR, Drouin J, et al. Sequence and Organization of the Human Mitochondrial Genome. Nature 1981 Apr;290(5806):457–465.
23. Fleischmann A, Michael TP, Rivadavia F, Sousa A, Wang W, Temsch EM, et al. Evolution of Genome Size and Chromosome Number in the Carnivorous Plant Genus *Genlisea* (Lentibulariaceae), with a New Estimate of the Minimum Genome Size in Angiosperms. Annals of Botany 2014 Dec;114(8):1651–1663.

24. Pellicer J, Fay MF, Leitch IJ. The Largest Eukaryotic Genome of Them All? Botanical Journal of the Linnean Society 2010 Sep;164(1):10–15.
25. Cai D, Rodríguez F, Teng Y, Ané C, Bonierbale M, Mueller LA, et al. Single Copy Nuclear Gene Analysis of Polyploidy in Wild Potatoes (*Solanum* Section *Petota*). BMC Evolutionary Biology 2012;12:70.
26. Pendinen G, Gavrilenko T, Jiang J, Spooner DM. Allopolyploid Speciation of the Mexican Tetraploid Potato Species *Solanum stoloniferum* and *S. hjertingii* Revealed by Genomic *in situ* Hybridization. Genome 2008 Aug;51(9):714–720.
27. Spooner D, Castillo R. Reexamination of series relationships of South American wild potatoes (Solanaceae: Solanum sect. Petota): evidence from chloroplast DNA restriction site variation. American Journal of Botany 1997;84(5):671. http://www.amjbot.org/content/84/5/671.abstract.
28. The Potato Genome Sequencing Consortium. Genome Sequence and Analysis of the Tuber Crop Potato. Nature 2011;475(7355):189–195.
29. The Tomato Genome Consortium. The Tomato Genome Sequence Provides Insights into Fleshy Fruit Evolution. Nature 2012 May;485(7400):635–641.
30. Hirakawa H, Shirasawa K, Miyatake K, Nunome T, Negoro S, Ohyama A, et al. Draft Genome Sequence of Eggplant (*Solanum melongena* L.): The Representative Solanum Species Indigenous to the Old World. DNA Research 2014 Dec;21(6):649–660.
31. Kim S, Park M, Yeom SI, Kim YM, Lee JM, Lee HA, et al. Genome Sequence of the Hot Pepper Provides Insights into the Evolution of Pungency in *Capsicum* Species. Nature Genetics 2014 Mar;46(3):270–278.
32. Simpson JT, Durbin R. Efficient *de novo* Assembly of Large Genomes using Compressed Data Structures. Genome Research 2012;22(3):549–556.
33. Simpson JT, Wong K, Jackman SD, Schein JE, Jones SJ, Birol I. ABySS: A Parallel Assembler for Short Read Sequence Data. Genome Research 2009;19(6):1117–1123.
34. Weisenfeld NI, Yin S, Sharpe T, Lau B, Hegarty R, Holmes L, et al. Comprehensive Variation Discovery in Single Human Genomes. Nature Genetics 2014 Dec;46(12):1350–1355.
35. Luo R, Liu B, Xie Y, Li Z, Huang W, Yuan J, et al. SOAPdenovo2: An Empirically Improved Memory-Efficient Short-Read *de novo* Assembler. GigaScience 2012;1:18.
36. Chin CS, Alexander DH, Marks P, Klammer AA, Drake J, Heiner C, et al. Nonhybrid, Finished Microbial Genome Assemblies from Long-Read SMRT Sequencing Data. Nature Methods 2013 Jun;10(6):563–569.
37. Koren S, Walenz BP, Berlin K, Miller JR, Bergman NH, Phillippy AM. Canu: Scalable and Accurate Long-Read Assembly via Adaptive *k*-mer Weighting and Repeat Separation. Genome Research 2017 Jan;27(5):722–736.
38. Chin CS, Peluso P, Sedlazeck FJ, Nattestad M, Concepcion GT, Clum A, et al. Phased Diploid Genome Assembly with Single-Molecule Real-Time Sequencing. Nature Methods 2016 Dec;13(12):1050–1054.
39. Li H. Minimap and miniasm: fast mapping and de novo assembly for noisy long sequences. Bioinformatics 2016;32(14):2103–2110. http://dx.doi.org/10.1093/bioinformatics/btw152.
40. Putnam NH, O'Connell BL, Stites JC, Rice BJ, Blanchette M, Calef R, et al. Chromosome-Scale Shotgun Assembly Using an *in vitro* Method for Long-Range Linkage. Genome Research 2016 Jan;26(3):342–350.
41. Hastie AR, Dong L, Smith A, Finklestein J, Lam ET, Huo N, et al. Rapid Genome Mapping in Nanochannel Arrays for Highly Complete and Accurate *de novo* Sequence Assembly of the Complex *Aegilops tauschii* Genome. PLOS ONE 2013 Feb;8(2):e55864.
42. English AC, Richards S, Han Y, Wang M, Vee V, Qu J, et al. Mind the Gap: Upgrading Genomes with Pacific Biosciences RS Long-Read Sequencing Technology. PLOS ONE 2012 Nov;7(11):e47768.
43. Mapleson D, Accinelli GG, Kettleborough G, Wright J, Clavijo BJ. KAT: A K-mer Analysis Toolkit to Quality Control NGS Datasets and Genome Assemblies. Bioinformatics 2016;.
44. Walker BJ, Abeel T, Shea T, Priest M, Abouelliel A, Sakthikumar S, et al. Pilon: An Integrated Tool for Comprehensive Microbial Variant Detection and Genome Assembly Improvement. PLOS ONE 2014 11;9(11):1–14.
45. Simão FA, Waterhouse RM, Ioannidis P, Kriventseva EV, Zdobnov EM. BUSCO: Assessing Genome Assembly and Annotation Completeness with Single-Copy Orthologs. Bioinformatics 2015 Oct;31(19):3210–3212.
46. Altschul SF, Gish W, Miller W, Myers EW, Lipman DJ. Basic Local Alignment Search Tool. Journal of Molecular Biology 1990 Oct;215(3):403–410.
47. Kurtz S, Phillippy A, Delcher AL, Smoot M, Shumway M, Antonescu C, et al. Versatile and Open Software for Comparing Large Genomes. Genome Biology 2004;5(2):R12.
48. Sharma SK, Bolser D, de Boer J, Sønderkær M, Amoros W, Carboni MF, et al. Construction of Reference Chromosome-Scale Pseudomolecules for Potato: Integrating the Potato Genome with Genetic and Physical Maps. G3: Genes, Genomes, Genetics 2013;3(11):2031–2047. http://www.g3journal.org/content/3/11/2031.
49. Carneiro MO, Russ C, Ross MG, Gabriel SB, Nusbaum C, DePristo MA. Pacific biosciences sequencing technology for genotyping and variation discovery in human data. BMC Genomics 2012;13(1):375. http://bmcgenomics.biomedcentral.com/articles/10.1186/1471-2164-13-375.
50. Schmidt MH, Vogel A, Denton AK, Istace B, Wormit A, van de Geest H, et al. De novo Assembly of a New Solanum pennellii Accession Using Nanopore Sequencing. The Plant Cell 2017;http://www.plantcell.org/content/early/2017/10/12/tpc.17.00521.
51. Christenhusz MJM, Byng JW. The number of known plants species in the world and its annual increase. Phytotaxa 2016;261(3):201. https://biotaxa.org/Phytotaxa/article/view/phytotaxa.261.3.1.
52. Kronenberg ZN, Hall RJ, Hiendleder S, Smith TPL, Sullivan ST, Williams JL, et al. FALCON-Phase: Integrating PacBio and Hi-C data for phased diploid genomes. bioRxiv 2018;https://www.biorxiv.org/content/early/2018/05/21/327064.
53. Koren S, Rhie A, Walenz BP, Dilthey AT, Bickhart DM, Kingan SB, et al. Complete assembly of parental haplotypes with trio binning. bioRxiv 2018;https://www.biorxiv.org/content/early/2018/02/26/271486.
54. Heavens D, Accinelli GG, Clavijo B, Clark MD. A Method to Simultaneously Construct up to 12 Differently Sized Illumina Nextera Long Mate Pair Libraries with Reduced DNA Input, Time, and Cost. BioTechniques 2015 Jul;59(1):42–45.
55. Mapleson D, Drou N, Swarbreck D. Rampart: A Workflow Management System for *de novo* Genome Assembly. Bioinformatics 2015 Jun;31(11):1824–1826.
56. Leggett RM, Clavijo BJ, Clissold L, Clark MD, Caccamo M. NextClip: An Analysis and Read Preparation Tool for Nextera Long Mate Pair Libraries. Bioinformatics 2014 feb;30(4):566–568.
57. Beier S, Himmelbach A, Colmsee C, Zhang XQ, Barrero RA, Zhang Q, et al. Construction of a Map-Based Reference Genome Sequence for Barley, *Hordeum vulgare* L. Scientific Data 2017 Apr;4.

58. Tange O. GNU Parallel—The Command-Line Power Tool. ;login: The USENIX Magazine 2011 Feb;36(1):42–47. http://www.gnu.org/s/parallel.
59. Langmead B, Salzberg SL. Fast Gapped-Read Alignment with Bowtie 2. Nature Methods 2012;9(4):357–359.
60. Li H. Aligning Sequence Reads, Clone Sequences and Assembly Contigs with BWA-MEM. arXiv preprint arXiv:13033997 2013;.
61. Tarasov A, Vilella AJ, Cuppen E, Nijman IJ, Prins P. Sambamba: Fast Processing of NGS Alignment Formats. Bioinformatics 2015;31(12):2032–2034.
62. Li H, Handsaker B, Wysoker A, Fennell T, Ruan J, Homer N, et al. The Sequence Alignment/Map Format and SAMtools. Bioinformatics 2009;25(16):2078–2079.
63. Quinlan AR, Hall IM. BEDTools: A Flexible Suite of Utilities for Comparing Genomic Features. Bioinformatics 2010;26(6):841–842.
64. Delcher AL, Salzberg SL, Phillippy AM. Using MUMmer to Identify Similar Regions in Large Sequence Sets. Current Protocols in Bioinformatics 2003;p. 10–3.
65. Hardigan MA, Crisovan E, Hamiltion JP, Kim J, Laimbeer P, Leisner CP, et al. Genome Reduction Uncovers a Large Dispensable Genome and Adaptive Role for Copy Number Variation in Asexually Propagated *Solanum tuberosum*. The Plant Cell 2016 Jan;p. TPC2015-00538-RA.
66. Paajanen P, Kettleborough G, López-Girona E, Giolai M, Heavens D, Baker D, et al. Supporting data for “A critical comparison of technologies for a plant genome sequencing project”. GigaScience Database 2018;.

Click here to access/download
Supplementary Material
supplemental.pdf
