# Supplementary material for: A critical comparison of technologies for a plant genome sequencing project
Source: Gigascience. 2019 Jan 9;8(3):giy163. doi: 10.1093/gigascience/giy163 (PMC6423373; doi:10.1093/gigascience/giy163)
Supplement: Supplemental File [file giy163_supplemental_file.pdf]

Supplemental material for *A critical comparison of technologies for a plant genome sequencing project*

Pirita Paajanen[1,3*], George Kettleborough[1*], Elena López-Girona[2,4], Michael Giolai[1], Darren Heavens[1], David Baker[1], Ashleigh Lister[1], Fiorella Cugliandolo[1], Gail Wilde[2], Ingo Hein[2], Iain Macaulay[1], Glenn J. Bryan[2], and Matthew D. Clark[1,5+]

**contributed equally to this work*
+corresponding author: `matt.clark@earlham.ac.uk`

[1] *Earlham Institute, Norwich, UK;* [2] *The James Hutton Institute, Invergowrie, Dundee, UK;* [3] *Department of Cell and Developmental Biology, John Innes Centre, Norwich, UK;* [4] *The New Zealand Institute for Plant & Food Research Limited, Plant & Food Research, Palmerston North, New Zealand;* [5] *Department of Life Sciences, Natural History Museum, London, UK*

Contents

1 DNA Extraction and Library Preparation 3
1.1 Germplasm . . . 3
1.2 DNA extraction . . . 3
1.2.1 CTAB DNA extraction . . . 3
1.2.2 IrysPrep DNA extraction . . . 3
1.3 Illumina library preparation . . . 4
1.3.1 Tight and Long Library (TALL) paired-end library construction protocol . . . 4
1.3.2 Amplification free paired-end library construction (DISCOVAR) . . . 4
1.3.3 Long mate pair library construction protocol . . . 5
1.4 PacBio library preparation . . . 6
1.5 10x library preparation . . . 6
1.6 BioNano preparation . . . 6
1.7 Dovetail extraction and library preparation . . . 6
1.8 BAC library . . . 6

2 Assembly and Scaffolding 8
2.1 Illumina . . . 8
2.1.1 ABySS . . . 8
2.1.2 DISCOVAR . . . 8
2.1.3 Mate-pair scaffolding . . . 8
2.2 PacBio . . . 8
2.2.1 Falcon . . . 8
2.2.2 Canu . . . 9
2.2.3 HGAP . . . 9
2.3 10x Genomics Supernova . . . 9
2.4 BioNano . . . 9
2.4.1 Optical map assembly . . . 10
2.4.2 Scaffolding . . . 10
2.5 Dovetail . . . 10
2.6 BAC assembly . . . 10
2.6.1 Illumina . . . 10
2.6.2 PacBio . . . 10

3 Evaluation 11
3.1 Contiguity . . . 11

3.2 K-mer content . . . 11
3.3 Synteny . . . 11
3.4 Gene content . . . 11
3.5 BUSCO . . . 11

4 Data availability **16**
4.1 Short reads . . . 16
4.2 Long reads . . . 16
4.3 Optical map . . . 16
4.4 Assemblies . . . 16

5 File list **17**

Library name	Library type	Read pair count	Read length (bp)	Insert size (bp)	Read coverage	Fragment coverage
LIB6268	TALL	308 350 323	150	650	137.25	274.50
LIB6487	Mate-pair	44 864 446	300	10 000	39.94	665.64
LIB12786	Discovar	160 305 585	250	500	118.92	118.92
LIB17395	Chicago (Dovetail)	142 321 242	250		105.58	
LIB24104	Chromium (10x)	141 344 719	250	900	104.86	188.74

Table S1.1: Summary of short read library sequencing. Insert size is given where a mode is appropriate.

1 DNA Extraction and Library Preparation

1.1 Germplasm

S. verrucosum accesssion Ver-54 was grown in the glass house in James Hutton Institute in Scotland. Both fresh and frozen leaves from this accession and its clones were used for DNA extraction.

1.2 DNA extraction

1.2.1 CTAB DNA extraction

Genomic DNA was extracted using CTAB lysis, phenol-chloroform, and Qiagen MagAttract HMW DNA Kit (QIAGEN, Manchester, UK) purification. Young frozen *S. verrucosum* Ver-54 leaves (5 g) were ground to a fine powder using a liquid nitrogen cooled pestle and mortar, distributed over two 50 ml falcon tubes and mixed with 20 ml CTAB buffer (100mM Tris-HCl, pH 8.0, 2 % (w/v) CTAB, 1.4 M NaCl, 20 mM EDTA) containing 20 µg/ml proteinase K and incubated at 55 °C for 20 minutes. Next, 0.5 volumes (8 ml) chloroform was added and carefully mixed by 15× inversion followed by centrifugation at 2990×g on an Eppendorf Centrifuge 5810 R (Eppendorf, Stevenage, UK) for 30 minutes. The aqueous phase was carefully transferred into a new tube to which 1 volume phenol/chloroform/isoamyl alcohol was added followed by centrifugation for 30 minutes at 2990×g. The aqueous phase was ethanol precipitated by addition of 3 M sodium acetate (1/10 of DNA volume), (pH 5.2) and 2.5 volumes of ethanol, mixed and precipitated at 2990×g and 4 °C. DNA pellets were washed with ice-cold 70 % ethanol, air dried and resuspended in 350 µl of 1× TE buffer (10 mM Tris-HCl, 1 mM EDTA pH 8.0) containing 10 U RNase One (Promega, Southampton, UK). The DNA was dissolved overnight at 4 °C with occasional mixing by inversion. Finally, we purified the DNA with the QIAGEN MagAttract HMW DNA Kit with minor changes to the manufacture instructions at the beginning; we added 150 µl AL Buffer to 200 µl DNA (10 µg in TE) and 15 µl magnetic bead suspension. From this point we followed the Quick-Start Protocol and we eluted the DNA in 200 µl of 1× TE buffer.

1.2.2 IrysPrep DNA extraction

Fresh young leaves of the *S. verrucosum* Ver-54 accession were collected after 48-hour treatment in the dark. Earlham Institute's Platforms and Pipelines group followed IrysPrep "Fix'n'Blend" Plant DNA extraction protocol supplied by BioNano Genomics. 2.5 g of fresh young leaves were fixed with 2 % formaldehyde. After washing, leaves are disrupted and homogenized in the presence of isolation buffer. The isolation buffer contains PVP10 and BME to prevent oxidation of polyphenols. Triton X-100 is added to facilitate the release of nuclei from the broken cells. The nuclei are then purified on a Percoll cushion. A nuclei phase is taken and washed several times in isolation buffer before embedding into low melting point agarose. 2 plugs of 90 µl were cast using the CHEF Mammalian Genomic DNA Plug Kit (Bio-Rad 170-3591). Once set at 4 °C the plugs were added to a lysis solution containing 200 µl proteinase K (QIAGEN 158920) and 2.5 ml of BioNano lysis buffer in a 50 ml conical tube. These were put at 50 °C for 2 hours on a thermomixer, making a fresh proteinase K solution to incubate overnight. The 50 ml tubes were then removed from the thermomixer for 5 minutes before 50 µl RNAse A (Qiagen158924) was added and the tubes returned to the thermomixer for a further hour at 37 °C.The plugs were then washed 7 times in Wash Buffer supplied in Chef kit and 7 times in 1xTE. One plug was removed and melted for 2 minutes at 70 °C followed by 5 minutes at 43 °C before adding 10 µl of 0.2 U /µl of GELase (Cambio Ltd G31200). After 45 minutes at 43 °C the melted plug was dialysed on a 0.1 µM membrane (Millipore VCWP04700) sitting on 15 ml of 1xTE in a small petri dish. After 2 hours the sample was removed with a wide bore tip and mixed gently 5 times and left overnight at 4 °C.

1.3 Illumina library preparation

1.3.1 Tight and Long Library (TALL) paired-end library construction protocol

DNA was extracted using the CTAB protocol given in Section 1.2.1. A total of 3 µg of DNA was sheared in a 60 µl volume on a Covaris S2 (Covaris, Massachusetts, USA) for 1 cycle of 40 seconds with a duty cycle of 5 %, cycles per burst of 200 and intensity of 3. The fragmented DNA was then subjected to size selection on a Blue Pippin (Sage Science, Beverly, USA). The 40 µl in each of collection wells was replaced with fresh buffer and the separation and elution current checked prior to loading the sample. To 30 µl of the end repaired molecules 10 µl of R2 marker solution was added and then loaded onto a 1.5 % Cassette. The Blue Pippin was configured to collect fragments at 800 bp using the tight settings. Post size selection, the 40 µl from the collection well was recovered and the size isolated estimated on High Sensitivty BioAnalyser Chip and DNA concentration determined using a Qubit HS Assay.

The size selected molecules were then end repaired in 100 µl volume using the NEB End Repair Module (NEB, Hitchin, UK) incubating the reaction at 22 °C for 30 minutes. Post incubation 100 µl beads of CleanPCR beads (GC Biotech, Alphen aan den Rijn, The Netherlands) were added and the DNA precipitated onto the beads. They were then washed twice with 70 % ethanol and the end repaired molecules eluted in 25 µl Nuclease free water (Qiagen, Manchester, UK).

End repaired molecules were then A tailed in 30 µl volume using in the NEB A tailing module (NEB) incubating the reaction at 37 °C for 30 minutes. To the A tailed library molecules 1 µl of an appropriate Illumina TruSeq Index adapter (Illumina, San Diego, USA) was added and mixed then 31 µl of Blunt/ TA ligase (NEB) added and incubated at 22 °C for 10m. Post incubation 5 µl of stop ligation was added and then the reaction incubated at room temperature for 5 minutes. Following this incubation 67 µl beads of CleanPCR beads (GC Biotech, Alphen aan den Rijn, The Netherlands) were added and the DNA precipitated onto the beads. They were then washed twice with 70 % ethanol and the end repaired molecules eluted in 100 µl nuclease free water. Two further 1× CleanPCR bead based purifications were undertaken to remove any adapter dimer molecules that may have formed during the adapter ligation step and the final library eluted in 25 µl Resuspension Buffer (Illumina).

Library QC was performed by running a 1 µl aliquot on a High Sensitivity BioAnalyser chip (Agilent, Stockport, UK) and the DNA concentration measured using the High Sensitivity Qubit (Thermo Fisher, Cambridge, UK). To determine the number of viable library molecules the library was subjected to quantification by the Kappa qPCR Illumina quantification kit (Kapa Biosystems, London, UK) and then sequenced on the Hiseq2500 (Illumina) with a 2×150 bp read metric.

1.3.2 Amplification free paired-end library construction (DISCOVAR)

DNA was extracted using the CTAB protocol given in Section 1.2.1. A total of 600 ng of DNA was sheared in a 60 µl volume on a Covaris S2 (Covaris, Massachusetts, USA) for 1 cycle of 40 seconds with a duty cycle of 5 %, cycles per burst of 200 and intensity of 3. The fragmented molecules were then end repaired in 100 µl volume using the NEB End Repair Module (NEB, Hitchin, UK) incubating the reaction at 22 °C for 30 minutes. Post incubation 58 µl beads of CleanPCR beads (GC Biotech, Alphen aan den Rijn, The Netherlands) were added using a positive displacement pipette to ensure accuracy and the DNA precipitated onto the beads. They were then washed twice with 70 % ethanol and the end repaired molecules eluted in 25 µl Nuclease free water (Qiagen, Manchester, UK).

End repaired molecules were then A tailed in 30 µl volume using in the NEB A tailing module (NEB) incubating the reaction at 37 °C for 30 minutes. To the A tailed library molecules 1 µl of an appropriate Illumina TruSeq Index adapter (Illumina, San Diego, USA) was added and mixed then 31 µl of Blunt/TA ligase (NEB) added and incubated at 22 °C for 10m. Post incubation 5 µl of stop ligation was added and then the reaction incubated at room temperature for 5 minutes. Following this incubation 67 µl beads of CleanPCR beads (GC Biotech, Alphen aan den Rijn, The Netherlands) were added and the DNA precipitated onto the beads. They were then washed twice with 70 % ethanol and the end repaired molecules eluted in 100 µl nuclease free water. Two further CleanPCR bead based purifications were undertaken to remove any adapter dimer molecules that may have formed during the adapter ligation step. The first with 0.9× volume beads, the second with 0.6× and the final library eluted in 25 µl Resuspension Buffer (Illumina).

Library QC was performed by running a 1 µl aliquot on a High Sensitivity BioAnalyser chip (Agilent, Stockport, UK) and the DNA concentration measured using the High Sensitivity Qubit (Thermo Fisher, Cambridge, UK). To determine the number of viable library molecules the library was subjected to quantification by the Kappa qPCR Illumina quantification kit (Kapa Biosystems, London, UK) and then sequenced at a loading concentration of 9 pM on the Hiseq2500s (Illumina) with a 2×250bp read metric.

1.3.3 Long mate pair library construction protocol

DNA was extracted using the CTAB protocol given in Section 1.2.1. For the Tagmentation reactions 4 µg of Genomic DNA was prepared in 308 µl and then 80 µl 5× Tagment Buffer Mate Pair (Illumina, San Diego, USA) added followed by 12 µl Mate Pair Tagmentation Enzyme (Illumina) and the reaction gently vortexed to mix. This was then incubated for 30 minutes at 55 °C, 100 µl of Neutralize Tagment Buffer (Illumina) added and then incubated at room temperature for 5 minutes. A 1× volume bead clean-up was performed with CleanPCR beads (GC Biotech, Alphen aan den Rijn, The Netherlands) and the DNA eluted in 165 µl of Nuclease free Water . A 1 µl aliquot was run on a BioAnalyser 1200 chip and DNA concentration determined using a Qubit HS Assay.

Strand Displacement was performed by combining 162 µl of tagmented DNA, 20 µl 10× Strand Displacement Buffer (Illumina), 8 µl dNTPs (Illumina) and 10 µl Strand Displacement Polymerase (Illumina). This was then incubated at room temperature for 30 minutes. A 0.75× volume bead clean-up was performed with CleanPCR beads and the DNA eluted in 16 µl of Nuclease free Water and the eluted DNA from the 3 µg and 6 µg reactions pooled. A 1 µl aliquot was diluted 1:6 and run on a BioAnalyser 1200 chip chip (Agilent, Stockport, UK) and DNA concentration determined using a Qubit HS Assay (Thermo Fisher, Cambridge, UK).

Size selection was performed on a Sage Science Blue Pippin (Sage Science, Beverly, USA). The 40 µl in the collection well was replaced with fresh buffer and the collection and elution current checked prior to loading the sample. To 30 µl of the pooled Strand Displaced reaction 10 µl of loading solution was added and then loaded onto a 0.75 % Cassette which was configured to collect fragments at 10 kbp using the tight setting. Post size selection, the 40 µl from the collection well was recovered and the DNA concentration determined using a Qubit HS Assay.

Circularisation was performed by combining 40 µl of size fractionated DNA, 12.5 µl of 10× circularisation buffer (Illumina), 3 µl Circularisation Enzyme (Illumina) and 75 µl nuclease free water.

This was then incubated at 30 °C overnight. Linear DNA was digested by adding 3.75 µl Exonuclease (Illumina) and incubating at 37 °C for 30 minutes followed by 70 °C for 30 minutes to denature the enzyme and 5 µl of stop ligation (Illumina) added. During exonuclease treatment 20 µl of M280 Dynabeads (Thermo Fisher) were prepared by washing twice with 100 µl Bead Bind Buffer (Illumina) before resuspending in 130 µl Bead Bind Buffer. Circularised DNA was then sheared in a 130 µl volume on a Covaris S2 for 2 cycles of 37 seconds with a duty cycle of 10 %, cycles per burst of 200 and intensity of 4.

To 130 µl fragmented DNA 130 µl of washed M280 beads was added, mixed and then placed on a lab rotator at room temperature for 20 minutes. Library molecules bound to M280 beads were then washed four times with 200 µl Bead Washer Buffer (Illumina) and twice with 200 µl Resuspension Buffer (Illumina).

A master mix containing 85 µl nuclease free water (Qiagen, Manchester, UK), 10 µl 10× End Repair Reaction Buffer (NEB, Hitchin, UK) and 5 µl end repair enzyme mix (NEB) was prepared and added to the tube, mixed with the beads and incubated at room temperature for 30 minutes. End repaired library molecules bound to M280 beads were then washed four times with 200 µl Bead Washer Buffer and twice with 200 µl Resuspension Buffer.

A master mix containing 25 µl nuclease free water, 3 µl A Tailing 10× Reaction Buffer (NEB) and 2 µl A tailing enzyme mix (NEB) was prepared and added to the tube, mixed with the beads and incubated at 37 °C for 30 minutes. To the A tailed library molecules 1 µl of the appropriate Illumina Index adapter (Illumina) was added and mixed then 31 µl of Blunt/TA ligase (NEB) added and incubated at room temperature for 10 minutes. Post incubation 5 µl of stop ligation added and then the adapter ligated library molecules bound to M280 beads were then washed four times with 200 µl Bead Washer Buffer and twice with 200 µl Resuspension Buffer.

A master mix containing 20 µl nuclease free water, 25 µl 2× Kappa HiFi (Kappa Biosystems) and 5 µl Illumina Primer Cocktail (Illumina) was prepared and added to each tube, mixed with the beads and the contents, including beads, transferred to a 200 µl PCR tube. Each sample was then subjected to amplification on a Veriti Thermal Cycler (Thermo Fisher) with the following conditions:- 98 °C for 3 minutes, 15 cycles of PCR of 98 °C for 10 seconds, 60 °C for 30 seconds, 72 °C for 30 seconds followed by 72 °C for 5 minutes and Hold at 4 °C.

Post amplification the PCR tube was placed on a magnetic plate, the beads allowed to pellet and then 45 µl of the PCR transferred to a 2 ml Lobind Eppendorf Tube. To this 31.5 µl beads of CleanPCR beads were added to precipitate the DNA, the beads washed twice with 70 % ethanol and the final library eluted in 20 µl resuspension buffer. Library QC was performed by running a 1 µl aliquot on a High Sensitivity BioAnalyser chip and the DNA concentration measured using the High Sensitivity Qubit. The quantification of the pool was determined by the Kappa qPCR Illumina quantification kit with the pool run at 10 pM on a MiSeq with a 2×300 bp reads read metric.

1.4 PacBio library preparation

DNA was extracted using the CTAB protocol given in Section 1.2.1. We created a 20 kbp fragment length library according to the manufacturer's instructions as 20 kbp Template Preparation Using BluePippinTM Size-Selection System, and sequenced 65 SMRT cells with the P6C4 chemistry on the PacBio RSII instrument.

The total yield was 32 Gb of Data, the final N50 of read length was 13 499 bp. The total coverage of the raw data was 50×.

1.5 10x library preparation

DNA was extracted using the CTAB protocol given in Section 1.2.1. DNA material was diluted to 1.1ng/ul with EB (Qiagen) and checked with a QuBit Flourometer 2.0 (Invitrogen) using the QuBit dsDNA HS Assay kit. The Chromium User Guide was followed as per the manufacturer's instructions (10X Genomics, PN-120229).

The final library was quantified using qPCR (KAPA Library Quant kit (Illumina), ABI Prism qPCR Mix, Kapa Biosystems). Sizing of the library fragments were checked using a Bioanalyzer (High Sensitivity DNA Reagents, Agilent). Samples were pooled based on the molarities calculated using the two QC measurements.

The library was clustered at 8 pM with a 1 % spike in of PhiX library (Illumina). The pool was run on a HiSeq2500 250 bp Rapid Run V2 mode (Illumina). The following run metrics were applied: Read 1: 250 cycles, Index 1: 8 cycles, Index 2: 0 cycles and Read 2: 250 cycles.

1.6 BioNano preparation

DNA was extracted using the IrysPrep protocol given in Section 1.2.2. A small amount was removed to QC on an Opgen Argus Q-Card and Qubit HS for the DNA concentration. 300 ng of DNA was taken into the NLRS (Nick, Label, Repair and Stain) reaction using 1 µl Nt.BspQI (NEB R0644S). Following the NLRS reaction 16 µl was loaded onto a single flow cell on a BioNano chip. The Chip loading was optimised and run for 30 cycles on the BioNano Irys using ICS1.6. The same chip was run a total of 8 times on each side to generate 252 Gb of raw data with molecule length over 100 kbp with a nick density of 6.79/100 kbp. Images were converted to .bnx files using AutoDetect 2.1.0.6656 before analysis.

HMW DNA was isolated and the nicking endonuclease Nt.BspQ1 (New England BioLabs) was used label high-quality HMW DNA molecules at specific sequence motifs (GCTCTTC). The nicked DNA molecules were then stained according to the instructions of IrysPrep Reagent Kit (BioNano Genomics).

The HMW DNA with flourescent labeles was loaded onto the nanochallen array of the IrysChip (BioNano Genomics) and was automatically imaged by the Irys system (BioNano Genomics). Raw DNA molecules of at least 100 kbp were collected and converted into BNX files by AutoDetect software to obtain basic labeling and DNA length information. The filtered raw DNA molecules in BNX format were aligned, clustered, and assembled into the BNG map by using the BioNano Genomics assembly pipeline as described in previous publications (40, 41). The P value thresholds used for pairwise assembly, extension/refinement, and final refinement stages were 1×109, 1×1010, and 1×1010, respectively. The initial BNG map was then checked for potential chimeric BNG contigs and was further refined. To compare the draft sequence assembly with the BNG map, sequences were digested in silico according to the restriction site of Nt.BspQ1 by using Knickers (BioNano Genomics). The alignment of sequence assemblies with the BNG map was computed with RefAligner, and the visualization of the alignment was performed with snapshot in IrysView (`http://bionanogenomics.com/support/software-downloads/`).

1.7 Dovetail extraction and library preparation

20 g of fresh leaf material was sent to Dovetail Genomics, LLC (Santa Cruz, CA, USA). They extracted HMW DNA that was used to construct a Chicago library.

1.8 BAC library

Very young, partially expanded leaves from dark treated *S. verrucosum* Ver-54 plantlets were harvested and immediately frozen in liquid nitrogen. A 5× pooled BAC library was generated by Bio S&T (Canada) and contained 192 pools of approximately 600 independent recombinants per pool. For the library generation, genomic DNA was subjected to HindIII restriction enzyme digest, ligated in to the vector pindigoBAC-5 (HindIII-Cloning Ready) (Epicentre) and transformed into compatible DH10B cells (Invitrogen). The average insert size was estimated to be 125 kbp following

NotI restriction enzyme digest and PFGE separation of nine randomly selected clones. Each well from one plate was then grown on a single plate and a single colony from each was selected for sequencing.

2 Assembly and Scaffolding

2.1 Illumina

2.1.1 ABySS

The Tight Amplification free Library (TALL) was assembled using ABySS. We used RAMPART [**mapleson_rampart:_2015**] to automate the running of ABySS and select an appropriate k value ($k = 113$ was selected). The configuration files for RAMPART is given in Supplemental File **S1** (`pair_end_potato.xml`. We used ABySS version 1.5.1 and RAMPART version 0.10.3. The command use to run Rampart was:

```
rampart pair_end_potato.xml
```

2.1.2 DISCOVAR

The DISCOVAR library was assembled using DISCOVAR de novo version 51828. We ran it with the following command:

```
DiscovarExp READS=DI_LIB12786_L1_R1.fastq.gz,DI_LIB12786_L1_R2.fastq.gz \
   OUT_DIR=potato NUM_THREADS=300 MAX_MEM_GB=3000
```

2.1.3 Mate-pair scaffolding

The raw reads were processed using NextClip which filters out paired-end reads (reads which do not include the Nextera adaptor), and removes the adaptor from the mate-pair reads. We used NextClip version 1.3 which depends on R version 2.15.2, bwa vesrion 0.6.2 and texlive version 1.2.2013. We used the following command for NextClip:

```
nextclip -d -m 30 -t 0 -n 600000000 -l output.log \
   -i mp_R1.fastq -j mp_R2.fastq -o nextclip_potato
```

The number of reads before processing was 44 864 446, which were divided into four categories based on whether the Nextera adaptor was found in the reads or not. The reads with adaptor were further filtered for sequences that were long enough (less than 25 bp), and total of 33 044 388 (73.65 %) were deemed usable. Total 11 173 807 647 (15.4×) was written for a file to be used in the scaffolding stage.

The scaffolding was done with SOAPdenovo version 2.4 (r240) with the following commands and the configuration file given in Supplemental File **S2**:

```
finalFusion -D -K 71 -s soap-discovar.config -c assembly.fasta -g scaffolds
SOAPdenovo-127mer map -s soap-discovar.config -g scaffolds
SOAPdenovo-127mer scaff -g scaffolds
```

2.2 PacBio

Python version 2.7.9 was used for running the PacBio software.

2.2.1 Falcon

We used Falcon version 0.3.0 and the following command line:

```
fc_run.py fc_run_ver.cfg
```

The configuration file is in `fc_run_ver.cfg` (Supplemental File **S6**) which needs the list of input files `input.fofn` (Supplemental File **S3**).

2.2.2 Canu

We used Canu version 1.0 with the following command line:

```
canu -d run2-18.2.16/ \
   -p verrucosum \
   errorRate=0.06 \
   genomeSize=670m \
   -pacbio-raw filtered_subreads.fastq \
   useGrid=false
```

We initially used filtered subreads from the standard PacBio pipeline and then did a second assembly using subreads from the HGAP 3 pipeline (the filtered reads are the result of running the HGAP pipeline, below, up to the `P_PreAssemblerDagcon` stage).

2.2.3 HGAP

We used HGAP 3 as part of smrtanalysis 2.3.0p5 and the following command line:

```
smrtpipe.py --params=params_v1.xml \
   xml:input.xml > smrtpipe.log
```

`params_v1.xml` (Supplemental File **S5**) contains the configuration for HGAP and `input.xml` (Supplemental File **S4**) lists the raw PacBio input files.

2.3 10x Genomics Supernova

To assemble the 10x Genomics library we used Supernova version 1.1.1 and the following commands:

```
supernova run --id=verrusocum5 \
   --fastqs=potato/10x/supernova/fastq \
   --sample=potato
supernova mkoutput --asmdir=genome-assembly/10x/verrusocum5/outs/assembly \
   --outprefix=pseudohap_ver_500 \
   --style=pseudohap --minsize=500
```

The manufacturer recommends the use of 150 bp paired-end reads with a coverage of between 38$\times$ to 56$\times$. Since our library was higher coverage, and with 250 bp reads, we generated subsamples of the read sets to simulate the use of 150 bp reads and coverages of 48$\times$ and 52$\times$. The reads for each subsample were selected using a pseudorandom number generator with a known seed. Three different seeds were produced for each subsample to provide triplicates for testing. We found that in each case the subsampled libraries with shorter read lengths produced assemblies with significantly less contiguity. Our original assembly has an N50 of 2.38 Mbp, while the 52$\times$ version has 2.15 Mbp and the 48$\times$ version has 1.94 Mbp.

The subsample program can be found here:
`https://github.com/georgek/bio-tools/blob/master/fastq-subsample.cc`.

2.4 BioNano

We used BioNano Irys version 2.0 (`http://bionanogenomics.com/support/software-downloads/`), Python version 2.7.12 as part of Anaconda version 2.5.0, and Perl version 5.16.3.

2.4.1 Optical map assembly

First we built a BioNano *de novo* optical map assembly using the following command line:

```
python bionano/scripts_UV2K/pipelineCL.py \
   -U -d -T 32 -j 16 -N 4 -i 5 -w \
   -a potato_arguments_v1.xml \
   -t bionano/tools \
   -l output \
   -b Molecules.bnx \
   -C clusterArguments.xml
```

XML configuration is given in Supplemental Files **S7** and **S8**.

2.4.2 Scaffolding

Scaffolding is performed using the optical map assembly and one of our *S. verrucosum* genome assemblies.

```
perl bionano/scripts_SLURM/HybridScaffold/hybridScaffold.pl \
   -n supernova.fasta \
   -b EXP_REFINEFINAL1.cmap \
   -c potato_hybrid_scaffold_parameters.xml \
   -o output \
   -B 1 -N 1 \
   -r bionano/tools/RefAligner
```

`EXP_REFINEFINAL1.cmap` is the optical map assembly from the previous section. `supernova.fasta` is our Supernova assembly (see Section 4.4). `potato_hybrid_scaffold_parameters.xml` is the configuration file (Supplemental File **S9**).

2.5 Dovetail

Dovetail scaffolding was performed for the `discovar-mp` and `falcon` assemblies by Dovetail Ltd. using HiRise.

2.6 BAC assembly

2.6.1 Illumina

The BAC reads were filtered to remove any phiX, *E. coli*, and the pIndigo5 BAC vector sequence. The filtered reads were then assembled using DISCOVAR *de novo* and the assembled contigs were filtered to remove any residual pieces of BAC vector and contigs under 500 bp in length.

2.6.2 PacBio

The BAC pools were assembled with HGAP as part of smrtanlysis 2.3.0p5 with standard settings.

Assembly	Contigs	N50 (Mbp)	Max length (Mbp)	Total length (Mbp)	%N
abyss77	569 872	0.078	0.501	759.2	6.65
abyss77-mp	99 999	0.487	2.595	629.7	1.96
abyss113	170 623	0.324	2.258	740.9	1.47
abyss113-mp	99 999	0.379	2.258	634.4	1.72
discovar	99 999	0.076	0.497	679.3	0.06
discovar-mp	99 999	0.858	4.132	666.4	2.91
discovar-mp-dt	99 999	4.713	14.050	685.2	2.90
discovar-mp-dt-bn	99 999	5.285	12.470	680.6	2.94
canu	8 138	0.290	4.701	722.3	0.00
canu-bn	7 840	0.404	4.701	764.1	5.47
hhgap	5 571	0.585	4.876	715.9	0.00
hgap-mp	4 814	1.021	7.078	716.5	0.09
hgap-bn	5 164	1.087	8.633	749.8	4.52
falcon	2 461	0.712	5.738	659.3	0.00
falcon-dt	1 377	2.553	16.490	659.4	0.02
falcon-dt-bn	1 331	2.868	16.490	662.3	0.45
falcon-bn	2 343	0.887	5.738	667.1	1.17
10x	22 492	2.238	9.908	688.7	6.81
10x-bn	22 476	2.327	9.908	690.4	7.04

Table S3.1: Comprehensive contiguity information for all assemblies

3 Evaluation

All of the code used to perform the evaluation is available on GitHub in the georgek/potato-figures repository. The complete process from raw data to figures can be found at `https://georgek.github.io/potato-figures/figures.html`.

3.1 Contiguity

Table S3.1 shows a comprehensive table of contiguity measures for the various assemblies.

3.2 K-mer content

In Section we used KAT to see the *k*-mer content of each assembly. Since we were comparing the assemblies to the Illumina reads, we polished the Falcon assembly using Pilon to reduce the difference due to the sequencing platform. Figure S3.1 shows the KAT plots for Falcon with and without the polishing step for comparison.

3.3 Synteny

The complete set of mummer plots showing alignments to the *S. tuberosum* reference v4.04 for the Falcon assembly, DISCOVAR assembly, and Supernova assembly are shown in Figures S3.2, S3.3, and S3.4, respectively.

3.4 Gene content

3.5 BUSCO

BUSCO was run for each assembly using the following command (in this case for `discovar-mp-dt-bn`):

```
python BUSCO.py -i discovar-mp-dt-bn.fasta -o discovar_plant \
   -l embryophyta_odb9 -m genome -c 16 -sp arabidopsis
```

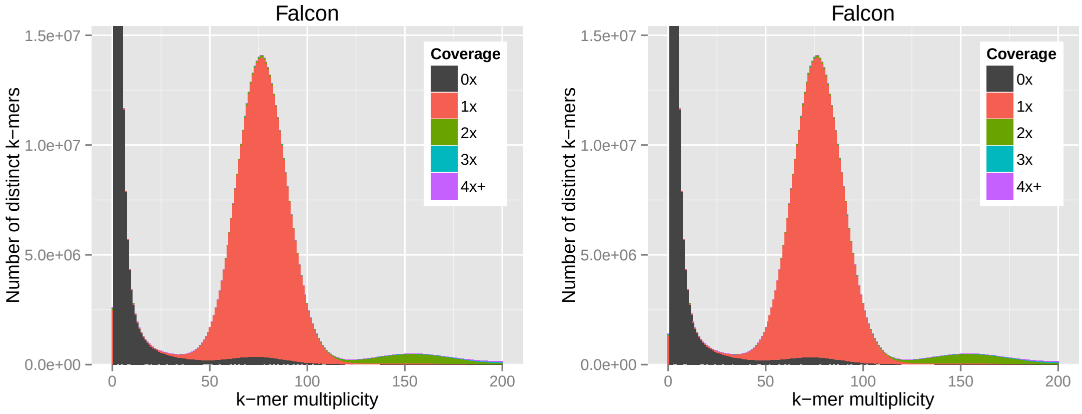

(a) Unpolished. **(b)** Polished.

Figure S3.1: KAT plots for Falcon assembly with and without polishing using Pilon.

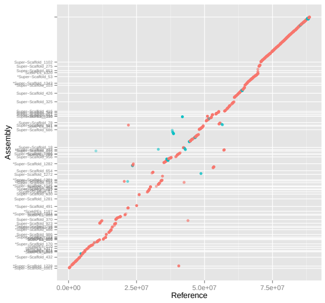

(a) Chromosome 1.

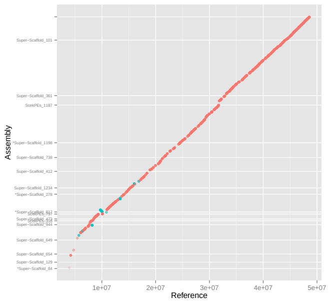

(b) Chromosome 2.

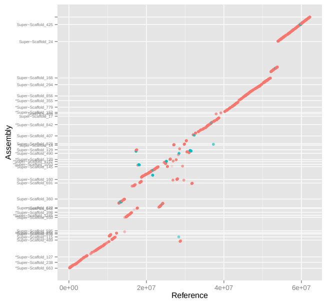

(c) Chromosome 3.

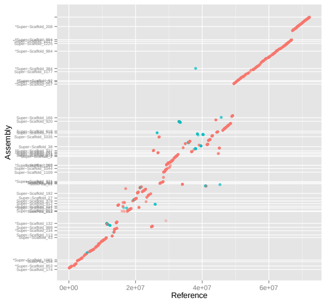

(d) Chromosome 4.

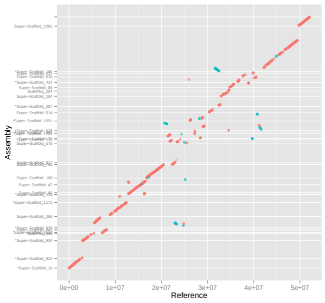

(e) Chromosome 5.

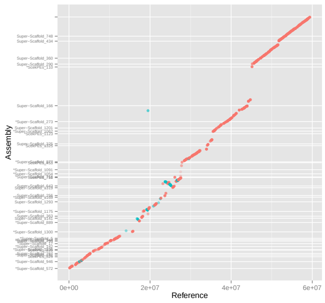

(f) Chromosome 6.

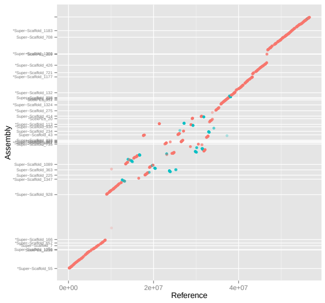

(g) Chromosome 7.

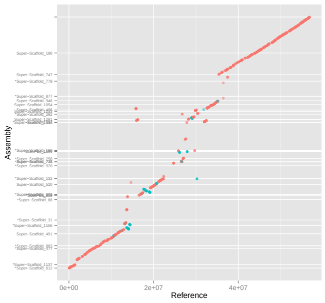

(h) Chromosome 8.

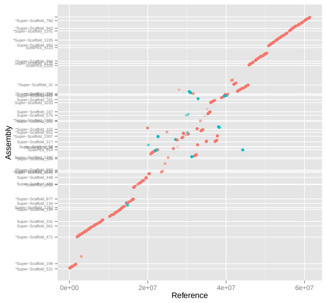

(i) Chromosome 9.

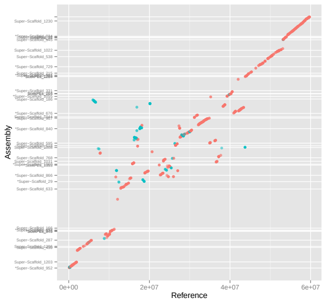

(j) Chromosome 10.

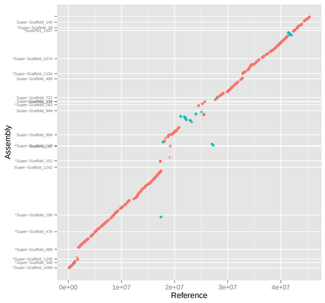

(k) Chromosome 11.

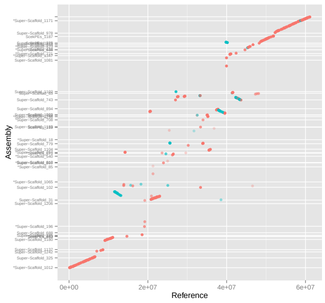

(l) Chromosome 12.

Figure S3.2: Mummer plots for Falcon assembly against the *S. tuberosum* reference.

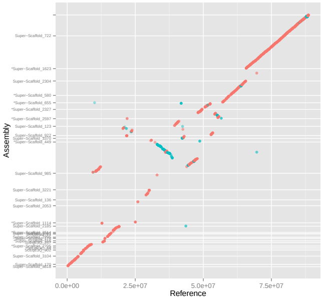

(a) Chromosome 1.

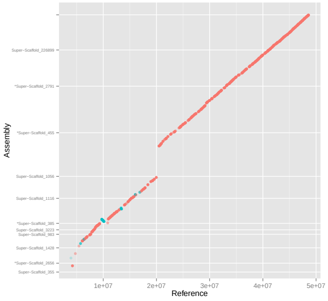

(b) Chromosome 2.

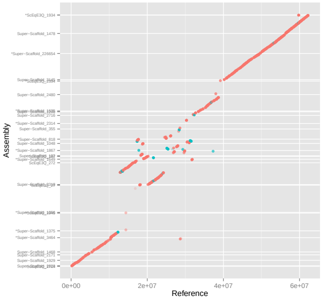

(c) Chromosome 3.

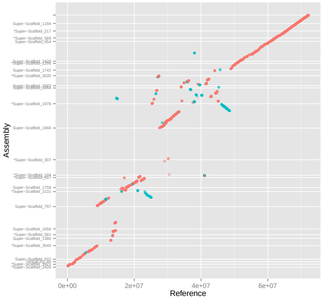

(d) Chromosome 4.

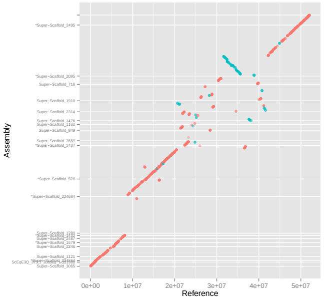

(e) Chromosome 5.

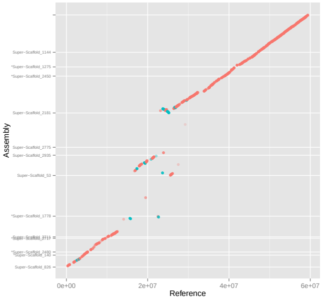

(f) Chromosome 6.

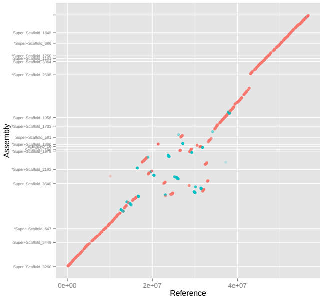

(g) Chromosome 7.

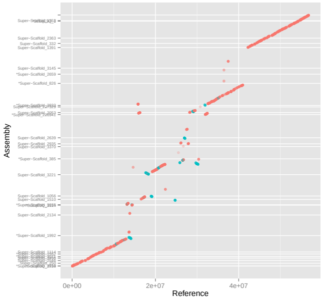

(h) Chromosome 8.

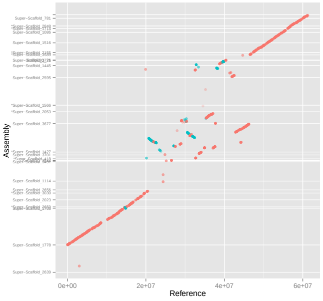

(i) Chromosome 9.

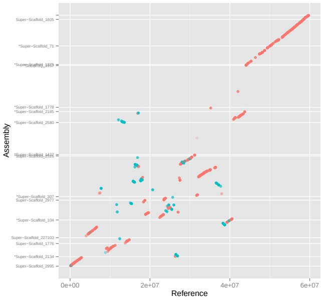

(j) Chromosome 10.

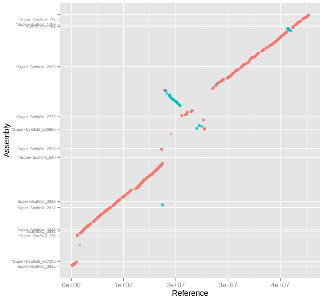

(k) Chromosome 11.

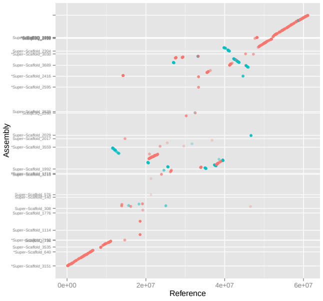

(l) Chromosome 12.

Figure S3.3: Mummer plots for Discovar assembly against the *S. tuberosum* reference.

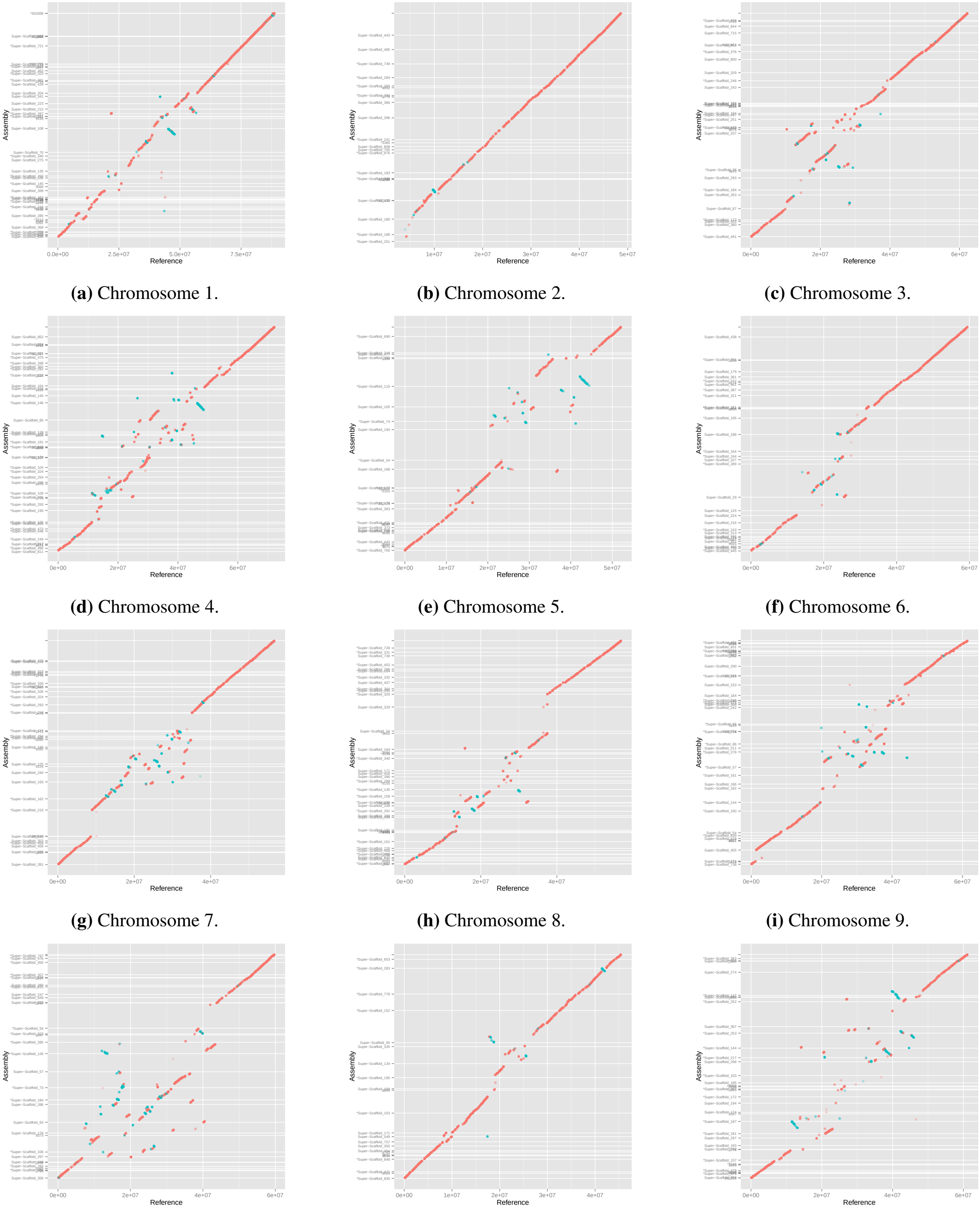

(a) Chromosome 1. **(b)** Chromosome 2. **(c)** Chromosome 3.

(d) Chromosome 4. **(e)** Chromosome 5. **(f)** Chromosome 6.

(g) Chromosome 7. **(h)** Chromosome 8. **(i)** Chromosome 9.

(j) Chromosome 10. **(k)** Chromosome 11. **(l)** Chromosome 12.

Figure S3.4: Mummer plots for Supernova assembly against the *S. tuberosum* reference.

4 Data availability

All read data is available in the European Nucleotide Archive (ENA) under the project PRJEB20860. The following subsections give the individual accession numbers for runs or analyses.

4.1 Short reads

DISCOVAR reads: ERR1988833. Dovetail reads: ERR1988846. Mate-pair reads: ERR1988848, ERR1988849. TALL reads: ERR1988850, ERR1988851. 10x reads: ERR1990231, ERR1990232, ERR1990233, ERR1990234.

4.2 Long reads

PacBio reads were generated in the following runs, each containing one or more SMRT cell: ERR1988882, ERR1988883, ERR1988884, ERR1988885, ERR1988886, ERR1988887, ERR1988888, ERR1989452, ERR1989453, ERR1989454, ERR1989455, ERR1989456, ERR1989457.

4.3 Optical map

The BioNano optical map is available in analysis ERZ438893.

4.4 Assemblies

- The Illumina BAC assemblies are in:

Accession	Assembly	Accession	Assembly
PRJEB21134	BAC 13	PRJEB21144	BAC 45
PRJEB21135	BAC 14	PRJEB21145	BAC 47
PRJEB21136	BAC 21	PRJEB21146	BAC 49
PRJEB21137	BAC 22	PRJEB21147	BAC 53
PRJEB21138	BAC 23	PRJEB21148	BAC 63
PRJEB21139	BAC 28	PRJEB21149	BAC 66
PRJEB21140	BAC 34	PRJEB21150	BAC 71
PRJEB21141	BAC 41	PRJEB21151	BAC 74
PRJEB21142	BAC 42	PRJEB21152	BAC 84
PRJEB21143	BAC 43	PRJEB21153	BAC 93

- The PacBio assembly of BAC 22 is in PRJEB21154;
- The whole genome assemblies are in:

Accession	Assembly	Accession	Assembly
PRJEB21112	10x-asm	PRJEB21122	falcon-asm
PRJEB21113	abyss113-asm	PRJEB21123	hgap-asm
PRJEB21114	abyss113-mp-asm	PRJEB21124	hgap-mp-asm
PRJEB21115	abyss77-asm	PRJEB21125	10x-bn-asm
PRJEB21116	abyss77-mp-asm	PRJEB21126	canu-bn-asm
PRJEB21117	canu-asm	PRJEB21127	discovar-mp-dt-bn-asm
PRJEB21118	discovar-contig-asm	PRJEB21128	falcon-bn-asm
PRJEB21119	discovar-mp-dt-asm	PRJEB21129	falcon-dt-bn-asm
PRJEB21120	discovar-mp-asm	PRJEB21130	hgap-bn-asm
PRJEB21121	falcon-dt-asm		

5 File list

S1 `pair_end_potato.xml`. Input file for RAMPART used to automate ABySS assemblies,

S2 `soap-discovar.config`. Configuration file for SOAPdenovo.

S3 `input.fofn`. Raw input for PacBio assembly.

S4 `input.xml`. Raw input for PacBio assembly in XML format.

S5 `params_v1.xml`. Configuration for HGAP assembler.

S6 `fc_run_ver.cfg`. Configuration for Falcon assembler.

S7 `clusterArguments.xml`. Cluster arguments for BioNano *de novo* optical map assembly.

S8 `potato_arguments_v1.xml`. Configuration for BioNano optical map assembly for *S. verrucosum*.

S9 `potato_hybrid_scaffold_parameters.xml`. Configuration file for BioNano scaffolding.
