# Supplementary material for: A critical comparison of technologies for a plant genome sequencing project
Source: Gigascience. 2019 Jan 9;8(3):giy163. doi: 10.1093/gigascience/giy163 (PMC6423373; doi:10.1093/gigascience/giy163)
Supplement: Response_to_Reviewer_Comments_Original_Submission.pdf [file giy163_response_to_reviewer_comments_original_submission.pdf]

Author's Response To Reviewer Comments

Close

I'd like to thank the reviewers for the time they took to carefully read our manuscript, their helpful comments, and positive feedback. When we started plant genome sequencing projects we looked for manuscripts comparing the different methods, but couldn't find one that focused on plant genomes. Having conducted this study we tried to document it so that others could use our findings. We hope our study and datasets would be of use to the research community: both to plant genetics and assembly experts, and are happy to hear that the reviewers find it has merit.

Best Wishes,

Matt

Responses to reviewers:

Reviewer #1: This paper is a comparison of many different methods of sequencing and assembly to get the best result for a plant genome that has its own specificities and difficulties compared to other eukaryotes. The paper focusses on standard and new technologies. The paper takes into account the prices, the compute time and what biological material is needed, which is a good point for this type of method comparison paper. It helps other people to be aware of all the aspects of a sequencing and assembling project.

1. In the Results section, the first sentence announces that the study will be presented in two parts: one comparing short vs. long reads, and the second comparing longer-range scaffolding technologies. I found it not clearly explained. I was expecting the second part to be short reads based assembly plus method A or B or C of long-range scaffolding then long-reads based assembly plus method A or B or C of long-range scaffolding. In fact, it is one short-reads assembly combined with one or many long-range scaffolding techniques compared also to long-reads assembly combined with one or many long-range scaffolding techniques. I suggest to make your message clearer.

We have rewritten the first paragraph. It now reads:

The results of this study are presented in two parts. In the first part we compare several short read (Illumina) to long read (PacBio) based assemblies. These represent the
simplest type of sequencing projects that are often undertaken. We then choose one each of the Illumina based and one PacBio based assemblies and in the second part we use various different combinations of longer-range scaffolding data from newer technologies, namely in vitro Hi-C (Dovetail) and optical mapping (BioNano Genomics) to increase continuity. Finally we compare these approaches to the read cloud(10x Genomics Chromium) technology, which promises short read assembly and longer-range scaffolding simultaneously. Validating the assemblies for sequence and scaffolding accuracy we find strengths and weaknesses, and that methods

differ hugely in their DNA, time, computational requirements and cost.

2. In the 'Contig assembly and scaffolding' part:
- For the TALL library, which sequencing machine was used, is it also on a HiSeq run? And for the Discovar assembly you do not give the genome size estimate?

We have changed the text to “sequenced with 100bp and 150bp paired-end reads on two Illumina HiSeq 2500 runs.” which gives the details the reviewer asked for. Also for the genome size estimates, we have now provided the preqc estimates for both DISCOVAR and TALL libraries:

“We analysed the TALL library reads with preqc, part of the SGA assembler (Simpson et al. 2012), which gives a genome size estimate of 702Mbp, while the same analysis on the DISCOVAR library yielded 722Mbp. The latter agrees better with the 727Mbp size of the potato genome assembly (The Potato Genome Sequencing Consortium 2011)”

3. Please explain why you used two different assembly algorithms?

We have added a sentence:
“Discovar de novo requires a specific data type (250bp paired reads, from a PCR-free libray with an insert size distribution around 500bp). Thus we could not use Discovar for the TALL library data, instead another leading short read assembler, AbySS, was used as it is well suited for the TALL data type.”

4. You said that the two assemblies you get (TALL and Discovar) are more contiguous than the equivalent of S. tuberosum genome, and cite a paper. Maybe, include the statistics of that paper so it is easier for the reader to compare.

We have continued the sentence
“, where the reported contig N50 from pair end reads is 22.4kbp.”

4. Considering the coverage of the two libraries are different, have you tried to normalize the results so it is more comparable? Maybe you should consider a k-mers analysis to be sure the assemblies you get are representative of the raw reads.

We did the preqc analysis for both libraries giving similar genome size estimates, see the earlier comment. However, the sequencing lengths were different and also the sequencing runs, as it is difficult to have perfect control over all the data. We did provide KAT (kmer) analysis in the paper which shows that the assemblies are representative of the raw Illumina reads. In a large experimental dataset such as this, pairwise comparisons can quickly spiral upwards, hence we also supply extensive documentation of how to generate these plots for the interested reader.

5. In the 'PacBio assembly" section:
- You said that canu and hgap assemblies contain more than all other assemblies. Please specify

what you mean by more content. Based on Table 1, I cannot agree it contain more contigs than all other assemblies, and their N50 value, Max length and Total length are not so much higher compare to other assemblies (example for N50, better value in falcon, for Max length better value in falcon, for total length similar value in abyss and abyss+mp).

We have changed this to:
The canu and hgap assemblies contain slightly more sequence content (as measured by the total length of the assembly), and also a lower percentage of unknown bases (measured by N base %) than the short read assemblies. This may be due to their capturing of additional difficult sequences, especially repeat elements which short read assemblies are known to have problems traversing.

6. Why producing alternate contig should be an argument in favour to keep the falcon assembly? With it you keep track of 'more' information but, do you use these alternate contig in the end? The choice of keeping falcon is not so much explained. Why do you think it is the best performing choice to do the downstream analysis?

We have changed this to:

Falcon also produced 9.9Mbp of alternate contigs, likely from residual heterozygosity, which will be useful for interpreting downstream genetic results e.g. forward and reverse genetic screens. We also found this assembly was easier and faster to run thanHGAP3, and the basepair accuracy of canu read correction to be lower than HGAP3 read correction. For these reasons we chose the falcon assembly (minus the alternative contigs) to take forward to hybrid scaffolding.

7. In the 'Longer-range scaffolding part:
- On the 'Dovetail' section, you said that discovar+mp assembly improves from 825kbp to 4700kbp when become discovar+mp+dt but in Table 1 it is written that dicovar+mp has N50=858kbp, please check the value. Also forfalcon, on Table 1 it is written 712 kpb and on this section, you say 710kbp, please check.

We’ve checked this. For simplicity in the Table 1, we consider only contigs that are longer than 1kb, as explained in the legend. For the Dovetail section, we use the N50 all contigs and scaffolds that abyss-fac, part of Abyss 1.9.0 reports.

We have updated this to read
“Dovetail used their HiRise software to further scaffold the discovar-mp assembly, increasing the N50 from 860kbp to 4713kbp, and the falcon assembly, increasing the N50 from 712kbp to 2553kbp. These assemblies are called discovar-mp-dt and falcon-dt, respectively.”

8. On the '10x Genomics' section:
- You explain why you used 10X alone to perform the supernova assembly but why havn't you also combined the 10X data to the Discovar and Falcon assemblies as for Bionano or Dovetail?

We did not use the 10x data to superscaffold the Discovar or Falcon assemblies because the tools for this were not available at the time, and because the biggest attraction is for this method is that it can use just a single data type to yield megabase scaffolds.

9. You said that the trimmed reads generated "very similar results", but similar to what exactly?

We have continued this sentence, but adding “compared to the ones reported above.” We found that trimming of the 250PE reads to recommended 150 PE reads did not change any assembly statistics and neither did the subsampling to the recommended coverage. It seems that the assembler itself may perform such data curation steps if necessary.

10. On the 'Assembly evaluation' part:
- On figure 2 what means "KAT"?. And the blue and purple colour are not visible, so it is difficult to evaluate what you say.

The caption now starts: “k-mer spectra plots from the k-mer Analysis Toolkit (KAT) comparing three S. verrucosum contig assemblies.” Colours have been adjusted.

11. In the 'gene content' section:
- Based on what figure 4 show, I would not say one is better than the other, but all give similar results in terms of gene content. The differences are really small. Not sure it is "significant".

We agree this is not a significant difference, but we felt we should comment upon it as genes are so important for the users of genome assemblies. Many of us are aware of complaints by users when one of their favourite genes is missing from an assembly.

Because it is a small difference have removed the sentence:

“the discovar-mp-dt-bn assembly is the most complete while supernova-bn is the worst performing.”

and replaced it by

“We found that each of the three assemblies shows at least 95% of Buscos as complete, with just a small difference of only 2-3% missing. “

12. In the 'Discussion' part:
- You suggest that MinION can be a good technology to overcome the repetitive regions, what about the error rate compare to what is available with PacBio for example?

We have added a reference a recent JXB review on the use of nanopore for plant research (https://doi.org/10.1093/jxb/erx289). In brief currently pacbio is 85% accurate and nanopore is 92-95% (depending on chemistry) accurate, we agree that both would struggle to separate

recent repeat copies. However, long enough reads could span a repeat with unique sequence anchors either side and so recover the repeats. Obviously in the world of long reads, size matters and current PacBio read N50s are ~15kb, whereas Nanopore datasets have been described with read N50 as high as 99.7kb with many labs getting >50kb. Hence the interest in nanopore reads. However, there is still a reluctance in many genome projects to select nanopore because it is still an evolving platform. This leads to a lack of reliability e.g. predictable flowcell yields, which makes it hard to integrate into many plans and budget accordingly.

We have added the paragraph:

"Recently ultra-long reads with an N50 of 99.7kbp (max. 882kbp) with ~ 92% accuracy have been produced with the MinION R9.4 chemistry using high molecular weight DNA from a human sample (Jain et al. 2018}. If this is also achievable on plant material the remaining (mostly repetitive) fraction of genomes should become visible. An earlier S. penellii Nanopore assembly (Schmidt et al. 2017) reported average read length of 12.7kbp} and error rate of 18-20%."

13. You suggest two different versions of HiRise may have been used. Could you check to be sure if it the case or not? If it is, what's the differences between the two versions you used? Could it influence the results you get?

We have contacted Dovetail and their answer confirmed there are the two versions of HiRise. Because it is a proprietary system the company is reluctant to describe the differences in detail, but we have given the version numbers. Based on this, we have added the following text.

"The two Dovetail scaffolding processes shared the same Hi-C sequence data but were conducted many months apart (discovar-mp first and later falcon}), and used different versions of Dovetail's proprietary HiRise software, versions 0.9.6 and 1.3.0, respectively, which may have affected the results."

Reviewer #2: In "A critical comparison of technologies for a plant genome sequencing project", Paajanen et al. describe a rigorous experiment that is often discussed but rarely published in this way. To be frank, I have never read a manuscript that was so detailed in exactly the way programs were run, as shown in the supplemental and github code. The manuscript is a pleasure to read and digest, and I have very few comments at all to improve it.

Thank you very much for your kind comments.

1. In the local accuracy section, was the Bionano data able to accurately assess the gap size? If so I would highlight that, as it is in contrast to what Dovetail can accomplish.

First we see that BioNano clearly adds more Ns into the assembly, by comparing the two Falcon assemblies scaffolded with either BioNano or Dovetail. As the Falcon assembly did not have any N's to start with, so this is an easy comparison to do. This comparison revealed that

BioNano is trying to calculate the gap sizes, which also leads to the Falcon+BioNano assembly being 7.7 Mbp longer than the Falcon+Dovetail. Thus BioNano estimates the gap sizes whereas Dovetail just marks them with an arbitrary 100 N bases.

We have added the sentence at the end of the paragraph.
"While BioNano software estimates gap sizes, we note that BioNano data was not able to close this particular gap in any of the assemblies."

2. The 2nd paragraph of the results section "The quality and quantity of DNA…" is out of place and does not flow as a result.

We moved the first part of this paragraph to the beginning of the discussion, which now reads:

The quality and quantity of DNA available, whether it is from fresh or frozen tissue, and ease of its extraction will often dictate which preparation and sequencing technologies are feasible to use. Budget constraints do play a large part in the choice of technologies to be adopted for any genome project. Assembly and scaffolding methods are often effectively the choice of sequencing method, but the properties of the genome will also affect the results. Interestingly, none of the assembly approaches we used lead to a "bad assembly" e.g. one that fails to assemble large parts of the genome or makes many systematic errors (as seen in many early short read assemblies). This speaks to the tremendous progress made in improved sequencing technologies and assembly algorithms. Instead they differ mostly in the length of the ungapped sequence and scaffolds, with much smaller differences in missing sequence and gene content, duplicated regions, and per base accuracy.

3. I would mention minimap/miniasm as low-computational power alternatives to the pacbio/nanopore assemblers, with the caveat that there is no error correction. This manuscript is one-half "state of the field" paper, one-half data, so readers from all backgrounds would appreciate it. Other than that, most popular plant genome assemblers were covered in the manuscript.

We added miniasm in the section about PacBio assemblies in the following text:

Another long read assembler, that we chose not to use, because it does not include any error correction is miniasm \cite{miniasm2016}. This is a fast lower computational power alternative to the ones that we used in this paper and is useful for many purposes e.g. empirical testing of long read assemblies.

4. Similarly I would also briefly mention FALCON-Phase and Trio Binning as newer approaches to handling Pacbio/Hi-C data for true diploid assembly.

We have added this sentence to the end of the discussion:

“Newer methods have recently been developed to assemble diploid genomes into chromosome scale phase blocks \cite{Kronenberg327064} or even to exploit the haplotype diversity using a ``trio binning" approach developed in \cite{Koren271486}, so we expect to see more true diploid assemblies in the near future.”

5. P8L12. The MinION long reads keep getting longer. With BulkVis (https://www.biorxiv.org/content/early/2018/05/03/312256) the longest published read is now 2.2 megabases.

We have added the reference to the preprint, we note that the dataset is the same as in Jain et al., which was already referenced.

6. The discussion ended rather abruptly with data rather than a final wrap-up. Perhaps the manuscript could end with a small paragraph about how this approach worked with this genome, but is subject to variation depending on genome size, heterozygosity, repeat content, polyploidy etc? The fact that genome assembly is not "one size fits all" might fit the overall theme of the manuscript.

Good point, we certainly don’t believe that one recipe will work for all genomes. We have added a final paragraph as suggested:

“Even though we found some surprisingly small differences between assemblies of S. verrucosum, this is an inbred diploid potato species, with a medium size genome and is in no way exceptional. As there are ~300,000 angiosperms alone [51] we remind the reader, that many factors e.g. genome size, the ease of high quality HMW DNA extraction, the types of repeat content, polyploidy or heterozygosity may pose additional hurdles affecting the choice of technology and how well they will perform. Heterozygosity, in particular, complicates the assembly process and if individual haplotypes are desired this places limitations on which strategies can be used. The careful choice of sample where possible, such as a highly inbred plant or doubled haploid, can remove or minimise these problems. This approach was also adopted for the potato DM reference, whereby a completely homozygous ``doubled monoploid" was used as the heterozygous diploid RH genotype originally selected
for sequencing proved difficult to assemble due to the extremely high level of heterozygosity.”

Reviewer #3: In this study, the authors compared assembly qualities and cost by using multiple sequence data of S. verrucosum (Illumina, PacBio, Dovetai, Chromium, Bionao) and combination. The manuscript is well written and the results are useful and informative for the scientist who are at a loss to select the best sequencing platform for de novo assembly.

Thank you very much for your kind comments.

1. The assembly result with Illumina and Pacbio reads are summarized in Table 1. However, those with Longer-range scaffolds were described in text only and difficult to understand the

differences. Could you make a table summarizing all the assembly results (Number of assembled sequences, N50, Max length, total length and N%)? It would help the understanding of readers.

We conducted a lot of different assemblies, in writing the paper we tried to simplify while retaining the main points. For the interested reader, we have added an extended summary (table S3.1) to the supplementary data and refer to it in the main manuscript. This supplementary table was produced using abyss-fac as part of Abyss 1.9.0 which takes into account all contigs, hence the slightly different numbers from the Table 1 in the main text that reports only contigs longer than 1 kb.

2. Introduction
P2, L60 (left): Describe estimated genome size of S. verrucosum

We have explain how this was carried out.

“In this paper we compare several practical de novo assembly projects
of a Mexican wild potato species Solanum verrucosum. We chose this
genome because Solanum verrucosum is a self-compatible, diploid,
tuber-bearing, wild potato species which we inbred further to produce the line
Ver-54. The estimated genome size based on k-mer content is 722Mbp.”

3. Result
P3, L23 (right): Discover and abyss are remarkably similar.
Really? Total length in discover is 8% shorter than abyss, and I think it should not be ignored.

True there are some differences but we expected larger ones. We have changed the sentence to:
“The results for these two Illumina assemblies are similar in contiguity and shown in Table1. However, while ABySS assembled ~8\% longer total length, the number of small contigs was larger leading to very similar contig N50 to Discovar. One additional feature was that AbySS performed more scaffolding using the paired end data but did not fill many of the introduced gaps leading to ~100x higher % of N bases than Discovar.”

4. P3, L33 (right): The total coverage of the LMP library was 15X.
Describe the ratio of PCR duplicates in the sequences.

We have changed the sentence:
“The total coverage of the LMP library was 15x after we had filtered out duplicates (23.4% of reads), reads that did not contain a Nextera adapter or were too short to be useful.”

5. P3, L59 (right): Falcon has closet to the estimated genome size.
The genome size was estimated as 722 Mb based on Tall library reads. It seems canu is closest.

Have changed this sentence to : “The canu and hgap assemblies contain considerably more content than all other assemblies. The falcon assembly has the highest N50, and while canu is closest to the kmer estimated genome length.”

6. Assembly evaluation, kmer content.
The approach in here is appropriate, however, it is difficult to understand the differences from Fig 2. The authors describe the potential duplicate content assemblies as 0.15-1.3? Are the numbers calculate based on area of 2X? If so, showing the area ratio to the whole are in table is more easy to understand the differences of the assembled quality. Please re-consider the style of figure/Table in this section.

It’s true these plots are rich in data but unfamiliar readers need to be walked through them. K-mer plots are increasingly familiar to the assembly community, including from KAT due to use by ourselves and others e.g. in Bioinformatics (Mapleson et al. 2016), Genome Research (Clavijo et al. 2017) and Gigascience (Zimin et al. 2017). We’ve rewritten the text describing and discussing this figure to make it clearer to the reader.

7. P5 L5(right)The small red bar on the origin: They are really small and cannot see. Please change the layout.

We have thickened the bar at the origin in PDF or online this figure can be zoomed into as well.

8. Gene content:
P5, L33 (right) We align the S. tuberosum representative transcript sequences..
Did authors used transcript sequences registered SRA? Describe the source of the sequences

This was from the latest assembly update from the SpudDB website at:
http://solanaceae.plantbiology.msu.edu/pgsc_download.shtml

We amended the text to:
“The S. tuberosum representative transcripts (PGSC_DM_V403_representative_genes - http://solanaceae.plantbiology.msu.edu/pgsc_download.shtml) from were aligned to the assemblies using Blast and the coverage of transcripts at various thresholds calculated using a tool we developed.”

9. Library accuracy
P6, L61 (left) Dovetail data shows much smoother fragment distribution Add the dovetail reads distribution in Fig.3.

We have not been able to add this to the Figure 3, as this is already a very busy figure, and already contains the Dovetail (and other data types) plotted on the exemplar region. We have made all the data and assemblies available so that any interested reader can visualise and compare the PE, LMP, Dovetail, 10x etc. insert size distributions.

10. Figure 6. I can see the letters of Y axis. Please change the font size. Please also specify the version of S. tuberosum reference in the legend.

We have removed the letters from the Y axis as we felt that those were not useful anyway. The

legend has been updated to contain “the S. tuberosum reference version 4.03.”

11. Table 2. Add the sequence coverage in the table.

Table 2 provides an overview of the requirements and costs of different approaches, so we feel that the sequencing coverage is not appropriate in this table. The sequence coverage for each library is provided in Table S1.1.

--

Close
