# Supplementary material for: A critical comparison of technologies for a plant genome sequencing project
Source: Gigascience. 2019 Jan 9;8(3):giy163. doi: 10.1093/gigascience/giy163 (PMC6423373; doi:10.1093/gigascience/giy163)
Supplement: Response_to_Reviewer_Comments_Revision_1.pdf [file giy163_response_to_reviewer_comments_revision_1.pdf]

Author's Response To Reviewer Comments

Close

Dear Scott,
we have made the minor revisions you requested (pasted below as an aide memoire).
My postdoc is applying for her first lecturer position at the moment so is keen to have this in print or online soon if thats possible.

Hope this finds you well and have a great christmas. Maybe see you at AGBT?

Matt

###########
We are now happy with your manuscript "A critical comparison of technologies for a plant genome sequencing project" (GIGA-D-18-00164R1). I am pleased to inform you that it is potentially acceptable for publication in GigaScience, once you have carried out some essential revisions to make it meet our reproducibility policies.

1. Can you change the Data Access section to Availability of Supporting Data and add an additional line to cite the GigaDB. This can be something similar to this:

Archival copy of the code, assemblies and other data are available in the GigaScience GigaDB repository[66].

Then cite the Data DOI in the references as reference 66:

Paajanen P; Kettleborough G; López-Girona E; Giolai M; Heavens D; Baker D; Lister A; Cugliandolo F; Wilde G; Hein I; Macaulay I; Bryan GJ; Clark MD (2018): Supporting data for "A critical comparison of technologies for a plant genome sequencing project" GigaScience Database. http://dx.doi.org/10.5524/100518

2. Can you add all relevant accessions and identifiers such as ORCID IDs (for authors that have them), accessions and RRIDs for the software (see: ftp://penguin.genomics.cn/pub/10.5524/RRID/RRIDlist.pdf), as well as version numbers for the software.

Once you have made the necessary corrections, please submit a revised manuscript online

Close
