# Supplementary material for: A critical comparison of technologies for a plant genome sequencing project
Source: Gigascience. 2019 Jan 9;8(3):giy163. doi: 10.1093/gigascience/giy163 (PMC6423373; doi:10.1093/gigascience/giy163)
Supplement: Reviewer_1_Report_Orginal_Submission -- CÃ©cile Monat — 6/13/2018 Reviewed [file giy163_reviewer_1_report_orginal_submission.pdf]

Reviewer Report

Title: A critical comparison of technologies for a plantgenome sequencing project

Version: Original Submission **Date:** 6/13/2018

Reviewer name: CÃ©cile Monat

Reviewer Comments to Author:

This paper is a comparison of many different methods of sequencing and assembly to get the best result for a plant genome that has its own specificities and difficulties compared to other eukaryotes. The paper focusses on standard and new technologies. The paper takes into account the prices, the compute time and what biological material is needed, which is a good point for this type of method comparison paper. It helps other people to be aware of all the aspects of a sequencing and assembling project.

In the Results section, the first sentence announces that the study will be presented in two parts: one comparing short vs. long reads, and the second comparing longer-range scaffolding technologies. I found it not clearly explained. I was expecting the second part to be short reads based assembly plus method A or B or C of long-range scaffolding then long-reads based assembly plus method A or B or C of long-range scaffolding. In fact, it is one short-reads assembly combined with one or many long-range scaffolding techniques compared also to long-reads assembly combined with one or many long-range scaffolding techniques. I suggest to make your message clearer.

In the 'Contig assembly and scaffolding' part:
- For the TALL library, which sequencing machine was used, is it also on a HiSeq run? And for the Discovar assembly you do not give the genome size estimate?
- Please explain why you used two different assembly algorithms?
- You said that the two assemblies you get (TALL and Discovar) are more contiguous than the equivalent of S. tuberosum genome, and cite a paper. Maybe, include the statistics of that paper so it is easier for the reader to compare.
- Considering the coverage of the two libraries are different, have you tried to normalize the results so it is more comparable? Maybe you should consider a k-mers analysis to be sure the assemblies you get are representative of the raw reads.

In the 'PacBio assembly" section:
- You said that canu and hgap assemblies contain more than all other assemblies. Please specify what you mean by more content. Based on Table 1, I cannot agree it contain more contigs than all other assemblies, and their N50 value, Max length and Total length are not so much higher compare to other assemblies (example for N50, better value in falcon, for Max length better value in falcon, for total length similar value in abyss and abyss+mp).
- Why producing alternate contig should be an argument in favour to keep the falcon assembly? With it you keep track of 'more' information but, do you use these alternate contig in the end? The choice of

keeping falcon is not so much explained. Why do you think it is the best performing choice to do the downstream analysis?

In the 'Longer-range scaffolding part:
- On the 'Dovetail' section, you said that discovar+mp assembly improves from 825kbp to 4700kbp when become discovar+mp+dt but in Table 1 it is written that dicovar+mp has N50=858kbp, please check the value. Also for falcon, on Table 1 it is written 712 kpb and on this section, you say 710kbp, please check.

On the '10x Genomics' section:
- You explain why you used 10X alone to perform the supernova assembly but why havn't you also combined the 10X data to the Discovar and Falcon assemblies as for Bionano or Dovetail?
- You said that the trimmed reads generated "very similar results", but similar to what exactly?

On the 'Assembly evaluation' part:
- On figure 2 what means "KAT"?. And the blue and purple colour are not visible, so it is difficult to evaluate what you say.

In the 'gene content' section:
- Based on what figure 4 show, I would not say one is better than the other, but all give similar results in terms of gene content. The differences are really small. Not sure it is "significant".

In the 'Discussion' part:
- You suggest that MinION can be a good technology to overcome the repetitive regions, what about the error rate compare to what is available with PacBio for example?
You suggest two different versions of HiRise may have been used. Could you check to be sure if it the case or not? If it is, what's the differences between the two versions you used? Could it influence the results you get?

Methods

Are the methods appropriate to the aims of the study, are they well described, and are necessary controls included? Choose an item.

Conclusions

Are the conclusions adequately supported by the data shown? Choose an item.

Reporting Standards

Does the manuscript adhere to the journal's guidelines on minimum standards of reporting? Choose an item.

Choose an item.

Statistics

Are you able to assess all statistics in the manuscript, including the appropriateness of statistical tests used? Choose an item.

Quality of Written English

Please indicate the quality of language in the manuscript: Choose an item.

Declaration of Competing Interests

Please complete a declaration of competing interests, considering the following questions:

- Have you in the past five years received reimbursements, fees, funding, or salary from an organisation that may in any way gain or lose financially from the publication of this manuscript, either now or in the future?
- Do you hold any stocks or shares in an organisation that may in any way gain or lose financially from the publication of this manuscript, either now or in the future?
- Do you hold or are you currently applying for any patents relating to the content of the manuscript?
- Have you received reimbursements, fees, funding, or salary from an organization that holds or has applied for patents relating to the content of the manuscript?
- Do you have any other financial competing interests?
- Do you have any non-financial competing interests in relation to this paper?

If you can answer no to all of the above, write 'I declare that I have no competing interests' below. If your reply is yes to any, please give details below.

I declare that I have no competing interests

I agree to the open peer review policy of the journal. I understand that my name will be included on my report to the authors and, if the manuscript is accepted for publication, my named report including any attachments I upload will be posted on the website along with the authors' responses. I agree for my report to be made available under an Open Access Creative Commons CC-BY license (http://creativecommons.org/licenses/by/4.0/). I understand that any comments which I do not wish to be included in my named report can be included as confidential comments to the editors, which will not be published.

I agree to the open peer review policy of the journal

To further support our reviewers, we have joined with Publons, where you can gain additional credit to further highlight your hard work (see: https://publons.com/journal/530/gigascience). On publication of this paper, your review will be automatically added to Publons, you can then choose whether or not to claim your Publons credit. I understand this statement.

Yes Choose an item.
