# Supplementary material for: A critical comparison of technologies for a plant genome sequencing project
Source: Gigascience. 2019 Jan 9;8(3):giy163. doi: 10.1093/gigascience/giy163 (PMC6423373; doi:10.1093/gigascience/giy163)
Supplement: Reviewer_2_Report_Orginal_Submission -- Alex Harkess — 6/19/2018 Reviewed [file giy163_reviewer_2_report_orginal_submission.pdf]

Reviewer Report

Title: A critical comparison of technologies for a plantgenome sequencing project

Version: Original Submission **Date:** 6/19/2018

Reviewer name: Alex Harkess

Reviewer Comments to Author:

In "A critical comparison of technologies for a plant genome sequencing project", Paajanen et al. describe a rigorous experiment that is often discussed but rarely published in this way. To be frank, I have never read a manuscript that was so detailed in exactly the way programs were run, as shown in the supplemental and github code. The manuscript is a pleasure to read and digest, and I have very few comments at all to improve it.

> In the local accuracy section, was the Bionano data able to accurately assess the gap size? If so I would highlight that, as it is in contrast to what Dovetail can accomplish.

> The 2nd paragraph of the results section "The quality and quantity of DNA…" is out of place and does not flow as a result.

> I would mention minimap/miniasm as low-computational power alternatives to the pacbio/nanopore assemblers, with the caveat that there is no error correction. This manuscript is one-half "state of the field" paper, one-half data, so readers from all backgrounds would appreciate it. Other than that, most popular plant genome assemblers were covered in the manuscript. Similarly I would also briefly mention FALCON-Phase and Trio Binning as newer approaches to handling Pacbio/Hi-C data for true diploid assembly.

> P8L12. The MinION long reads keep getting longer. With BulkVis (https://www.biorxiv.org/content/early/2018/05/03/312256) the longest published read is now 2.2 megabases.

> The discussion ended rather abruptly with data rather than a final wrap-up. Perhaps the manuscript could end with a small paragraph about how this approach worked with this genome, but is subject to variation depending on genome size, heterozygosity, repeat content, polyploidy etc? The fact that genome assembly is not "one size fits all" might fit the overall theme of the manuscript.

Methods

Are the methods appropriate to the aims of the study, are they well described, and are necessary controls included? Choose an item.

Conclusions

Are the conclusions adequately supported by the data shown? Choose an item.

Reporting Standards

Does the manuscript adhere to the journal's guidelines on minimum standards of reporting? Choose an item.

Choose an item.

Statistics

Are you able to assess all statistics in the manuscript, including the appropriateness of statistical tests used? Choose an item.

Quality of Written English

Please indicate the quality of language in the manuscript: Choose an item.

Declaration of Competing Interests

Please complete a declaration of competing interests, considering the following questions:

- Have you in the past five years received reimbursements, fees, funding, or salary from an organisation that may in any way gain or lose financially from the publication of this manuscript, either now or in the future?
- Do you hold any stocks or shares in an organisation that may in any way gain or lose financially from the publication of this manuscript, either now or in the future?
- Do you hold or are you currently applying for any patents relating to the content of the manuscript?
- Have you received reimbursements, fees, funding, or salary from an organization that holds or has applied for patents relating to the content of the manuscript?
- Do you have any other financial competing interests?
- Do you have any non-financial competing interests in relation to this paper?

If you can answer no to all of the above, write 'I declare that I have no competing interests' below. If your reply is yes to any, please give details below.

I declare that I have no competing interests.

I agree to the open peer review policy of the journal. I understand that my name will be included on my report to the authors and, if the manuscript is accepted for publication, my named report including any attachments I upload will be posted on the website along with the authors' responses. I agree for my report to be made available under an Open Access Creative Commons CC-BY license (http://creativecommons.org/licenses/by/4.0/). I understand that any comments which I do not wish to

be included in my named report can be included as confidential comments to the editors, which will not be published.

I agree to the open peer review policy of the journal

To further support our reviewers, we have joined with Publons, where you can gain additional credit to further highlight your hard work (see: https://publons.com/journal/530/gigascience). On publication of this paper, your review will be automatically added to Publons, you can then choose whether or not to claim your Publons credit. I understand this statement.

Yes Choose an item.
