# Supplementary material for: A critical comparison of technologies for a plant genome sequencing project
Source: Gigascience. 2019 Jan 9;8(3):giy163. doi: 10.1093/gigascience/giy163 (PMC6423373; doi:10.1093/gigascience/giy163)
Supplement: Reviewer_3_Report_Orginal_Submission -- Sachiko Isobe — 6/28/2018 Reviewed [file giy163_reviewer_3_report_orginal_submission.pdf]

Reviewer Report

Title: A critical comparison of technologies for a plantgenome sequencing project

Version: Original Submission **Date:** 6/28/2018

Reviewer name: Sachiko Isobe

Reviewer Comments to Author:

In this study, the authors compared assembly qualities and cost by using multiple sequence data of S. verrucosum (Illumina, PacBio, Dovetai, Chromium, Bionao) and combination. The manuscript is well written and the results are useful and informative for the scientist who are at a loss to select the best sequencing platform for de novo assembly.

The assembly result with Illumina and Pacbio reads are summarized in Table 1. However, those with Longer-range scaffolds were described in text only and difficult to understand the differences. Could you make a table summarizing all the assembly results (Number of assembled sequences, N50, Max length, total length and N%)? It would help the understanding of readers.

Introduction
P2, L60 (left): Describe estimated genome size of S. verrucosum

Result
P3, L23 (right): Discover and abyss are remarkably similar.
Really? Total length in discover is 8% shorter than abyss, and I think it should not be ignored.

P3, L33 (right): The total coverage of the LMP library was 15X.
Describe the ratio of PCR duplicates in the sequences.

P3, L59 (right): Falcon has closet to the estimated genome size.
The genome size was estimated as 722 Mb based on Tall library reads. It seems canu is closest.

Assembly evaluation, kmer content.
The approach in here is appropriate, however, it is difficult to understand the differences from Fig 2. The authors describe the potential duplicate content assemblies as 0.15-1.3? Are the numbers calculate based on area of 2X? If so, showing the area ratio to the whole are in table is more easy to understand the differences of the assembled quality. Please re-consider the style of figure/Table in this section.
P5 L5(right)The small red bar on the origin: They are really small and cannot see. Please change the layout.

Gene content:
P5, L33 (right) We align the S. tuberosum representative transcript sequences..

Did authors used transcript sequences registered SRA? Describe the source of the sequences

Library accuracy
P6, L61 (left) Dovetail data shows much smoother fragment distribution
Add the dovetail reads distribution in Fig.3.

Figure 6. I can see the letters of Y axis. Please change the font size. Please also specify the version of S. tuberosum reference in the legend.

Table 2. Add the sequence coverage in the table.

Methods

Are the methods appropriate to the aims of the study, are they well described, and are necessary controls included? Choose an item.

Conclusions

Are the conclusions adequately supported by the data shown? Choose an item.

Reporting Standards

Does the manuscript adhere to the journal's guidelines on minimum standards of reporting? Choose an item.

Choose an item.

Statistics

Are you able to assess all statistics in the manuscript, including the appropriateness of statistical tests used? Choose an item.

Quality of Written English

Please indicate the quality of language in the manuscript: Choose an item.

Declaration of Competing Interests

Please complete a declaration of competing interests, considering the following questions:

- Have you in the past five years received reimbursements, fees, funding, or salary from an organisation that may in any way gain or lose financially from the publication of this manuscript, either now or in the future?
- Do you hold any stocks or shares in an organisation that may in any way gain or lose financially from the publication of this manuscript, either now or in the future?

- Do you hold or are you currently applying for any patents relating to the content of the manuscript?
- Have you received reimbursements, fees, funding, or salary from an organization that holds or has applied for patents relating to the content of the manuscript?
- Do you have any other financial competing interests?
- Do you have any non-financial competing interests in relation to this paper?

If you can answer no to all of the above, write 'I declare that I have no competing interests' below. If your reply is yes to any, please give details below.

I declare that I have no competing interests

I agree to the open peer review policy of the journal. I understand that my name will be included on my report to the authors and, if the manuscript is accepted for publication, my named report including any attachments I upload will be posted on the website along with the authors' responses. I agree for my report to be made available under an Open Access Creative Commons CC-BY license (http://creativecommons.org/licenses/by/4.0/). I understand that any comments which I do not wish to be included in my named report can be included as confidential comments to the editors, which will not be published.

I agree to the open peer review policy of the journal

To further support our reviewers, we have joined with Publons, where you can gain additional credit to further highlight your hard work (see: https://publons.com/journal/530/gigascience). On publication of this paper, your review will be automatically added to Publons, you can then choose whether or not to claim your Publons credit. I understand this statement.

Yes Choose an item.
